# Metabolomic and high-throughput sequencing analysis—modern approach for the assessment of biodeterioration of materials from historic buildings

**DOI:** 10.3389/fmicb.2015.00979

**Published:** 2015-09-29

**Authors:** Beata Gutarowska, Sukriye Celikkol-Aydin, Vincent Bonifay, Anna Otlewska, Egemen Aydin, Athenia L. Oldham, Jonathan I. Brauer, Kathleen E. Duncan, Justyna Adamiak, Jan A. Sunner, Iwona B. Beech

**Affiliations:** ^1^Department of Biotechnology and Food Sciences, Institute of Fermentation Technology and Microbiology, Lodz University of TechnologyLodz, Poland; ^2^Department of Microbiology and Plant Biology, University of OklahomaNorman, OK, USA

**Keywords:** biodeterioration, historic building materials, electron microscopy, genomics, metabolomics

## Abstract

Preservation of cultural heritage is of paramount importance worldwide. Microbial colonization of construction materials, such as wood, brick, mortar, and stone in historic buildings can lead to severe deterioration. The aim of the present study was to give modern insight into the phylogenetic diversity and activated metabolic pathways of microbial communities colonized historic objects located in the former Auschwitz II–Birkenau concentration and extermination camp in Oświecim, Poland. For this purpose we combined molecular, microscopic and chemical methods. Selected specimens were examined using Field Emission Scanning Electron Microscopy (FESEM), metabolomic analysis and high-throughput Illumina sequencing. FESEM imaging revealed the presence of complex microbial communities comprising diatoms, fungi and bacteria, mainly cyanobacteria and actinobacteria, on sample surfaces. Microbial diversity of brick specimens appeared higher than that of the wood and was dominated by algae and cyanobacteria, while wood was mainly colonized by fungi. DNA sequences documented the presence of 15 bacterial phyla representing 99 genera including *Halomonas, Halorhodospira, Salinisphaera, Salinibacterium, Rubrobacter, Streptomyces, Arthrobacter* and nine fungal classes represented by 113 genera including *Cladosporium, Acremonium, Alternaria, Engyodontium, Penicillium, Rhizopus*, and *Aureobasidium*. Most of the identified sequences were characteristic of organisms implicated in deterioration of wood and brick. Metabolomic data indicated the activation of numerous metabolic pathways, including those regulating the production of primary and secondary metabolites, for example, metabolites associated with the production of antibiotics, organic acids and deterioration of organic compounds. The study demonstrated that a combination of electron microscopy imaging with metabolomic and genomic techniques allows to link the phylogenetic information and metabolic profiles of microbial communities and to shed new light on biodeterioration processes.

## Introduction

Identifying the conditions and causes governing biodeterioration of construction materials, in general, and heritage materials, in particular, is essential in aiding the development of effective prevention and mitigation strategies. Importantly, both disinfection and conservation treatments must be approached with the utmost caution. Incorrectly administered mitigation strategies may actually increase the severity of biodeterioration and result in the destruction of the materials they aim to protect (Pillinger et al., 2008; Saiz-Jimenez et al., 2012).

Characterizing microbial biodiversity and assessing the degree of material biodeterioration requires a combination of methods, such as modern molecular microbial ecology methods, advanced light and electron microscopy and chemical and surface analysis (González and Saiz-Jiménez, 2005; Dakal and Arora, 2012; Ettenauer et al., 2012). The use of non-invasive techniques is of extreme importance, to avoid damage to irreplaceable historic artifacts. Typically, only small specimen sizes are available for investigations (Sterflinger and Piñar, 2013). In addition, the deteriorated state of the material and the presence of microbial by-products often require extensive modifications to standard analytical procedures and methods (Dakal and Arora, 2012). The study reported here offers an example of a comprehensive approach to the analysis of biodeterioration phenomena, using construction materials from the buildings on the grounds of the former concentration and extermination camps, Auschwitz I and Auschwitz II–Birkenau (1940–1945) in Poland. These structures are of exceptional historic importance as memorials of the tragic events of World War II and as symbols for the Holocaust.

The two historic sites, from which wood and brick specimens were obtained, belong to the Auschwitz–Birkenau State Museum near Oswiecim in southern Poland. The museum covers a total area of 171 ha. It is responsible for the conservation of 155 barracks, approximately 300 relics, 35 watchtowers, four gas chamber and crematoria ruins, two water treatment plants, shelters, as well as many kilometers of railroad tracks, roads, irrigation/drainage ditches, sewers, fences, and gates. This impressive and extensive collection of historic structures requires continuous monitoring to mitigate the onsets of biodeterioration processes. Among the factors that favor severe biodeterioration of principal building materials used in the construction of the barracks, namely wood and brick, are the absence of heating within the buildings and the lack of effective protection against moisture. High humidity levels often measured inside the buildings also result from the geographical location of the site, i.e., its proximity to two rivers (Kościelniak et al., 2012). Currently, there is no public admission to some of the barracks in sections B I and B II of the museum because of their dilapidated conditions.

Owing to the unprecedented historic significance of the Auschwitz–Birkenau State Museum, intensive conservation work is currently in progress to preserve and make all barracks available to visitors.

The processes of biodeterioration are unique for each building material and, furthermore, vary with climatic conditions. In particular, little is known about the exact mechanisms of biodeterioration of wood and brick in historic buildings located in the temperate climate zone of the northern hemisphere, (e.g., countries in North and Central Europe, such as Poland). This climate is characterized by large fluctuations in temperature and humidity. Thus, temperatures can vary from: −30°C to +30°C and the relative humidity (RH) from 30 to >90%. This climate zone also experiences long wet seasons (spring, summer) and is subject to extreme weather events, such as floods and droughts. Such an environment promotes the growth of microorganisms that are tolerant to broad ranges of temperature and humidity.

The investigation reported herein expands on the previous studies that (i) assessed the visual signs of biodeterioration of wood and bricks from selected barracks and (ii) reported on isolation and identification, as well as seasonal variations in bacterial, fungal, cyanobacterial, algal, bryophyte and lichen populations associated with these materials (Koziróg et al., 2014; Piotrowska et al., 2014; Rajkowska et al., 2014). Although these early analyses revealed that wood and brick were in a severe state of deterioration, culture-dependent methods could not confirm unambiguously that the biotic component was a key factor contributing to their damage.

The aim of the present study was to a detailed characterization of the phylogenetic diversity and activated metabolic pathways of microbial communities inhabiting wood and brick collected from historic objects located in the former Auschwitz II–Birkenau concentration and extermination camp in Oświęcim by combining the techniques of electron microscopy imaging, elemental analysis and metabolomics with genomic profiling. Employing state-of-the-art ultra-high performance liquid chromatography (UPLC) coupled to high-resolution mass spectrometry (HRMS), and in particular to quadruple time-of-flight mass spectrometry (QToF-MS) (Lenhart et al., 2014; Brauer et al., 2015 and references therein), allowed the detection of active metabolic pathways present in heritage materials. To our knowledge, this is the first report on the use of UPLC/HRMS-based metabolomics in the study of biodeterioration of heritage materials. While DNA profiling of microbial communities demonstrated their considerable diversity, field emission electron microscopy (FESEM) imaging revealed the severity of material deterioration and confirmed the presence of microorganisms.

Importantly, combining the above methods has generated an unprecedented amount of chemical and phylogenetic data on biodeteriorating historical building materials and environments. The present study can thus demonstrate the potential usefulness of studied techniques for the degradation of historic buildings that will enable information-based approaches for aiding the design of effective and case-specific conservation strategies.

## Materials and methods

### Sampling

A total of eight wood and brick specimens were recovered from a brick barrack (B-124), and a wooden barrack (B-145), both of which are located in the former Auschwitz II–Birkenau concentration and extermination camp. Pictures of the buildings where the samples were collected are presented by Piotrowska et al. (2014). Descriptions of barracks and materials sampled are given in Table 1. Samples S1 and S2 (brick B124) were collected from the top surface layers of the brick, up to a depth of about 1 cm, while samples S3 and S4 (brick B124) were obtained from deeper layers, i.e., 8–16 cm below the brick surface. Wood samples S5 and S6 (wood B124) were collected from materials inside barrack B-124. Samples S7 and S8 (wood D2) were from materials stored inside barrack B-145. One of each of the two samples from the four locations (S2, S4, S6, and S8) was “activated,” i.e., exposed to high humidity (RH = 80%) at 28°C for 3 months, in order to promote microbial growth and to elucidate the effects of warm and humid conditions on materials integrity. Commercially obtained fired, solid brick (S9) (The Fired Brick Production “Konstatntynów” in Sanniki, Poland) and fresh pine wood segments (S10) (50 × 20 × 10 mm) served as controls.

**Table 1:** **Description of barracks and collected samples**.

**Sample number**	Sample site^*^	**Material**	**Analysis: M, metab.; G, genomic; F, FESEM**	**Barrack**	**Symptoms of biodeterioration**
S1 (brick B124)	Inside wall on the west side of the building; 0–1 cm behind brick surface; before activation	Brick	M/G/F	B124 brick barrack	Bulging and crumbling plaster, peeling paint, degradation of floor bricks
S2^**^ (brick B124)	Inside wall on the west side of the building; 0–1 cm behind brick surface; after activation	Brick	M/G/F		
S3 (brick B124)	Inside wall on the west side of the building; 8–16 cm behind surface; before activation	Brick	G/F		
S4^**^ (brick B124)	Inside wall on the west side of the building; 8–16 cm behind surface; after activation	Brick	G/F		
S5 (wood B124)	Plank bed in the center part of the building; before activation	Wood	M/G/F		Decomposition of wood in bunk beds and floor planks
S6^**^ (wood B124)	Plank bed in the center part of the building; after activation	Wood	M/G/F		
S7 (wood D2)	Exterior decking of the wooden barracks; historic material currently stored inside the building; before activation	Wood	M/G/F	B145 wooden barrack	The structural elements were affected by fiber splitting and cracking
S8^**^ (wood D2)	Exterior decking of the wooden barracks; historic material currently stored inside the building; after activation	Wood	M/G/F		
S9	N.A.	Modern brick	M/F	Retail	None
S10	N.A.	Modern pine wood	M/F	Retail	None

* All samples were collected on the 16th of October, 2013.

** Sample numbers S2, S4, S6, and S8 refer to samples that had been activated at RH 80% and 28°C for 3 months prior to analysis.

N.A.—not applicable.

### Field emission scanning electron microscopy (FESEM)

Scanning electron microscopy micrographs of wood and brick samples were acquired with a *Zeiss* SUPRA 55VP Ultra-High Resolution Scanning Electron Microscope. Images were obtained at magnifications between 649 × and 7.64 k ×, and at 1 kV for imaging.

### Analyses of microbial populations

#### DNA extraction

Small fragments of wood or brick specimens (0.5–1 g dry weight) were used for DNA extraction. Wood samples were shaved using a sterile scalpel. Brick fragments and wood shavings were ground into a fine powder under liquid nitrogen, using a mortar and pestle. DNA was extracted from 0.5 g wood powder and from 0.25 g brick powder, employing PowerBiofilm DNA extraction kit (MO-BIO Laboratories, Carlsbad, CA) following the manufacturer's instructions. Quantification of DNA was conducted using Qubit 2.0 Fluorometer, high sensitivity dsDNA assay as described in the manufacturer's protocol (Invitrogen/Life Technologies, Carlsbad, CA).

#### Bacterial and fungal PCR amplification

The bacterial 16S region was amplified for high-throughput sequencing analysis using universal prokaryotic primers, 519F and 806R (Wuchter et al., 2013), with the forward primer modified to contain the M13 sequence on its 5′ end (M13-519F). 16S rDNA amplification took place in 50 μL PCR reactions containing 5–10 μL DNA, 25 μL of 2 × Phusion high fidelity master mix, 10 pmol of M13-519F and 806R primers. The fungal ITS1 region was amplified for high-throughput sequencing analysis using ITS1FI2 forward (Schmidt et al., 2013) and ITS2 reverse (White et al., 1990) primers with the forward primer modified to contain the M13 sequence on its 5′ end (M13-ITS1F12). ITS amplification took place in 50 μL PCR reactions containing 5–10 μL DNA, 25 μL of 2 × DreamTaq Green master mix, 10 pmol of M13-ITS1FI2 and ITS2 primers and 0.5 M betaine. Amplification of bacteria and fungi was performed in an Eppendorf thermal cycler under the same conditions, where an initial denaturation at 98°C for 1 min was followed by 30 cycles of denaturation at 98°C for 10 s, annealing at 52°C for 20 s, extension at 72°C for 10 s, and a final extension at 72°C for 5 min. Bacterial and fungal PCR products were visualized on 1% (w/v) agarose gels pre-stained with SYBRSafe (Invitrogen, Carlsbad, CA). The images were recorded using a Gel Logic 112 Imaging System and Molecular Imaging Software v5 (Carestream, WoodBridge, CT).

#### High-throughput sequencing

Triplicate reactions of each bacterial and fungal PCR product were pooled and purified using Ampure beads (Agencourt Bioscience Corporation, MA, USA). Ten microliters of each purified PCR product was added to a second PCR reaction each containing specific 12nt barcodes attached to M13. Samples were “tagged” with barcoded primers by re-amplification for six cycles. Barcode sequences are provided in Tables S1, S2, Supplementary Materials. Tagged PCR products were pooled in equimolar concentrations, corresponding to 500 ng DNA/ml and sequenced on an Illumina MiSeq sequencer at Oklahoma Medical Research Foundation, (Oklahoma City, OK, USA). Raw data files in FASTQ format were deposited in NCBI Sequence Read Archive (SRA) with the study accession number SRP053328 under Bioproject number PRJNA274593. The bacterial 16S rRNA gene libraries and the fungal ITS libraries were pre-processed and analyzed using a combination of Trimmomatic 0.32 (Bolger et al., 2014), the USEARCH v7 (UPARSE) pipeline (Edgar, 2013), and the bioinformatics software package QIIME version 1.8.0 (Caporaso et al., 2010). Briefly, Illumina adapters, low quality regions of sequence reads (Phred score < 25 over a 50 bp window), and sequences containing homopolymer regions (>6 bp) were eliminated from the paired-end sequence read files using Trimmomatic 0.32 (Bolger et al., 2014). Paired-end reads that passed this quality-filtering step were joined together using the EA-Utils package, fastq-join (Aronesty, 2011) and analyzed further.

USEARCH v7 (UPARSE) was used for filtering the chimeric sequences and clustering OTUs (Edgar, 2013) and QIIME was used for beta-diversity analyses. No barcode mismatches were allowed. Sequences were demultiplexed using split_libraries_fastq.py script in QIIME (Caporaso et al., 2010). Duplicate sequences were removed with “derep_fulllength” command and single reads were eliminated by “sortbysize” command. Sequences were clustered based on 97% identity and an OTU table was created using the nonchimeric sequences. The Ribosomal Database Project classifier (Wang et al., 2007) against the Greengenes database (97% taxonomy) (2012 release: http://greengenes.lbl.gov; McDonald et al., 2012) was used for taxonomy assignment of bacteria. UNITE database (2014 QIIME release: http://unite.ut.ee/repository.php; Kõljalg et al., 2013) was used for taxonomy assignment of fungi. Beta diversity analyses were executed on rarefied data using jackknifed_beta_diversity.py script in QIIME (Caporaso et al., 2010) and Principal Coordinate Analyses (PCoA) were performed on weighted and unweighted unifrac calculations.

### Metabolomic survey

One gram of ground brick, or 0.20 g of ground wood, was added to 4.0 mL of 4 N HCl and the suspensions sonicated in a water bath for 30 min. The mixtures were extracted into 2 times 3 mL ethyl acetate (HPLC grade, Sigma Aldrich). The organic phase was evaporated to dryness under a constant flow of ultrapure nitrogen gas and the residue reconstituted in 100 μL isopropanol (Sigma Aldrich). Volumes of 5 μL were injected into the UPLC. Analysis of each sample was performed in triplicate and *p*-value of 0.01 was used for all abundance comparisons between sets of triplicates.

An UPLC/HRMS system, i.e., an Agilent 1290 Infinity UPLC coupled to an Agilent 6538 quadrupole time-of-flight mass spectrometer, was used for metabolomic analysis. Samples were processed in both negative and positive ion mode. When the mass spectrometer was operated in negative ion mode, an Acquity UPLC® HSS C18 SB column (1.8 μm, 2.1 × 100 mm, Waters, Ireland) was used for separation. The flow rate was 0.4 mL/min and a linear gradient from 23.5 to 95.5% HPLC- grade acetonitrile over 35 min, followed by 5 min at 95.5%, was used. With positive ion mode mass spectrometry, a SeQuant® ZIC®-HILIC column (5 μm, 150 × 4.6 mm, The Nest Group, Inc., Mass., USA) was used with a flow rate of 0.3 mL/min. A linear gradient from 80 to 20% acetonitrile was used for the first 30 min, followed by 5% acetonitrile for an additional 8 min. Solvents contained 0.1% formic acid to promote positive ion formation in the electrospray. For both positive and negative ion mode, MS parameters were as follow: ion-source gas temperature, 350°C; capillary voltage, 3500 V; fragmentor voltage, 160 V; m/z range, 50–1100; data acquisition rate, 4 GHz; and 1 spectrum recorded per second.

Raw MS data was processed using IDEOM version 19 (Creek et al., 2012a) workflow. IDEOM processing utilizes XCMS Centwave (Tautenhahn et al., 2008) for peak detection and mzMatch, R (Scheltema et al., 2011) for peak alignment between triplicates and between samples, for filtering and for the storage of the data in peakML formatted files. Feature alignment was performed with a retention time window of 0.5 min and a mass error window of 5 ppm. Scripts for XCMS (Smith et al., 2006) and mzMatch (Scheltema et al., 2011) are coded in the R environment.

Alignment of detected peaks was performed separately for the set of brick and the set of wood samples. In this procedure, peaks obtained in different UPLC/HRMS experiments are determined to be formed from the same compound, based on their appearance at nearly the same retention time and m/z value. After identifying isotope peaks and assigning them to their respective parent peak, the procedure results in a list of compounds, each with an associated mass and retention time. Compounds that have thus been detected, but not identified, are customarily referred to as “features,” and this term will be used throughout the manuscript.

Detected features were matched against the IDEOM's version of the Kyoto Encyclopedia of Genes and Genomes (KEGG) metabolite database (Kanehisa et al., 2014) using a mass tolerance of 4 ppm. For features detected in positive ion mode (about 80% of the total) identification was based also on retention times. The retention time calculator for HILIC chromatography, developed by Creek et al. (2011) and implemented in IDEOM was used. The calculator uses a quantitative structure/retention relationship model that incorporates six physicochemical variables in a multiple-linear regression scheme based on 120 authentic standard metabolites. As suggested by Creek et al. (2012b), the maximum difference between calculated and observed RT was set to 10% for the authentic standards and to 45% for all other metabolites. The use of the RT calculator serves to remove a significant number of false positive identifications. In the present study, 33 metabolite authentic standards were used to calibrate the retention time predictor (Table S7, Supplementary Materials). Twelve of these were detected in the brick and wood samples and these are shown in bold text in Table S7.

After the putative identification of extracted features with metabolites and other compounds in the IDEOM database, the metabolites were matched with the Pathos (Leader et al., 2011) metabolic pathway database. The definitions of the distinct pathways in Pathos are identical to that used for the KEGG database.

## Results

### FESEM imaging of historical materials

**Figure 1:**
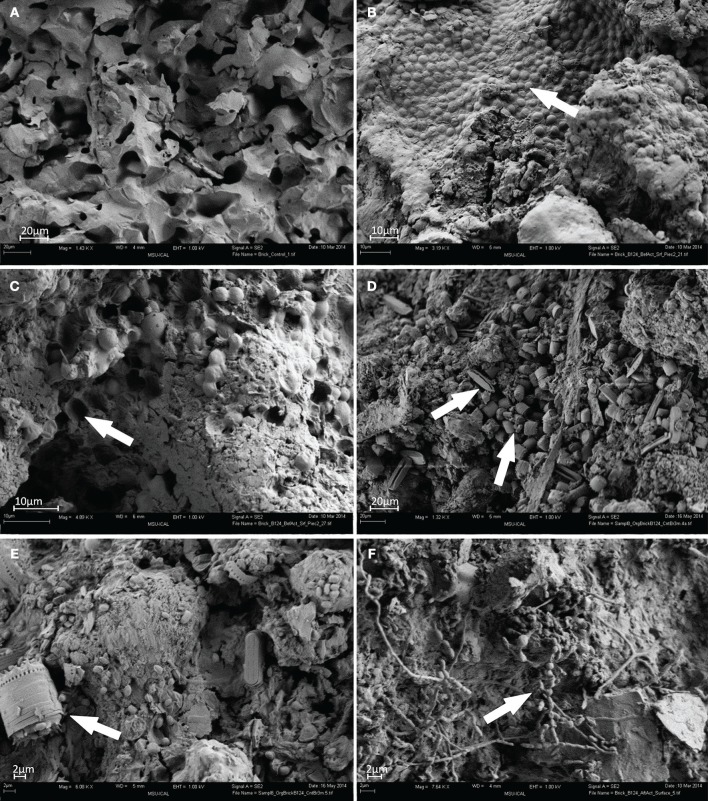
**FESEM images of brick samples: (A) control brick (S9); (B–E) brick sample B124 before activation (S1); (F) brick sample B124 after activation (S2) (Mag. 1.32–7.64 K ×), viewing distance 4–6 mm**.

FESEM imaging of test specimens revealed striking differences between surfaces of the control brick (Figure 1A) and those recovered from the barrack located in the former Auschwitz–Birkenau concentration and extermination camp (Figures 1B–F). No visual differences in colonization or of degradation patterns between replicate samples were perceived and the images presented are representative of all biological replicates. The surface of the control brick had a porous structure of an apparently uniform material, with no apparent signs of degradation or of microbial contamination (Figure 1A). In contrast, the historical brick had experienced extensive microbial colonization and undergone considerable changes in their topology and morphology. Clusters of numerous spherical cyanobacterial cells, embedded in layers of deposits, covered large parts of the test brick (Figure 1B, arrow). The removal of cyanobacterial cells and their associated deposits revealed arrays of μm-sized pits in the supporting structure (Figure 1C, arrow). These are possibly caused by the release of acidic metabolites, which led to localized etching of the material. Similar pits, caused by *Gloeocapsa* cyanobacteria, were seen by Gaylarde and Englert (2006) in the deteriorated quartzite of a church in Minas Gerais, Brazil. However, cyanobacteria belonging to *Gloeocapsa* (*G. biformis, G. kuetzingiana, G. rupestris, G. magma, G. punctata, G. livida*) are common inhabitants of building, temples, monuments surfaces and was found on rock, limestone, marble, sandstone, wall paintings and painted or coated surfaces (Gaylarde and Gaylarde, 2002; Nugari et al., 2009; Caneva et al., 2012). Abundant aerophilic diatoms identified, based on their morphology, as belonging to the genera *Diadesmis, Stephanodiscus*, and *Nitzschia* were also detected (Figure 1D, arrow). Furthermore, single bacterial cells sized 2–5 μm were present on the surfaces of the brick and associated with diatom cells (Figure 1E, arrow).

Surfaces of activated brick B124 (S2) revealed extensive development of actinomycetes as evidenced by characteristic hyphae of a diameter of approx. 1 μm, and the formation of spores (Figure 1F, arrow).

**Figure 2:**
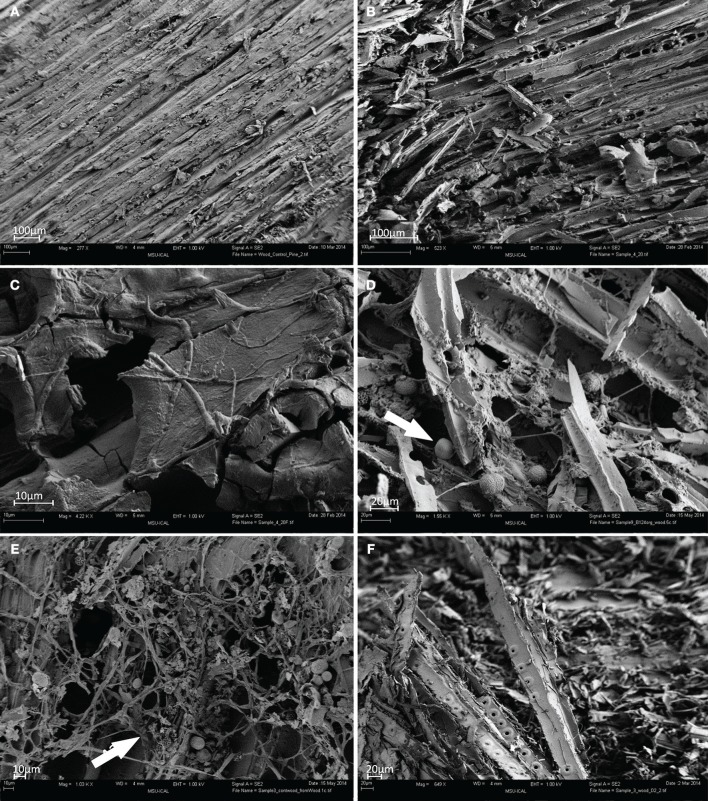
**FESEM images of wood samples. (A)** Control pine wood (S10); **(B–D)** wood sample B124 before activation (S5); **(E)** wood sample B124 after activation (S6); **(F)** wood sample B145 before activation (S7) (Mag. 649–4.22 K ×).

The surface of heritage wood B124 (S5) showed extensive degradation (Figure 2B) when compared to the control wood (Figure 2A). The penetration of pseudomycelium into the wood matrix is the most probable cause of the observed structural damage (Figure 2C). Fungal spores and bacterial cells are also apparent, although their contribution to material deterioration is not explicit (Figure 2D, arrow). The structure of wood D2 (S7) was more severely degraded than that of the wood B124 (S5) (Figure 2F).

Following activation, abundant proliferation of actinomycetes was observed on surfaces of wood B124 (S6), with wood fibers entwined and separated from each other by pseudomycelium (Figure 2E, arrow). Numerous spores, as well as active growth of fungal mycelium in the form of hyphae, were apparent and are depicted in the latter figure.

### Microbial diversity characterized by illumina sequencing

**Figure 3:**
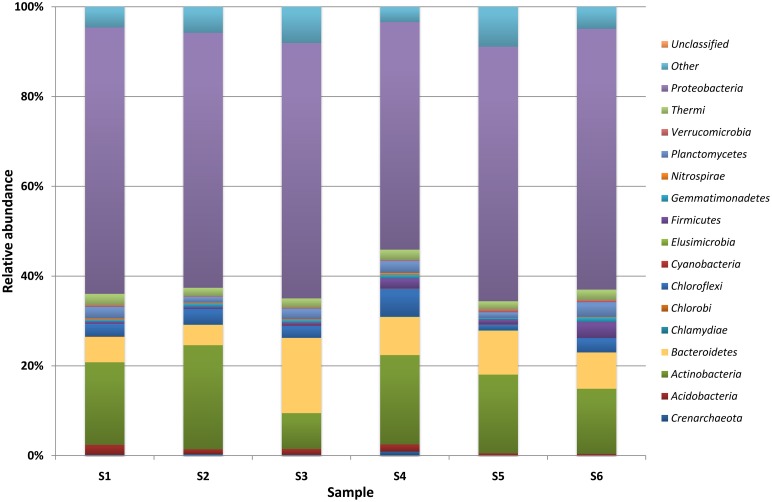
**Percentage share of each bacterial/archaeal phylum making up the total number of identified strains colonizing museum objects**.

**Figure 4:**
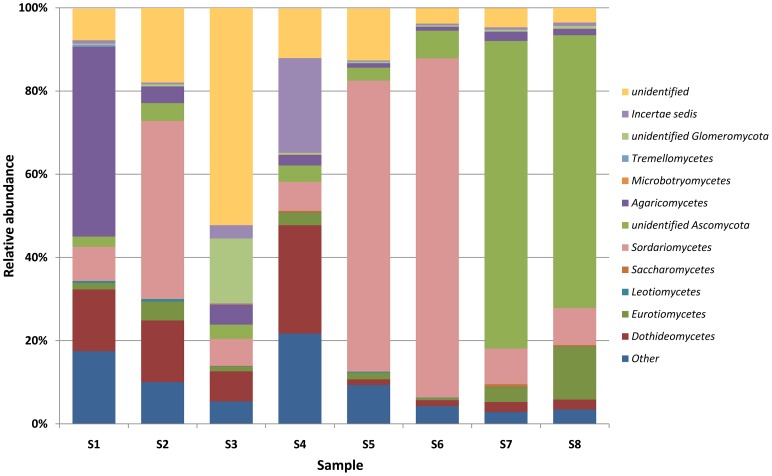
**Percentage share of each fungal classes making up the total number of identified strains colonizing museum objects**.

DNA sequencing data revealed the presence of a total of 99 genera of bacteria, belonging to 15 different classes (Table S3, Supplementary Materials) and 113 genera of molds from 9 classes (Table S4, Supplementary Materials) in the brick and wood samples. Sequences of *Proteobacteria* were dominant in brick B124 both prior to and following the activation process (Figure 3) and constituted from 50 to 59% of OTUs. The genera *Marinobacter* (4.3–25.5%), *Halomonas* (1.5–2.7%) *Halorhodospira* and *Salinisphaera* (below 0.1%) represented the halophilic bacteria. DNA sequences associated with halophilic and halotolerant bacteria of the genera *Salinibacterium* and *Rubrobacter* were also identified in the class *Actinobacteria*. This latter group was dominated by sequences representing the genera *Arthrobacter* (1.0–6.5%) and *Pseudonocardia* (0.45–4.1%) (Table S3, Supplementary Materials). There was an increase in the percentage of bacterial DNA sequences after the specimen activation process, i.e., upon incubation of samples at high humidity and temperature. Bacterial and fungal community profiles in the studied brick samples varied with the activation regime and the location of the sample (Table S4, Supplementary Materials). The as-received sample of the brick was dominated by fungal DNA sequences of the class *Agaricomycetes* and the genus *Volvariella* (0.25–4.2%), while *Sordariomycetes* was the most abundant class in the activated specimen (Figure 3), with the genus *Engyodontium* dominating this class and representing 9.2% of the total fungal OTU pool in this specimen. The sample yielded a high proportion of sequences characteristic of fungi recognized as potential human allergens, namely of the genera *Cladosporium* (1.6–5.6%) and *Alternaria* (0.9–8.3%) and the class *Dothideomycetes*, (Figure 4). The DNA recovered from wood B124 (S5) represented mostly *Proteobacteria*, with a dominance of halophilic organisms of the genera *Marinobacter* (4.3–5.2%) and *Halomonas* (1.7–2.7%), as well as *Devosia* (3.7–5.6%) and *Chelativorans* (0.5–1.4%) (Table S4, Supplementary Materials). The activation of the wood sample increased the abundance of sequences representative of the genus *Promicromonospora*, of the class *Actinobacteria*. A high fungal diversity was associated with wood B124 (S5) (Figure 4). The sample was dominated by molds of the genera *Alternaria* and *Cladosporium* (class *Dothideomycetes*), *Penicillium* (class *Eurotiomycetes*) and *Engyodontium* (class *Sordariomycetes*). The fungal species composition was not altered by specimen activation.

In the case of wood D2 (S7), bacterial DNA amplification proved to be unsuccessful. Solely fungal sequences were obtained. Prior to the activation, the DNA recovered from the surface of wood D2 (S7) was dominated by sequences of molds from the genera *Emerciella* (1.05%) and *Penicillium* (1.65%), belonging to the class *Eurotiomycetes, as well as Alternaria* (0.4%) and *Cladosporium* (0.6%) of the class *Dothideomycetes*, and *Engyodontium* of the *Sordariomycetes* class (0.45%) (Figure 4, Table S4, Supplementary Materials). After incubation at increased humidity and temperature, the percentages of the majority of the identified mold taxa remained at the same level. Fungi of the genus *Penicillium* were the exception, as their number increased more than six-fold.

### Metabolic pathways analysis of historical materials

A large number of features were obtained from the analysis of the UPLC/HRMS data. (See images 3 to 7 in Supplementary Materials for total ion chromatograms and selected extracted ion chromatograms). However, only those with relative abundance higher than 0.1% in at least one sample were retained for further analysis. This resulted in the removal of about half of the detected features. After this filtering, the number of features used for the metabolic analysis presented here was 3069 for wood samples (S5, S6, S7, S8, and S10) and 2908 for brick samples (S1, S2, and S9). For the purpose of this discussion, a feature is considered not to be “detected” in a particular sample if its relative abundance in that sample was less than 0.1%.

Overall, the abundances of features were significantly higher in the activated brick sample (S2) than in the non-activated ones (S1, S9). For example, of the 2908 features detected in the combined brick samples, 90% were detected in the activated sample and 36% in the non-activated ones. The same trend, though less pronounced, was observed for the activated wood samples (S6, S8) vs. the non-activated ones (S5, S7).

Of the features detected in the wood and brick samples, 43 and 35%, respectively, were putatively identified by matching them with metabolites and other compounds listed in the KEGG database, as described in Materials and Methods. For the more than 80% of these that were detected in positive ion mode, the identification was based both on exact mass and on a retention time calculator that had been calibrated for the instrument using 33 internal standards, see Materials and Methods. The remaining identifications were based on exact mass only, but these putative metabolites made an insignificant contribution (about 3% of identifications) to the results presented here. The putatively identified metabolites in the brick and wood samples are listed in Table S6, Supplementary Materials. The implementation of the RT calculator resulted in the removal of about 1000 putative metabolite identifications for the wood samples and about 500 for the brick samples. The large majority of these are expected to have been false positives.

The putative metabolites were used for metabolic pathway analysis as described in Materials and Methods. Ideally, the metabolites used for the analysis should all be identified at Level 1 (Sumner et al., 2007). Unfortunately, with existing technology, this is not yet a realistic goal for most studies. However, the work by Creek et al. (2011) demonstrates that pathway analysis is possible at Level 2 identifications. Indeed, in extensive work on environmental metabolomics on complex microbial systems, we have found that pathway analysis based on metabolites identified on the basis of exact mass and retention time only, very often but not always, correctly identifies up- and down-regulated pathways. One reason for this degree of success is that errors in metabolite identifications tend to cancel out when the identification of an active pathway is based on a large number of metabolites. For these reasons and with these caveats in mind, the results discussed below should be dealt with carefully. They demonstrate, however, what is possible and they can be used for formulating hypotheses or for inspiring new experimental approaches on a very important problem.

A total of 22 pathways, including both primary and secondary metabolism, were “detected” on the basis of a significant number (≥5) of detected, putative metabolites. These pathways are strong candidates for being active in the respective samples, and they are listed in Table 2. For each pathway, the number of metabolites detected is indicated, as well as the number of “changed” metabolites, i.e., those whose abundances before and after activation differed by a factor of more than 2. With one exception, all those metabolites in all listed pathways were upregulated in the activated samples. The exception was observed for pathway #7 in Table 2 (“*Biosynthesis of unsaturated fatty acids”*) for which all metabolites were observed with a lower abundance in the activated wood samples (S6, S8) than in the non-activated ones (S5, S7). In addition to the 22 pathways listed in Table 2, an additional 45 pathways were “detected.” While these are listed in Table S5 in Supplementary Materials, it should be noted that the reliability of detection was lower for the pathways not listed in Table 2.

**Table 2:** **Metabolic pathways detected in brick and wood samples**.

**No.**	**Metabolic pathway**	Brick^*^	Wood B124^*^
1	Alanine, aspartate, glutamate metabolism		7/39 (5 ↑)
2	Alpha-linolenic acid metabolism	8/24 (7 ↑)	
3	Arachidonic acid metabolism		10/74 (10 ↑)
4	Arginine and proline metabolism	13/80 (13 ↑)	9/80 (6 ↑)
5	Benzoate degradation	5/23 (4 ↑)	
6	Biosynthesis of type II polyketide products		9/86 (6 ↑)
7	Biosynthesis of unsaturated fatty acids	7/49 (5 ↑)	**6/49 (6 ↓)**
8	Carotenoid biosynthesis		5/91 (5 ↑)
9	Diterpenoid biosynthesis	11/69 (8 ↑)	20/69 (12 ↑)
10	Flavonoid biosynthesis		11/68 (11 ↑)
11	Glucosinolate biosynthesis	7/72 (5 ↑)	
12	Isoquinoline alkaloid biosynthesis	7/93 (6 ↑)	12/93 (3 ↑)
13	Nicotinate and nicotinamide metabolism	8/46 (6 ↑)	
14	Phenyloalanine metabolism	19/64 (16 ↑)	10/64 (4 ↑)
15	Phenyloalanine, tyrosine, tryptophan biosynthesis	6/31 (6 ↑)	
16	Phenylpropanoid biosynthesis	11/51 (9 ↑)	12/51 (7 ↑)
17	Steroid biosynthesis	7/99 (5 ↑)	8/45 (4 ↑)
18	Tropane, piperidine, and pyridine alkaloid biosynthesis	9/61 (8 ↑)	
19	Tryptophan metabolism	21/80 (20 ↑)	
20	Tyrosine metabolism	17/75 (16 ↑)	21/75 (8 ↑)
21	Ubiquinone and other terpenoid-quinone biosynthesis	10/36 (9 ↑)	11/36 (8 ↑)
22	Valine, leucine, isoleucine biosynthesis	5/23 (5 ↑)	

* The number of metabolites participating in the metabolic pathway is indicated on the right hand side of the slash, and the number of these that were (putatively) detected is indicated on the left hand side.

The number within parenthesis indicates the number of metabolites that were detected with at least a two-fold higher (↑) or a two-fold lower (↓) abundance in the activated sample as compared to the non-activated sample. A p-value of 0.01 was used to determine if a two-fold change was statistically significant or not. A metabolite is considered “detected” in a sample, if its relative abundance is at least 0.1%. The pathways are classified as follows: 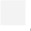 pathways typical for active primary metabolism of aminoacids, vitamins, glycerophospholipids, sulfur and nitrogen; 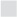 pathways typical for active primary metabolism of organic acids including fatty acids and steroids; 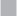 secondary metabolism pathways; 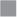 pathways typical for active primary metabolism of dyes and aromatic compounds; 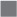 pathways typical for active primary metabolism of phototrophs (CO_2_ assimilation, chlorophyll degradation); 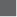 pathways involved in degradation of compounds which may originate from herbicides, insecticides, biocides and other compounds used to preserve historic materials. Downregulated pathway are represented in bold.

Active metabolic pathways can be illustrated in KEGG metabolic graphs by highlighting metabolites that are detected in a sample. Examples of such maps for the brick and wood samples, using the KEGG ipath map, are depicted in Images 1, 2 (Supplementary Materials).

The majority of the metabolic pathways detected in the heritage brick and wood specimens (Table 2 and Table S5, Supplementary Materials) are pathways that are active during growth of all living organisms. The detected pathways include the biosynthesis and degradation of amino acids, such as alanine, lysine, methionine, cysteine, arginine, glutamic acid, aspartic acid, tryptophan, phenylalanine, tyrosine, valine, leucine, and isoleucine; the biosynthesis of vitamins and peptides; biosynthesis of glycosaminoglycans, glycerophospholipids, and glycosphingolipids forming biological membranes; sulfur and nitrogen metabolic pathways; typical primary metabolic pathways of glycolysis, pentose-phosphate cycle and the tricarboxylic acid cycle (TCA); metabolic pathways of compounds involved in cellular respiration (NAD, NADP, coenzyme Q10), the synthesis of nucleotides and other compounds.

Eleven pathways were associated with the metabolism and synthesis of fatty acids and steroids. Moreover, two pathways, namely those involved in CO_2_ assimilation and degradation of porphyrins and chlorophyll, were identified in the brick specimen, which indicates the possible presence of phototrophic organisms. Seven pathways found in the analyzed samples indicated the biosynthesis of aromatic compounds including sesquiterpenes and diterpenes. Secondary metabolites, in particular a metabolite representative of the streptomycin pathway, were detected in the wood sample. Compounds such as benzoates, naphthalene, xylene, and ethylbenzene, which are indicative of degradation of organics, were present in both wood and brick specimens (Table 2 and Table S5, Supplementary Materials).

## Discussion

Electron microscopy imaging and phylogenetic analysis of selected heritage wood and brick material revealed their extensive colonization by diverse microorganisms, comprising fungi, algae and bacteria. Microalgae (diatoms) and cyanobacteria, actinomyces and fungi dominated the surface of brick B124 (S1), while actinomycetes and fungi grew readily on wood.

DNA sequences representing 99 bacterial genera belonging to 15 classes and fungi from 113 genera belonging to nine classes were identified in the tested samples. In brick samples 94 bacterial taxa and 97 fungi were detected, while in wood 84 bacterial genera and only 54 fungi were found. Previous investigation of building material from the former Auschwitz II–Birkenau concentration and extermination camp carried out using conventional methods indicated much lower microbial diversity than that revealed with Illumina sequencing. In the former studies only a total of 8 genera of bacteria belonging to 3 classes and 23 fungal genera of 9 classes were identified (Piotrowska et al., 2014; Rajkowska et al., 2014). The current investigation confirmed earlier reported results by demonstrating the presence of DNA sequences matching those of bacteria detected through cultivation, namely the genera *Arthrobacter, Bacillus, Sporosarcina*, and *Streptomyces* and fungi of the genera *Cladosporium, Acremonium, Alternaria, Engyodontium, Penicillium, Rhizopus*, and *Aureobasidium*. Unlike enrichments, genomic analysis revealed halophilic and halotolerant bacterial sequences representing genera *Halomonas, Halorhodospira, Salinisphaera, Salinibacterium, and Rubrobacter*. The biodeterioration potential of these genera has been described and discussed by Ortega-Morales et al. (2004), Piñar et al. (2009, 2013) and Ettenauer et al. (2014). Bacteria of the genera *Streptomyces, Nocardia, Arthrobacter*, and *Micromonospora* sequences of which were found in bacterial DNA isolated from test specimens have all been associated with and implicated in the destruction of heritage materials, especially murals and layers of paint (Schabereiter-Gurtner et al., 2001, 2004; Stomeo et al., 2007; De Felice et al., 2010; Abdel-Haliem et al., 2013), wooden figures (Lupan et al., 2014) and church walls (Pangallo et al., 2007).

Fungal sequences identified in DNA recovered from tested brick and wood samples, comprised genera of molds isolated from heritage materials, such as archaeological and historic wood (Zyani et al., 2009), sculptures and statues (Ebrahimi et al., 2011), murals and stained glass windows (Valme et al., 2008) and material-deteriorating capability of these organisms has been evidenced and emphasized.

The advantage of using Illumina next-generation sequencing method (NGSM) over conventional microbiological techniques in the study of phylogenetic diversity of microbial populations associated with deteriorating heritage materials is undisputable. The NGSMs are increasingly employed to characterize bacterial and fungal community structure in soil (Schmidt et al., 2013), compost (Neher et al., 2013), oilfield environments (Lenhart et al., 2014), animal and human gut (Wagner Mackenzie et al., 2015), marine and freshwater habitats (Fang et al., 2014), as well as cultural heritage materials (Cutler et al., 2013; Rosado et al., 2014). Notwithstanding their popularity this method has not yet been used to assess the biodiversity of microorganisms associated with deterioration of historic materials. The clone library method has previously been used in the analysis of bacterial colonization of historic building materials from the Auschwitz–Birkenau State Museum (Otlewska et al., 2015). The study demonstrated the presence of 61 different bacterial taxa, including many species reported as pertinent to degradation processes (Otlewska et al., 2015 and references therein). The present study confirms that NGSM is a very useful and, comparing with culturing methods and clone library approach, a superior tool for interrogating the diversity of the microbial populations associated with heritage materials.

Within the 3 months of activating brick B124 specimen by laboratory exposure to high humidity and temperature, material-specific microbial succession was observed, analogous to that reported in the field studies. The growth of autotrophic pioneering organisms such as algae and cyanobacteria was followed by that of actinomycetes and heterotrophic fungi. The succession phenomenon is readily accepted and documented in the literature (Herrera et al., 2004; Crispin and Gaylarde, 2005). The metabolomic analysis using UPLC/HRMS coupled to HRMS demonstrated the presence in the building material samples of many primary metabolic pathways characteristic of living organisms, consistent with the development of various groups of microorganisms. The indicated metabolic pathways included CO_2_ assimilation, characteristic of algae and cyanobacteria, as well as degradation of photosynthetic pigment (chlorophyll), most likely caused by heterotrophic microorganisms. FESEM images confirmed severe damage of test specimens and the formation of microbial biofilms. It has been proposed that the presence of biofilms on building materials may lead to changes in their physical and chemical properties and contribute to the biodeterioration of the materials (Gaylarde and Morton, 1999; Herrera et al., 2004). Dead cells of cyanobacteria and algae, both of which are considered to be primary colonizers of bricks, can serve as a source of nutrients for subsequent colonizers i.e., heterotrophic bacteria and fungi.

Loose fragments of degraded wood and crumbling brick observed in FESEM images may result from microbial metabolic activity. Organic acids can deteriorate building materials by contributing to mass loss through material dissolution and the formation of soluble or insoluble salts (Gomez-Alarcon et al., 1994; Warscheid and Braams, 2000). Studies by Ortega-Calvo et al. (1991), Gaylarde and Morton (1999), and Crispin and Gaylarde (2005) documented the growth of algae and cyanobacteria of the genera *Apatococcus, Chlorella, Chlorococcum, Klebsormidium, Trebouxia, Stichococcus* and *Lyngbyga, Chroococcus, Scytonema, Nostoc, Microcoleus, Plectonema* on the surfaces of buildings was accompanied by the production of organic acids. The secretion of 2-ketogluconic acid which initiates decomposition of silica by bacteria of the genera *Bacillus, Arthrobacter*, and *Streptomyces* has also been described (Papida et al., 2000; Warscheid and Braams, 2000) and acidification of building materials by fungi of the genera *Aspergillus, Penicillium* has been reported in a number of studies (Gu et al., 1998; Warscheid and Braams, 2000; Gutarowska and Czyżowska, 2009). The presence of bacterial and fungal DNA sequences belonging to the above genera was confirmed in microbial community analyses of wood and brick samples. Specifically, acidic compounds indicative of the TCA were identified in the metabolome of both brick and wood samples (Table S5, Figures S1, S2 Supplementary Material).

However, it should be noted that the observed damage to wood specimens can also result from physical disruption of the wood fibers, caused by the penetration of the fungal hyphae into the wood matrix (Crispin and Gaylarde, 2005) or by the activity of lignin and cellulose degrading enzymes (Blanchette, 2000).

The release of secondary metabolites, in particular by molds, including mycotoxins which are dangerous to human health, has been reported on the surfaces of building materials (Flannigan et al., 2001). The detection of streptomycin biosynthesis pathway would, if confirmed by accurate identifications, be consistent with the presence of *Actinomycetes*, demonstrated here not only with NGS (Table S3, Supplementary Material) but also through FESEM imaging (Figure 2E). The detection of compounds such as benzoate and the putative degradation pathways of naphthalene, xylene and ethylbenzene in the wood specimens documents past remedial treatments. These compounds originate from tars or paints which are frequently used to protect the wood against microbial growth. In addition the presence of degradation pathways of atrazine, which is a known herbicide, and of the insecticide benzoxazinone (Carretti et al., 2010; Giorgi et al., 2010) would indicate their progressive decay in the brick and wood. Both are used to control weeds and insects on the grounds of the Auschwitz–Birkenau museum.

Metabolomic profiling has not been previously been employed in the study of biodeterioration of building materials. However, the method has been used to screen and compare profiles of chemical compounds produced by molds and higher fungi in mono- and co-cultures on solid growth media (Peiris et al., 2008; Bertrand et al., 2013). These latter studies yield valuable information on species interactions. Unlike model laboratory cultures, environmental samples such as historic building materials, exhibit high biodiversity. Here reported analysis of metabolites using UPLC/HRMS is the first of its kind. The detection of metabolic pathway may be of great interest when developing novel conservation strategies. Metabolomic analyses do not require large quantities of samples, which is particularly important in the case of heritage material. The results of the metabolomic analysis complemented and further supported information on microbial communities obtained through FESEM imaging and NGS data.

### Conflict of interest statement

The authors declare that the research was conducted in the absence of any commercial or financial relationships that could be construed as a potential conflict of interest.
