# Supplementary material for: Metabolomic and high-throughput sequencing analysis—modern approach for the assessment of biodeterioration of materials from historic buildings
Source: Front Microbiol. 2015 Sep 29;6:979. doi: 10.3389/fmicb.2015.00979 (PMC4586457; doi:10.3389/fmicb.2015.00979)
Supplement: Supplementary file 1 [file Table1.DOCX]

**Table S1.** 16S rDNA barcode sequences for brick and wood samples

| Sample number | Sample description | Replicate # | Barcode Sequence | Linker Primer Sequence  (Variable spacer + M13) | Community |
| --- | --- | --- | --- | --- | --- |
| S1 | Brick B124, 0-1 cm, before activation | 1 | TTCCTAGGCCAG | CCCGTAAAACGACGGCCAG | Bacteria - Archaea |
|  |  | 2 | GCCGTCTCGTAA | CCCGTAAAACGACGGCCAG |  |
| S3 | Brick B124, 8-16 cm, before activation | 1 | CGTAGGTAGAGG | CCCGTAAAACGACGGCCAG | Bacteria - Archaea |
|  |  | 2 | TTCTGAGAGGTA | CCCGTAAAACGACGGCCAG |  |
| S2 | Brick B124, 0-1 cm, after activation | 1 | GTCGAATTTGCG | CCCGTAAAACGACGGCCAG | Bacteria - Archaea |
|  |  | 2 | AGGGTGACTTTA | CCCGTAAAACGACGGCCAG |  |
| S4 | Brick B124, 8-16 cm, after activation | 1 | ATTGTTCCTACC | CCCGTAAAACGACGGCCAG | Bacteria - Archaea |
|  |  | 2 | CTATCATCCTCA | CCCGTAAAACGACGGCCAG |  |
| S5 | Wood B124, before activation | 1 | ATTTAGGACGAC | CCCCGTAAAACGACGGCCAG | Bacteria - Archaea |
|  |  | 2 | ATCCCTACGGAA | CCCCGTAAAACGACGGCCAG |  |
| S6 | Wood B124, after activation | 1 | GCATCAGAGTTA | CCCCGTAAAACGACGGCCAG | Bacteria - Archaea |
|  |  | 2 | GACTTCATGCGA | CCGTAAAACGACGGCCAG |  |
