# Supplementary material for: Metabolomic and high-throughput sequencing analysis—modern approach for the assessment of biodeterioration of materials from historic buildings
Source: Front Microbiol. 2015 Sep 29;6:979. doi: 10.3389/fmicb.2015.00979 (PMC4586457; doi:10.3389/fmicb.2015.00979)
Supplement: Supplementary file 2 [file Table2.DOCX]

**Table S2.** ITS1 region barcode sequences for brick and wood samples

| **Sample number** | **Sample description** | **Replicate #** | **Barcode Sequence** | **Linker Primer Sequence**  **(Variable spacer + M13)** | **Community** |
| --- | --- | --- | --- | --- | --- |
| S1 | Brick B124, 0-1 cm, before activation | 1 | TGGTTGGTTACG | CCGTAAAACGACGGCCAG | Fungi |
|  |  | 2 | GTGTTCCCAGAA | CCGTAAAACGACGGCCAG |  |
| S3 | Brick B124, 8-16 cm, before activation | 1 | TGGCTTTCTATC | CCCGTAAAACGACGGCCAG | Fungi |
|  |  | 2 | GACTACCCGTTG | CCCGTAAAACGACGGCCAG |  |
| S2 | Brick B124, 0-1 cm, after activation | 1 | GCAGATTTCCAG | CCCGTAAAACGACGGCCAG | Fungi |
|  |  | 2 | CGATAGGCCTTA | CCCGTAAAACGACGGCCAG |  |
| S4 | Brick B124, 8-16 cm, after activation | 1 | CTGAAGGGCGAA | CCGTAAAACGACGGCCAG | Fungi |
|  |  | 2 | ACTGATGGCCTC | CCCCGTAAAACGACGGCCAG |  |
| S5 | Wood B124, before activation | 1 | GTCGTCCAAATG | CGTAAAACGACGGCCAG | Fungi |
|  |  | 2 | CCGAGGTATAAT | CGTAAAACGACGGCCAG |  |
| S6 | Wood B124, after activation | 1 | CGCTCACAGAAT | CGTAAAACGACGGCCAG | Fungi |
|  |  | 2 | TTCGATGCCGCA | CCCGTAAAACGACGGCCAG |  |
| S7 | Wood D2, before activation | 1 | ACAGCTCAAACA | CCGTAAAACGACGGCCAG | Fungi |
|  |  | 2 | GCGTTGCAAACT | CCGTAAAACGACGGCCAG |  |
| S8 | Wood D2, after activation | 1 | AGATGATCAGTC | CCGTAAAACGACGGCCAG | Fungi |
|  |  | 2 | AATGACCTCGTG | CCGTAAAACGACGGCCAG |  |
