# Supplementary material for: Metabolomic and high-throughput sequencing analysis—modern approach for the assessment of biodeterioration of materials from historic buildings
Source: Front Microbiol. 2015 Sep 29;6:979. doi: 10.3389/fmicb.2015.00979 (PMC4586457; doi:10.3389/fmicb.2015.00979)
Supplement: Supplementary file 3 [file Table3.DOCX]

**Table S3.** Archaeal/bacterial diversity in biodeteriorated wood and brick samples

| **Domain** | **Phylum** | **Genus** | **Abundance of archaeal/bacterial genera in samples [%]*** | | | | | |
| --- | --- | --- | --- | --- | --- | --- | --- | --- |
|  |  |  | **S1** | **S2** | **S3** | **S4** | **S5** | **S6** |
| *Archaea* | *Crenarchaeota* | *Nitrososphaera* | 0.20 | 0.30 | 0.00 | 0.90 | 0.00 | 0.00 |
| *Bacteria* | *Actinobacteria* | *Salinibacterium* | 0.15 | 0.15 | 0.50 | 0.30 | 0.30 | 1.00 |
|  |  | *Arthrobacter* | 5.60 | 6.50 | 1.00 | 4.70 | 0.80 | 0.80 |
|  |  | *Promicromonospora* | 0.80 | 0.75 | 0.70 | 0.80 | 11.30 | 2.20 |
|  |  | *Actinomycetospora* | 0.10 | 0.75 | 0.30 | 0.70 | 0.10 | 0.10 |
|  |  | *Pseudonocardia* | 2.65 | 4.10 | 0.45 | 2.00 | 0.40 | 0.50 |
|  |  | *Saccharopolyspora* | 0.00 | 0.70 | 0.05 | 0.10 | 0.00 | 0.10 |
|  |  | *Sporichthya* | 0.30 | 0.10 | 0.00 | 0.10 | 0.00 | 0.00 |
|  |  | *Streptomyces* | 0.00 | 0.10 | 0.00 | 0.00 | 0.10 | 0.70 |
|  |  | *Euzebya* | 0.25 | 0.25 | 0.35 | 1.00 | 0.25 | 0.50 |
|  |  | *Rubrobacter* | 0.10 | 0.30 | 0.10 | 0.40 | 0.10 | 0.20 |
|  | *Bacteroidetes* | *Hymenobacter* | 0.35 | 0.30 | 0.35 | 0.30 | 0.30 | 0.40 |
|  |  | *Porifericola* | 0.25 | 0.15 | 0.60 | 0.60 | 0.05 | 0.00 |
|  |  | *Rubricoccus* | 0.00 | 0.00 | 0.00 | 0.00 | 0.35 | 0.30 |
|  |  | *Balneola* | 0.30 | 0.65 | 2.45 | 0.80 | 1.35 | 0.30 |
|  | *Firmicutes* | *Bacillus* | 0.00 | 0.00 | 0.10 | 0.10 | 0.00 | 0.00 |
|  |  | *Marinibacillus* | 0.10 | 0.10 | 0.05 | 1.10 | 0.05 | 0.00 |
|  |  | *Paenibacillus* | 0.00 | 0.00 | 0.00 | 0.00 | 0.00 | 0.00 |
|  |  | *Sporosarcina* | 0.00 | 0.110 | 0.00 | 0.00 | 0.00 | 0.00 |
|  | *Nitrospirae* | *Nitrospira* | 0.20 | 0.15 | 0.00 | 0.20 | 0.00 | 0.00 |
|  | *Planctomycetes* | *Pirellula* | 0.35 | 0.05 | 0.00 | 0.50 | 0.05 | 0.00 |
|  |  | *Planctomyces* | 0.30 | 0.30 | 0.70 | 0.50 | 0.40 | 1.10 |
|  | *Alphaproteobacteria* | *Mycoplana* | 0.15 | 0.10 | 0.00 | 0.10 | 0.00 | 0.00 |
|  |  | *Devosia* | 1.20 | 1.00 | 1.05 | 1.40 | 5.60 | 3.70 |
|  |  | *Hyphomicrobium* | 1.80 | 0.25 | 0.10 | 0.20 | 0.10 | 0.10 |
|  |  | *Rhodoplanes* | 0.50 | 0.20 | 0.05 | 0.40 | 0.10 | 0.10 |
|  |  | *Chelativorans* | 3.90 | 1.80 | 1.10 | 5.00 | 1.40 | 0.50 |
|  |  | *Mesorhizobium* | 0.15 | 0.20 | 0.35 | 0.20 | 0.80 | 0.70 |
|  |  | *Amaricoccus* | 0.10 | 0.10 | 0.35 | 0.00 | 0.00 | 0.00 |
|  |  | *Marivita* | 1.15 | 0.85 | 1.15 | 0.90 | 0.85 | 1.00 |
|  |  | *Paracoccus* | 0.40 | 0.20 | 0.25 | 0.20 | 0.20 | 0.20 |
|  |  | *Roseomonas* | 0.45 | 0.30 | 0.30 | 0.40 | 0.30 | 0.30 |
|  |  | *Phaeospirillum* | 0.30 | 0.00 | 0.00 | 0.00 | 0.00 | 0.00 |
|  |  | *Erythrobacter* | 0.30 | 0.20 | 0.45 | 0.50 | 0.30 | 0.50 |
|  |  | *Kaistobacter* | 0.60 | 0.40 | 0.10 | 0.90 | 0.10 | 0.10 |
|  |  | *Sphingomonas* | 0.20 | 0.05 | 0.10 | 0.10 | 0.00 | 0.00 |
|  |  | *Sphingopyxis* | 0.70 | 0.05 | 0.15 | 0.10 | 0.10 | 0.10 |
|  | *Betaproteobacteria* | *Methylibium* | 3.20 | 0.55 | 0.30 | 1.00 | 0.25 | 0.60 |
|  |  | *Methylobacillus* | 0.10 | 0.00 | 0.00 | 0.10 | 0.55 | 0.10 |
|  | *Gammaproteobacteria* | *Marinimicrobium* | 0.00 | 0.00 | 0.65 | 0.00 | 0.00 | 0.00 |
|  |  | *Marinobacter* | 4.95 | 25.45 | 11.35 | 4.30 | 4.30 | 5.20 |
|  |  | *Microbulbifer* | 0.00 | 0.00 | 0.20 | 0.00 | 0.00 | 0.00 |
|  |  | *Shewanella* | 1.30 | 1.70 | 1.00 | 1.10 | 1.10 | 1.20 |
|  |  | *Halorhodospira* | 0.00 | 0.00 | 0.05 | 0.00 | 0.15 | 0.30 |
|  |  | *Alcanivorax* | 0.40 | 0.25 | 0.20 | 0.20 | 0.30 | 0.20 |
|  |  | *Halomonas* | 1.75 | 2.45 | 2.70 | 1.50 | 1.65 | 2.70 |
|  |  | *Saccharospirillum* | 0.00 | 0.25 | 0.35 | 0.00 | 0.00 | 0.00 |
|  |  | *Salinisphaera* | 0.00 | 0.00 | 0.00 | 0.00 | 0.00 | 0.10 |
|  |  | *Methylophaga* | 0.00 | 0.20 | 0.00 | 0.00 | 0.00 | 0.00 |
|  |  | *Panacagrimonas* | 0.30 | 0.00 | 0.00 | 0.00 | 0.00 | 0.00 |
|  |  | *Steroidobacter* | 0.80 | 0.20 | 0.05 | 0.10 | 0.20 | 0.50 |
|  |  | *Lysobacter* | 0.05 | 0.00 | 0.15 | 0.00 | 0.85 | 0.20 |
|  | *Verrucomicrobia* | *Pelagicoccus* | 0.00 | 0.00 | 0.10 | 0.10 | 0.50 | 0.20 |
|  | *Thermi* | *Deinococcus* | 0.80 | 0.60 | 0.60 | 0.60 | 0.45 | 0.60 |
|  |  | *Truepera* | 1.55 | 1.20 | 1.20 | 1.10 | 1.05 | 1.10 |

*percentage share of studied genera greater than 0.1%
