# Supplementary material for: Metabolomic and high-throughput sequencing analysis—modern approach for the assessment of biodeterioration of materials from historic buildings
Source: Front Microbiol. 2015 Sep 29;6:979. doi: 10.3389/fmicb.2015.00979 (PMC4586457; doi:10.3389/fmicb.2015.00979)
Supplement: Supplementary file 4 [file Table4.DOCX]

**Table S4.** Fungal diversity in biodeteriorated wood and brick samples

| **Phylum** | **Classes** | **Genus** | **Abundance of fungal genera in samples [%]*** | | | | | | | |
| --- | --- | --- | --- | --- | --- | --- | --- | --- | --- | --- |
|  |  |  | **S1** | **S2** | **S3** | **S4** | **S5** | **S6** | **S7** | **S8** |
|  |  | Other | 17.45 | 10.05 | 5.40 | 21.70 | 9.35 | 4.25 | 2.85 | 3.45 |
| *Ascomycota* | *Dothideomycetes* | *Cladosporium* | 2.55 | 5.55 | 1.55 | 3.20 | 0.30 | 0.25 | 0.60 | 0.45 |
|  |  | *Mycosphaerella* | 0.00 | 0.10 | 1.40 | 0.10 | 0.10 | 0.05 | 0.10 | 0.10 |
|  |  | *unidentified* | 0.60 | 1.40 | 0.80 | 20.90 | 0.60 | 0.70 | 1.00 | 1.10 |
|  |  | *Aureobasidium* | 0.00 | 0.35 | 0.00 | 0.00 | 0.00 | 0.00 | 0.05 | 0.00 |
|  |  | *Perisporiopsis* | 0.00 | 0.35 | 0.00 | 0.00 | 0.00 | 0.00 | 0.00 | 0.00 |
|  |  | *Pseudeurotium* | 0.00 | 0.00 | 0.00 | 0.10 | 0.00 | 0.00 | 0.00 | 0.00 |
|  |  | *Xenobotryosphaeria* | 0.00 | 0.15 | 0.00 | 0.00 | 0.00 | 0.00 | 0.00 | 0.00 |
|  |  | *Setoseptoria* | 0.00 | 1.30 | 0.00 | 0.00 | 0.00 | 0.00 | 0.00 | 0.00 |
|  |  | *Stagonospora* | 0.10 | 0.00 | 0.00 | 0.00 | 0.00 | 0.00 | 0.00 | 0.00 |
|  |  | *Alternaria* | 8.30 | 4.25 | 2.10 | 0.95 | 0.30 | 0.25 | 0.40 | 0.50 |
|  |  | *Cochliobolus* | 0.10 | 0.00 | 0.00 | 0.00 | 0.00 | 0.00 | 0.00 | 0.00 |
|  | *Eurotiomycetes* | *Aspergillus* | 0.00 | 0.00 | 0.10 | 0.00 | 0.00 | 0.00 | 0.00 | 0.00 |
|  |  | *Emericella* | 0.80 | 2.25 | 0.55 | 1.60 | 0.10 | 0.10 | 1.05 | 0.20 |
|  |  | *Penicillium* | 0.15 | 0.35 | 0.30 | 0.20 | 0.25 | 0.25 | 1.65 | 12.00 |
|  |  | *Verrucaria* | 0.35 | 0.00 | 0.00 | 0.65 | 0.00 | 0.05 | 0.00 | 0.00 |
|  |  | *unidentified* | 0.15 | 0.00 | 0.00 | 0.20 | 0.00 | 0.00 | 0.45 | 0.00 |
|  | *Leotiomycetes* | *Hymenoscyphus* | 0.30 | 0.00 | 0.00 | 0.00 | 0.00 | 0.00 | 0.00 | 0.00 |
|  |  | *Geomyces* | 0.05 | 0.50 | 0.10 | 0.00 | 0.25 | 0.10 | 0.00 | 0.05 |
|  | *Saccharomycetes* | *Candida* | 0.00 | 0.00 | 0.00 | 0.45 | 0.00 | 0.00 | 0.00 | 0.00 |
|  | *Sordariomycetes* | *Latruncellus* | 0.00 | 0.00 | 0.10 | 0.00 | 0.00 | 0.00 | 0.00 | 0.00 |
|  |  | *Valsa* | 0.00 | 0.00 | 0.00 | 0.00 | 0.00 | 0.00 | 0.10 | 0.00 |
|  |  | *Pochonia* | 0.00 | 0.20 | 0.00 | 0.00 | 0.00 | 0.00 | 0.00 | 0.00 |
|  |  | *Engyodontium* | 0.75 | 9.25 | 0.60 | 0.75 | 0.30 | 0.25 | 0.45 | 0.40 |
|  |  | *Hypomyces* | 0.00 | 0.30 | 0.00 | 0.00 | 0.00 | 0.00 | 0.00 | 0.00 |
|  |  | *Acremonium* | 0.10 | 0.50 | 0.10 | 0.45 | 0.00 | 0.00 | 0.05 | 0.05 |
|  |  | *Stilbella* | 0.00 | 0.30 | 0.00 | 0.00 | 0.00 | 0.00 | 0.00 | 0.00 |
|  |  | *unidentified* | 0.30 | 16.00 | 0.40 | 0.65 | 0.40 | 0.45 | 0.65 | 0.70 |
|  |  | *Scedosporium* | 0.00 | 0.00 | 0.00 | 0.00 | 0.10 | 0.00 | 0.00 | 0.00 |
|  |  | *Khuskia* | 0.00 | 0.00 | 0.10 | 0.00 | 0.00 | 0.00 | 0.00 | 0.00 |
|  |  | *unidentified* | 6.25 | 14.80 | 5.00 | 4.65 | 68.80 | 80.60 | 7.30 | 7.60 |
|  | *unidentified* | *unidentified* | 2.45 | 4.30 | 3.40 | 3.95 | 3.10 | 6.65 | 73.95 | 65.55 |
| *Basidiomycota* | *Agaricomycetes* | *Coprinus* | 0.20 | 0.00 | 0.00 | 0.00 | 0.00 | 0.00 | 0.00 | 0.00 |
|  |  | *Cortinarius* | 0.20 | 0.00 | 0.00 | 0.00 | 0.00 | 0.00 | 0.00 | 0.00 |
|  |  | *Entoloma* | 0.10 | 0.00 | 0.00 | 0.00 | 0.00 | 0.00 | 0.00 | 0.00 |
|  |  | *Laccaria* | 0.10 | 0.00 | 0.00 | 0.00 | 0.00 | 0.00 | 0.00 | 0.00 |
|  |  | *Crepidotus* | 0.00 | 0.00 | 0.00 | 0.00 | 0.00 | 0.00 | 0.15 | 0.00 |
|  |  | *Marasmius* | 0.10 | 0.00 | 0.00 | 0.00 | 0.00 | 0.00 | 0.00 | 0.00 |
|  |  | *Panellus* | 0.10 | 0.00 | 0.00 | 0.00 | 0.00 | 0.00 | 0.00 | 0.00 |
|  |  | *Volvariella* | 4.20 | 0.25 | 3.00 | 0.20 | 0.20 | 0.15 | 0.25 | 0.20 |
|  |  | *Coprinellus* | 0.20 | 0.00 | 0.05 | 0.10 | 0.00 | 0.00 | 0.15 | 0.00 |
|  |  | *Psathyrella* | 0.10 | 0.00 | 0.00 | 0.00 | 0.00 | 0.00 | 0.00 | 0.00 |
|  |  | *Hypholoma* | 0.10 | 0.00 | 0.00 | 0.00 | 0.00 | 0.00 | 0.00 | 0.00 |
|  |  | *Pholiota* | 0.10 | 0.00 | 0.00 | 0.00 | 0.00 | 0.00 | 0.00 | 0.00 |
|  |  | *Psilocybe* | 0.00 | 0.00 | 0.00 | 0.35 | 0.00 | 0.00 | 0.00 | 0.00 |
|  |  | *Stropharia* | 0.10 | 0.00 | 0.00 | 0.10 | 0.00 | 0.00 | 0.00 | 0.00 |
|  |  | *unidentified* | 0.05 | 0.00 | 0.00 | 0.10 | 0.00 | 0.00 | 0.00 | 0.00 |
|  |  | *Clitocybe* | 0.00 | 0.25 | 0.00 | 0.00 | 0.00 | 0.00 | 0.00 | 0.00 |
|  |  | *unidentified* | 37.95 | 1.05 | 0.95 | 0.80 | 0.60 | 0.60 | 1.05 | 1.10 |
|  |  | *Vuilleminia* | 0.00 | 0.00 | 0.15 | 0.00 | 0.00 | 0.00 | 0.00 | 0.00 |
|  |  | *Waitea* | 0.10 | 0.00 | 0.00 | 0.00 | 0.00 | 0.00 | 0.00 | 0.00 |
|  |  | *Mensularia* | 0.00 | 0.00 | 0.00 | 0.15 | 0.00 | 0.00 | 0.00 | 0.00 |
|  |  | *Hyphodontia* | 0.10 | 0.00 | 0.00 | 0.10 | 0.00 | 0.00 | 0.00 | 0.00 |
|  |  | *unidentified* | 0.55 | 0.00 | 0.10 | 0.45 | 0.00 | 0.00 | 0.00 | 0.00 |
|  |  | *Meripilus* | 0.05 | 0.00 | 0.00 | 0.00 | 0.00 | 0.00 | 0.00 | 0.00 |
|  |  | *Physisporinus* | 0.10 | 0.00 | 0.00 | 0.00 | 0.00 | 0.00 | 0.00 | 0.00 |
|  |  | *Bjerkandera* | 0.20 | 0.00 | 0.00 | 0.00 | 0.00 | 0.00 | 0.00 | 0.00 |
|  |  | *Heterobasidion* | 0.60 | 0.00 | 0.00 | 0.00 | 0.00 | 0.00 | 0.00 | 0.00 |
|  |  | *Peniophora* | 0.00 | 0.00 | 0.00 | 0.00 | 0.00 | 0.00 | 0.40 | 0.00 |
|  |  | *unidentified* | 0.00 | 2.35 | 0.00 | 0.05 | 0.00 | 0.00 | 0.00 | 0.10 |
|  |  | *Rhodotorula* | 0.00 | 0.00 | 0.20 | 0.00 | 0.00 | 0.00 | 0.00 | 0.00 |
|  | *Tremellomycetes* | *Mrakia* | 0.10 | 0.00 | 0.00 | 0.00 | 0.00 | 0.00 | 0.00 | 0.00 |
|  |  | *Udeniomyces* | 0.10 | 0.00 | 0.00 | 0.00 | 0.00 | 0.00 | 0.00 | 0.00 |
|  |  | *Cryptococcus* | 0.10 | 0.00 | 0.00 | 0.00 | 0.00 | 0.00 | 0.00 | 0.00 |
| *Glomeromycota* | *unidentified* | *unidentified* | 0.30 | 0.55 | 15.55 | 0.40 | 0.30 | 0.35 | 0.45 | 0.65 |
| *Zygomycota* | *Incertae sedis* | *Ramicandelaber* | 0.75 | 0.40 | 1.50 | 22.75 | 0.30 | 0.40 | 0.50 | 0.70 |
|  |  | *Mortierella* | 0.00 | 0.00 | 1.60 | 0.00 | 0.00 | 0.00 | 0.05 | 0.05 |
|  |  | *Rhizopus* | 0.00 | 0.00 | 0.00 | 0.00 | 0.00 | 0.00 | 0.00 | 0.00 |
|  |  | *unidentified* | 7.60 | 17.90 | 52.20 | 11.95 | 12.50 | 3.70 | 4.60 | 3.50 |

*percentage share of studied genera greater than 0.1%
