# Supplementary material for: Metabolomic and high-throughput sequencing analysis—modern approach for the assessment of biodeterioration of materials from historic buildings
Source: Front Microbiol. 2015 Sep 29;6:979. doi: 10.3389/fmicb.2015.00979 (PMC4586457; doi:10.3389/fmicb.2015.00979)
Supplement: Supplementary file 5 [file Table5.DOCX]

**Table S5.** Metabolic pathways detected in brick and wood samples

| **No** | **Metabolic pathways** | **Brick B124** | **Wood B124** |
| --- | --- | --- | --- |
| 1 | Alanine, aspartate, glutamate metabolism | + | + |
| 2 | Alpha-linolenic acid metabolism | + | + |
| 3 | Arachidonic acid metabolism | - | + |
| 4 | Arginine and proline metabolism | + | + |
| 5 | Atrazine degradation | - | + |
| 6 | Benzoate degradation | + | + |
| 7 | Benzoxazinone degradation | + | + |
| 8 | Beta-Alanine metabolism | + | - |
| 9 | Biosynthesis of type II polyketide products | + | + |
| 10 | Biosynthesis of unsaturated fatty acids | - | + |
| 11 | Biosynthesis of other secondary metabolites | + | + |
| 12 | Butonate metabolism | - | + |
| 13 | Carbon fixation | + | - |
| 14 | Carotenoid biosynthesis | - | + |
| 15 | Citrate cycle (TCA cycle) | + | + |
| 16 | Cystein and methionine metabolism | + | + |
| 17 | D-Arginine and O-ornithine metabolism | + | + |
| 18 | Diterpenoid biosynthesis | + | + |
| 19 | Ethylbenzene degradation | + | - |
| 20 | Fatty acid biosynthesis | - | + |
| 21 | Fatty acid elongation in mitochondria | - | + |
| 22 | Flavone and falvonol biosynthesis | + | - |
| 23 | Flavonoid biosynthesis | + | + |
| 24 | Fluorobenzoate degradation | + | + |
| 25 | Folate metabolism | - | + |
| 26 | Glutathione metabolism | - | + |
| 27 | Glucosinolate biosynthesis | + | - |
| 28 | Glycerophospholipid metabolism | + | + |
| 29 | Glycine, serine and threonine metabolism | - | + |
| 30 | Glycolysis/ Gluconeogenesis | + | + |
| 31 | Glycosaminoglycan biosynthesis | + | + |
| 32 | Glycosphingolipids biosynthesis | + | + |
| 33 | Glycosyltransferases metabolism | + | - |
| 34 | Histidine metabolism | + | + |
| 35 | Isoquinoline alkaloid biosynthesis | + | + |
| 36 | Linoleic acid metabolism | - | + |
| 37 | Lipoic acid metabolism | + | - |
| 38 | Lysine biosynthesis | + | + |
| 39 | Metabolism of other amino acids | - | + |
| 40 | Methane metabolism | - | + |
| 41 | Monoterpenoid biosynthesis | - | + |
| 42 | Naphtalene degradation | + | + |
| 43 | Nicotinate and nicotinamide metabolism | + | - |
| 44 | Nitrogen metabolism | - | + |
| 45 | Nucleotide metabolism | + | + |
| 46 | Pentose phosphate metabolism | + | - |
| 47 | Phenyloalanine metabolism | + | - |
| 48 | Phenyloalanine, tyrosine, tryptophan biosynthesis | + | + |
| 49 | Phenylpropanoid biosynthesis | + | + |
| 50 | Phosphonate and phosphinate metabolism | - | + |
| 51 | Polyketides and nonribosomal peptides biosynthesis | + | + |
| 52 | Porphyrin and chlorophyll metabolism | + | - |
| 53 | Proteoglycans metabolism | + | + |
| 54 | Purine metabolism | + | - |
| 55 | Retinol metabolism | - | + |
| 56 | Sesquiterpenoid biosynthesis | + | + |
| 57 | Sphingolipid metabolism | - | + |
| 58 | Starch and sucrose metabolism | - | + |
| 59 | Steroid biosynthesis | + | + |
| 60 | Streptomycin biosynthesis | - | + |
| 61 | Sulphur metabolism | - | + |
| 62 | Synthesis and degradation of ketone bodies | - | + |
| 63 | Taurine and hypotaurine metabolism | + | + |
| 64 | Terpenoid backbone biosynthesis | - | + |
| 65 | Thiamine metabolism | - | + |
| 66 | Tropane, piperidine and pyridine alkaloid biosynthesis | + | - |
| 67 | Tryptophan metabolism | + | + |

(+) metabolic pathway present in sample; (-) metabolic pathway absent in sample

■ pathways typical for active primary metabolism of aminoacids, vitamins, glycerophospholipids, sulphur and nitrogen; ■ pathways typical for active primary metabolism of organic acids including fatty acids and steroids; ■ secondary metabolism pathways; ■ pathways typical for active primary metabolism of dyes and aromatic compounds; ■ pathways typical for active primary metabolism of phototrophs (CO_2_ assimilation, chlorophyll degradation); ■ pathways involved in degradation of compounds which may originate from herbicides, insecticides, biocides and other compounds used to preserve historic materials
