# Supplementary material for: Metabolomic and high-throughput sequencing analysis—modern approach for the assessment of biodeterioration of materials from historic buildings
Source: Front Microbiol. 2015 Sep 29;6:979. doi: 10.3389/fmicb.2015.00979 (PMC4586457; doi:10.3389/fmicb.2015.00979)
Supplement: Supplementary file 6 [file Table6.DOCX]

**Table S6**. List of putatively identified metabolites in brick samples, detected in reverse phase and ZIC-HILIC chromatography.

| **Retention time (minutes)** | **Predicted RT (minutes)** | **m/z** | **mass error (ppm)** | **Formula** | **Putatively identified metabolite** | **Samples** |
| --- | --- | --- | --- | --- | --- | --- |
|  |  |  |  |  |  |  |
| **Negative ion mode - Reverse phase column** | | | | | | |
| 0.16 | N/A | 116.9282 | -0.77 | CrH2O4 | chromate | S1,S6,S8,S9 |
| 0.30 | N/A | 61.9882 | 1.52 | HNO3 | Nitrate | S2,S5,S6,S7,S8,S1,S9 |
| 0.49 | N/A | 170.8860 | 4.93 | C2H2BrClO2 | 2-Bromo-2-chloroacetic acid | S2,S5,S6,S7,S8,S1,S9 |
| 0.55 | N/A | 96.9598 | -0.41 | H2O4S | Sulfate | S2,S5,S6,S7,S8,S1,S9 |
| 0.62 | N/A | 119.0360 | -0.79 | C5H4N4 | Purine | S2,S5,S6,S7,S8,S1,S9 |
| 0.64 | N/A | 249.0588 | 1.37 | C25H24O11 | Epigallocatechin 5,3',5'-trimethyl ether 3-O-gallate | S1,S6,S8 |
| 0.68 | N/A | 301.0035 | -0.99 | C13H12Cl2O4 | ethacrynate | S1,S6,S8 |
| 0.68 | N/A | 124.9913 | 1.02 | C2H6O4S | 2-Hydroxyethanesulfonate | S2,S5,S6,S7,S8,S1,S9 |
| 0.71 | N/A | 297.1527 | 3.37 | C28H40N10O5 | Arg-Trp-Val-His | S1,S9 |
| 0.71 | N/A | 128.0353 | 1.70 | C5H7NO3 | L-1-Pyrroline-3-hydroxy-5-carboxylate | S2,S5,S6,S7,S8,S1,S9 |
| 0.72 | N/A | 265.1479 | 0.80 | C12H26O4S | sodium dodecyl sulfate | S1,S9 |
| 0.79 | N/A | 153.0223 | -1.23 | C4H10O4S | Diethyl sulfate | S2,S5,S6,S7,S8,S1 |
| 0.82 | N/A | 288.9973 | 3.16 | C6H11O11P | 3-phosphoglucarate | S1,S6,S8 |
| 0.82 | N/A | 311.1684 | 1.39 | C33H52O11 | Spongipregnoloside A | S1,S9 |
| 0.83 | N/A | 433.1275 | -3.59 | C25H22O7 | Artobiloxanthone | S1,S9 |
| 0.86 | N/A | 263.0750 | 3.28 | C27H28O11 | Tremulacin | S1,S6,S8 |
| 0.86 | N/A | 447.1431 | -3.62 | C26H24O7 | Cycloartomunin | S1,S6,S8,S9 |
| 0.90 | N/A | 159.0662 | 0.74 | C7H12O4 | [FA (7:0/2:0)] Heptanedioic acid | S2,S5,S6,S7,S8,S9 |
| 0.94 | N/A | 225.1126 | -1.99 | C12H18O4 | [FA oxo,hydroxy(5:1/5:0)] (1S,2R)-3-oxo-2-(5'-hydroxy-2'Z-pentenyl)-cyclopentaneacetic acid | S2,S5,S6,S7,S8,S1 |
| 1.00 | N/A | 237.1131 | 0.55 | C26H36O8 | all-trans-Retinoyl-beta-glucuronide | S2,S5,S6,S7,S8,S1 |
| 1.01 | N/A | 171.0659 | -0.69 | C8H12O4 | [FA dioxo(8:0)] 4,7-dioxo-octanoic acid | S2,S5,S6,S7,S8,S1,S9 |
| 1.10 | N/A | 178.0506 | -0.66 | C9H9NO3 | Hippurate | S2,S5,S6,S7,S8 |
| 1.12 | N/A | 150.0191 | -2.31 | C7H5NO3 | 6-Imino-5-oxocyclohexa-1,3-dienecarboxylate | S2,S5,S6,S7,S8 |
| 1.12 | N/A | 145.0611 | -3.94 | C5H10N2O3 | L-Glutamine | S2,S5,S6,S7,S8,S1 |
| 1.15 | N/A | 118.9416 | -3.43 | H3O9P3 | Trimetaphosphate | S1,S6,S8,S9 |
| 1.15 | N/A | 170.0819 | -0.54 | C8H13NO3 | N-Butyryl-L-homoserine lactone | S2,S5,S6,S7,S8 |
| 1.15 | N/A | 263.1030 | -2.08 | C13H16N2O4 | alpha-N-Phenylacetyl-L-glutamine | S2,S5,S6,S7,S8,S1 |
| 1.20 | N/A | 144.0452 | -0.27 | C9H7NO | 3-Methyleneoxindole | S2,S5,S6,S7,S8 |
| 1.22 | N/A | 182.0094 | 0.98 | C7H5NO5 | chelidamate | S2,S5,S6,S7,S8,S1,S9 |
| 1.30 | N/A | 116.9282 | -0.67 | CrH2O4 | chromate | S2,S5,S6,S7,S8,S1,S9 |
| 1.32 | N/A | 222.0160 | -1.09 | C8H15O2Se | 8-seleno-octanoate | S2,S5,S6,S7,S8 |
| 1.35 | N/A | 226.0352 | -1.13 | C9H9NO6 | 5-(2'-Carboxyethyl)-4,6-dihydroxypicolinate | S2,S5,S6,S7,S8,S1 |
| 1.36 | N/A | 196.0251 | 0.93 | C8H7NO5 | 3-Hydroxy-2-methylpyridine-4,5-dicarboxylate | S2,S5,S6,S7,S8,S1,S9 |
| 1.46 | N/A | 138.0194 | -0.45 | C6H5NO3 | 6-Hydroxynicotinate | S2,S5,S6,S7,S8,S1,S9 |
| 1.52 | N/A | 608.2772 | 1.41 | C36H39N3O6 | Niguldipine | S2,S5,S6,S7,S8 |
| 1.54 | N/A | 121.0291 | -1.20 | C7H6O2 | Benzoate | S2,S5,S6,S7,S8,S1,S9 |
| 1.57 | N/A | 183.0052 | 3.44 | C6H4N2O5 | 2,4-Dinitrophenol | S2,S5,S6,S7,S8,S1,S9 |
| 1.60 | N/A | 188.0348 | -1.46 | C10H7NO3 | Kynurenate | S2,S5,S6,S7,S8,S1 |
| 1.68 | N/A | 152.0352 | 0.47 | C7H7NO3 | 3-Hydroxyanthranilate | S2,S5,S6,S7,S8,S1,S9 |
| 1.72 | N/A | 166.0144 | 0.05 | C7H5NO4 | Pyridine-2,3-dicarboxylate | S2,S5,S6,S7,S8,S1,S9 |
| 1.76 | N/A | 111.0083 | -2.29 | C5H4O3 | 2-Furoate | S2,S5,S6,S7,S8,S9 |
| 1.80 | N/A | 226.9933 | -4.53 | C7H4N2O7 | 3,5-Dinitrosalicylic acid | S2,S5,S6,S7,S8,S1,S9 |
| 1.84 | N/A | 225.1608 | 0.61 | C12H22N2O2 | 1,8-Diazacyclotetradecane-2,9-dione | S1,S6,S8 |
| 1.93 | N/A | 271.0220 | -0.84 | C7H13O9P | &alpha;-(2,6-anhydro-3-deoxy-D-arabino-heptulopyranosid)onate 7-phosphate | S2,S5,S6,S7,S8 |
| 1.94 | N/A | 208.0252 | 1.54 | C18H14N2O10 | Muscapurpurin | S2,S5,S6,S7,S8 |
| 1.97 | N/A | 267.0625 | 1.64 | C11H12N2O6 | Portulacaxanthin III | S2,S5,S6,S7,S8 |
| 2.18 | N/A | 210.0407 | 0.71 | C9H9NO5 | 5-(2'-Formylethyl)-4,6-dihydroxypicolinate | S2,S5,S6,S7,S8,S1,S9 |
| 2.72 | N/A | 304.0205 | -1.39 | C12H7N3O7 | 2,4-Dinitro-1-(3-nitrophenoxy)benzene | S2,S5,S6,S7,S8 |
| 3.05 | N/A | 138.0193 | -0.65 | C6H5NO3 | 6-Hydroxynicotinate | S2,S5,S6,S7,S8,S1,S9 |
| 3.09 | N/A | 247.0359 | 0.47 | C11H8N2O5 | (E)-2-(2-Furyl)-3-(5-nitro-2-furyl)acrylamide | S2,S5,S6,S7,S8 |
| 4.60 | N/A | 549.1358 | 3.00 | C21H26N8O6S2 | Cefclidin | S2,S5,S6,S7,S8,S1 |
| 4.70 | N/A | 267.0615 | -1.96 | C11H12N2O6 | Portulacaxanthin III | S2,S5,S6,S7,S8 |
| 4.91 | N/A | 189.0304 | 0.25 | C9H6N2O3 | 4-Nitroquinoline N-oxide | S2,S5,S6,S7,S8 |
| 5.09 | N/A | 380.0158 | 1.92 | C8H17NO12P2 | N-Acetyl-D-glucosamine 1,6-bisphosphate | S2,S5,S6,S7,S8 |
| 5.67 | N/A | 265.1478 | 0.45 | C12H26O4S | sodium dodecyl sulfate | S2,S5,S6,S7,S8,S1,S9 |
| 5.78 | N/A | 297.1529 | 4.03 | C28H40N10O5 | Arg-Trp-Val-His | S2,S5,S6,S7,S8,S1,S9 |
| 5.78 | N/A | 413.1597 | -1.71 | C23H26O7 | [Fv Trihydroxy,methoxy,hydroxy,ethyl(9:1)] 5,7,4'-Trihydroxy-3'-methoxy-6-(beta-hydroxyethyl)-8-prenylflavanone | S2,S5,S6,S7,S8,S1 |
| 5.80 | N/A | 433.1274 | -3.84 | C25H22O7 | Artobiloxanthone | S2,S5,S6,S7,S8,S1,S9 |
| 5.80 | N/A | 549.1358 | 3.01 | C21H26N8O6S2 | Cefclidin | S2,S5,S6,S7,S8,S1 |
| 6.07 | N/A | 427.1758 | -0.57 | C24H28O7 | [Fv] Heteroflavanone B | S1,S6,S8 |
| 6.44 | N/A | 311.1689 | 2.97 | C33H52O11 | Spongipregnoloside A | S2,S5,S6,S7,S8,S1,S9 |
| 6.52 | N/A | 447.1439 | -1.83 | C26H24O7 | Cycloartomunin | S2,S5,S6,S7,S8,S1,S9 |
| 6.68 | N/A | 396.1460 | 2.46 | C22H23NO6 | Aureothin | S2,S5,S6,S7,S8,S1,S9 |
| 7.29 | N/A | 293.1793 | -1.86 | C29H48N8O5 | Lys-Lys-Lys-Trp | S2,S5,S6,S7,S8,S1,S9 |
| 7.35 | N/A | 461.1596 | -1.60 | C27H26O7 | Euchrenone b3 | S2,S5,S6,S7,S8,S1,S9 |
| 7.46 | N/A | 146.0241 | -3.23 | C8H5NO2 | Indole-5,6-quinone | S2,S5,S6,S7,S8,S1 |
| 7.60 | N/A | 441.1909 | -1.62 | C25H30O7 | [Fv] Exiguaflavanone M | S2,S5,S6,S7,S8,S1 |
| 7.77 | N/A | 242.1765 | 2.29 | C13H25NO3 | N-Undecanoylglycine | S2,S5,S6,S7,S8,S1,S9 |
| 8.25 | N/A | 475.1755 | -2.43 | C21H28N6O5S | Ala-Phe-Cys-His | S2,S5,S6,S7,S8,S1,S9 |
| 8.40 | N/A | 143.1077 | 1.06 | C8H16O2 | [FA (8:0)] octanoic acid | S1,S6,S8,S9 |
| 8.41 | N/A | 116.9282 | -0.63 | CrH2O4 | chromate | S2,S5,S6,S7,S8,S1,S9 |
| 8.42 | N/A | 119.0360 | -0.72 | C5H4N4 | Purine | S2,S5,S6,S7,S8,S1,S9 |
| 8.67 | N/A | 455.2062 | 3.42 | C22H28N6O5 | His-Phe-Gly-Pro | S2,S5,S6,S7,S8,S1 |
| 8.87 | N/A | 387.0836 | 1.08 | C18H16N2O8 | dopaxanthin quinone | S2,S5,S6,S7,S8,S1 |
| 10.45 | N/A | 239.0672 | 0.16 | C10H12N2O5 | Dinoseb | S1,S6,S8,S9 |
| 10.47 | N/A | 157.1232 | 0.01 | C9H18O2 | Nonanoic acid | S2,S5,S6,S7,S8,S1,S9 |
| 10.56 | N/A | 247.0730 | 3.07 | C12H12N2O4 | 5-Hydroxyindoleacetylglycine | S2,S5,S6,S7,S8 |
| 10.86 | N/A | 171.1388 | -0.36 | C10H20O2 | Decanoic acid | S2,S5,S6,S7,S8,S1,S9 |
| 11.28 | N/A | 303.0377 | -2.65 | C10H13N2O7P | thymidine 3'5'-cyclic monophosphate | S2,S5,S6,S7,S8,S1 |
| 12.24 | N/A | 171.1388 | -0.25 | C10H20O2 | Decanoic acid | S1,S6,S8,S9 |
| 12.53 | N/A | 270.0520 | -4.33 | C30H22O10 | Isochamaejasmin | S2,S5,S6,S7,S8,S1 |
| 13.81 | N/A | 185.1544 | -0.17 | C11H22O2 | [FA (11:0)] undecanoic acid | S9 |
| 14.75 | N/A | 165.0403 | 0.56 | C5H10O6 | L-Arabinonate | S2,S5,S6,S7,S8,S1,S9 |
| 15.26 | N/A | 199.1703 | 0.91 | C12H24O2 | Dodecanoic acid | S1,S6,S8,S9 |
| 16.63 | N/A | 213.1858 | -0.06 | C13H26O2 | CAI-1 | S2,S5,S6,S7,S8,S1,S9 |
| 16.64 | N/A | 271.2277 | 0.23 | C16H32O3 | 16-hydroxypalmitate | S2,S5,S6,S7,S8,S1,S9 |
| 17.10 | N/A | 509.1318 | 3.77 | C23H26O13 | Quercetin 3,3'-dimethyl ether 4'-glucoside | S2,S5,S6,S7,S8 |
| 17.26 | N/A | 239.2009 | -2.12 | C15H28O2 | [FA dimethyl(13:0)] 2,5-dimethyl-2E-tridecenoic acid | S2,S5,S6,S7,S8,S1,S9 |
| 17.66 | N/A | 552.3302 | -0.44 | C26H52NO9P | PS(20:0/0:0) | S2,S5,S6,S7,S8 |
| 17.93 | N/A | 227.2018 | 1.78 | C14H28O2 | Tetradecanoic acid | S1,S6,S8,S9 |
| 18.43 | N/A | 253.2174 | 1.12 | C16H30O2 | (9Z)-Hexadecenoic acid | S1,S6,S8,S9 |
| 18.84 | N/A | 279.2328 | 0.27 | C18H32O2 | Linoleate | S2,S5,S6,S7,S8,S1,S9 |
| 19.12 | N/A | 241.2175 | 1.87 | C15H30O2 | [FA methyl(14:0)] 12-methyl-tetradecanoic acid | S1,S6,S8,S9 |
| 19.30 | N/A | 382.2351 | -2.85 | C17H38NO6P | [PC methyl(8:2)] 1-octyl-2-methyl-sn-glycero-3-phosphocholine | S2,S5,S6,S7,S8 |
| 19.56 | N/A | 365.2457 | -4.09 | C43H73O7P | PA(P-18:0/22:6(4Z,7Z,10Z,13Z,16Z,19Z)) | S2,S5,S6,S7,S8,S1,S9 |
| 19.59 | N/A | 496.2285 | 2.80 | C19H31N9O7 | Ala-Asp-Arg-His | S2,S5,S6,S7,S8,S1,S9 |
| 19.64 | N/A | 267.2327 | -0.26 | C17H32O2 | omega-Cyclohexylundecanoic acid | S2,S5,S6,S7,S8,S1,S9 |
| 19.78 | N/A | 370.2442 | -4.22 | C17H33N5O4 | Lys-Lys-Pro | S2,S5,S6,S7,S8,S1,S9 |
| 20.17 | N/A | 367.2442 | -1.11 | C42H73O8P | PA(17:1(9Z)/22:4(7Z,10Z,13Z,16Z)) | S2,S5,S6,S7,S8,S1,S9 |
| 20.23 | N/A | 441.2042 | 1.33 | C17H30N8O4S | Met-Arg-His | S1,S6,S8,S9 |
| 20.33 | N/A | 499.2644 | 2.48 | C20H36N8O7 | Arg-Thr-Gln-Pro | S2,S5,S6,S7,S8,S1 |
| 20.34 | N/A | 255.2333 | 2.25 | C16H32O2 | Hexadecanoic acid | S1,S6,S8,S9 |
| 20.34 | N/A | 391.2091 | -1.64 | C18H28N6O4 | Phe-Ala-Arg | S1,S6,S8,S9 |
| 20.35 | N/A | 425.1886 | -3.27 | C16H26N8O6 | Asp-Arg-His | S2,S5,S6,S7,S8,S1,S9 |
| 20.36 | N/A | 448.2666 | -2.11 | C21H35N7O4 | Lys-Phe-Arg | S1,S6,S8,S9 |
| 20.44 | N/A | 281.2489 | 1.78 | C18H34O2 | [FA (18:1)] 9Z-octadecenoic acid | S1,S6,S8,S9 |
| 21.30 | N/A | 269.2487 | 1.12 | C17H34O2 | [FA (17:0)] heptadecanoic acid | S2,S5,S6,S7,S8,S1,S9 |
| 21.79 | N/A | 295.2634 | -2.27 | C19H36O2 | [FA methyl(18:0)] 11R,12S-methylene-octadecanoic acid | S2,S5,S6,S7,S8,S1,S9 |
| 22.01 | N/A | 393.2781 | -3.92 | C27H38O2 | [ST (4:0/2:0)] (5Z,7E)-(3S)-9,10-seco-5,7,10(19),16-cholestatetraen-23-yne-3,25-diol | S2,S5,S6,S7,S8,S1,S9 |
| 22.47 | N/A | 213.0151 | 2.67 | C5H14N2O2Se | selenalysine | S2,S5,S6,S7,S8,S1,S9 |
| 22.48 | N/A | 283.2647 | 2.29 | C18H36O2 | Octadecanoic acid | S1,S6,S8,S9 |
| 22.48 | N/A | 426.2002 | 2.38 | C18H29N5O7 | Asn-Thr-Pro-Pro | S2,S5,S6,S7,S8,S1,S9 |
| 22.49 | N/A | 419.2409 | -0.33 | C20H32N6O4 | Phe-Val-Arg | S2,S5,S6,S7,S8,S1,S9 |
| 22.51 | N/A | 538.1984 | 1.76 | C23H33N5O8S | Asp-Met-Phe-Gln | S2,S5,S6,S7,S8,S1,S9 |
| 24.09 | N/A | 61.9882 | 1.52 | HNO3 | Nitrate | S2,S5,S6,S7,S8,S1,S9 |
| 24.32 | N/A | 311.2960 | 2.30 | C20H40O2 | [FA (20:0)] eicosanoic acid | S2,S5,S6,S7,S8,S1,S9 |
| 24.54 | N/A | 116.9287 | 3.12 | CrH2O4 | chromate | S2,S5,S6,S7,S8,S1,S9 |
| 25.36 | N/A | 119.0361 | -0.38 | C5H4N4 | Purine | S2,S5,S6,S7,S8,S1,S9 |
| 25.36 | N/A | 61.9882 | 1.69 | HNO3 | Nitrate | S1,S6,S8,S9 |
| 25.88 | N/A | 339.3272 | 1.71 | C22H44O2 | Docosanoic acid | S1,S6,S8,S9 |
| 26.30 | N/A | 541.3072 | 0.39 | C34H42N2O4 | Belladonnine | S2,S5,S6,S7,S8 |
| 26.68 | N/A | 353.3418 | -1.43 | C23H46O2 | [FA (23:0)] tricosanoic acid | S2,S5,S6,S7,S8,S1,S9 |
| 27.58 | N/A | 367.3587 | 1.99 | C24H48O2 | Tetracosanoic acid | S2,S5,S6,S7,S8,S1,S9 |
| 27.81 | N/A | 535.3771 | -3.56 | C35H52O4 | Hyperforin | S2,S5,S6,S7,S8 |
| 28.49 | N/A | 61.9882 | 1.42 | HNO3 | Nitrate | S1,S6,S8,S9 |
| 31.86 | N/A | 61.9882 | 1.30 | HNO3 | Nitrate | S1,S6,S8,S9 |
| 35.89 | N/A | 815.7459 | -4.49 | C53H100O5 | estolide | S2,S5,S6,S7,S8,S1,S9 |
| 35.90 | N/A | 962.7600 | 1.92 | C57H106NO8P | [PE (26:2/26:2)] 1,2-di-(5Z,9Z-hexacosadienoyl)-sn-glycero-3-phosphoethanolamine | S2,S5,S6,S7,S8,S1,S9 |
| 35.92 | N/A | 813.2810 | -1.25 | C37H50O20 | Jionoside B1 | S2,S5,S6,S7,S8,S1,S9 |
| 35.94 | N/A | 1064.7220 | 4.69 | C59H104NO13P | PE(16:0/18:1(9Z))-15-isoLG hydroxylactam | S2,S5,S6,S7,S8,S1,S9 |
| 35.94 | N/A | 61.9882 | 1.13 | HNO3 | Nitrate | S2,S5,S6,S7,S8,S1,S9 |
| 35.95 | N/A | 1089.7012 | -3.77 | C56H102N2O18 | Ganglioside GA2 (d18:1/9Z-18:1) | S2,S5,S6,S7,S8,S1,S9 |
| 35.95 | N/A | 943.7123 | 3.59 | C53H100O13 | DAT(16:0/23:0(2Me[S],4Me[S])) | S2,S5,S6,S7,S8,S1,S9 |
| 35.97 | N/A | 265.1481 | 1.53 | C12H26O4S | sodium dodecyl sulfate | S2,S5,S6,S7,S8,S1,S9 |
| 35.97 | N/A | 309.1720 | 4.71 | C17H26O5 | Botrydial | S2,S5,S6,S7,S8,S1,S9 |
| 35.99 | N/A | 115.9987 | 0.19 | C3H3NO4 | 3-Nitroacrylate | S2,S5,S6,S7,S8,S9 |
| 36.02 | N/A | 255.2328 | 0.25 | C16H32O2 | Hexadecanoic acid | S2,S5,S6,S7,S8,S1,S9 |
| 36.05 | N/A | 283.2649 | 3.10 | C18H36O2 | Octadecanoic acid | S2,S5,S6,S7,S8,S1,S9 |
| 36.06 | N/A | 213.0152 | 2.88 | C5H14N2O2Se | selenalysine | S2,S5,S6,S7,S8,S1,S9 |
| 36.12 | N/A | 138.0190 | -3.06 | C6H5NO3 | 6-Hydroxynicotinate | S2,S5,S6,S7,S8,S1,S9 |
| 36.12 | N/A | 183.0046 | 0.40 | C6H4N2O5 | 2,4-Dinitrophenol | S2,S5,S6,S7,S8,S1,S9 |
| 36.15 | N/A | 166.0507 | -0.23 | C8H9NO3 | Pyridoxal | S2,S5,S6,S7,S8,S1,S9 |
| 36.25 | N/A | 119.0361 | 0.06 | C5H4N4 | Purine | S2,S5,S6,S7,S8,S1,S9 |
| 36.34 | N/A | 116.9286 | 1.96 | CrH2O4 | chromate | S1,S6,S8,S9 |
| 36.41 | N/A | 118.9417 | -2.90 | H3O9P3 | Trimetaphosphate | S1,S6,S8,S9 |
| 38.74 | N/A | 116.9283 | 0.15 | CrH2O4 | chromate | S2,S5,S6,S7,S8,S1,S9 |
| **Positive ion mode - ZIC-HILIC Column** | | | | | | |
| 3.47 | 1.94 | 877.7260 | -2.54 | C57H96O6 | TG(54:7) | S2,S6,S5,S1,S9,S8,S7,S10 |
| 3.71 | 2.42 | 851.7107 | -2.12 | C55H94O6 | TG(52:6) | S2,S6,S5,S1,S9,S8,S7,S10 |
| 3.80 | 2.34 | 315.1230 | 0.20 | C18H18O5 | [Fv Hydroxy,trimethox] 4'-Hydroxy-2',4,6'-trimethoxychalcone | S2,S6,S5,S1,S9,S8,S7,S10 |
| 3.87 | 2.62 | 286.3111 | 1.41 | C18H39NO | [SP] 1-deoxy-sphinganine | S2,S6,S5,S1,S9,S8 |
| 3.88 | 2.77 | 813.6952 | -2.12 | C52H92O6 | [GL (16:0/16:0/17:2)] 1,2-di-(9Z-hexadecenoyl)-3-(9Z,12Z-heptadecadienoyl)-sn-glycerol | S2,S6,S1,S9,S8,S7,S10 |
| 3.89 | 2.28 | 849.6743 | -1.68 | C58H88O4 | 3-decaprenyl-4-hydroxy-5-methoxybenzoate | S6,S5 |
| 3.92 | 2.84 | 695.6536 | -2.11 | C44H86O5 | [GL (20:0/21:0)] 1-eicosanoyl-2-heneicosanoyl-sn-glycerol | S2,S6,S1,S9,S10 |
| 4.19 | 3.39 | 587.5767 | 0.48 | C40H74O2 | Behenyl linolenate | S2,S6,S5,S1,S9,S8,S7 |
| 4.32 | 3.64 | 493.3673 | -1.21 | C33H48O3 | [ST (3:0)] (5Z,7E)-(1S,3R,11S)-11-phenyl-9,10-seco-5,7,10(19)-cholestatriene-1,3,25-triol | S2,S6,S1,S9,S8,S7 |
| 4.35 | 3.70 | 369.3515 | -0.71 | C27H44 | [ST] (5Z,7E)-9,10-seco-5,7,10(19)-cholestatriene | S2,S6,S5,S1,S9,S8 |
| 4.38 | 3.56 | 673.5302 | 3.08 | C37H73N2O6P | SM(d18:2/14:0) | S2,S6,S5,S1,S9,S8,S7,S10 |
| 4.40 | 3.83 | 200.2011 | 0.04 | C12H25NO | Dodecanamide | S2,S6,S5,S1,S9,S8,S7,S10 |
| 4.45 | 3.89 | 445.2231 | 1.84 | C25H32O7 | [FA methyl,oxo(5:0/5:0)] methyl 4R,12S-diacetoxy-9-oxo-5Z,7E,10Z,13Z,17Z-prostapentaenoate-cyclo[8,12] | S2,S6,S5,S1,S9,S8,S10 |
| 4.45 | 3.41 | 493.3276 | -2.98 | C25H49O7P | PA(22:1(11Z)/0:0) | S2,S6,S1,S9,S8,S10 |
| 4.46 | 4.46 | 262.9945 | -1.30 | C10H15N4O15P3 | XTP | S9 |
| 4.47 | 4.47 | 449.2549 | 1.30 | C18H36N6O5S | Glutathionylaminopropylcadaverine | S2,S6,S5,S1,S9,S8,S7,S10 |
| 4.51 | 3.52 | 561.1590 | -2.76 | C27H28O13 | 3'-Deoxymaysin | S2,S6,S1,S8,S7,S10 |
| 4.62 | 3.24 | 768.4809 | -0.48 | C41H70NO10P | PS(15:0/20:5(5Z,8Z,11Z,14Z,17Z)) | S5,S1,S9,S8,S7,S10 |
| 4.62 | 4.58 | 239.2372 | 0.03 | C16H30O | 2-trans-Hexadecenal | S6,S5,S1,S9,S8,S7,S10 |
| 4.71 | 3.92 | 487.1239 | 0.35 | C24H22O11 | Epigallocatechin 3-O-(3,5-di-O-methylgallate) | S2,S6,S5,S1,S9,S8,S7,S10 |
| 4.72 | 3.43 | 744.4196 | 3.85 | C37H61NO14 | Demethyllactenocin | S7 |
| 4.74 | 3.11 | 215.0342 | 0.25 | C12H6O4 | Dibenzo[1,4]dioxin-2,3-dione | S6,S8,S7,S10 |
| 4.79 | 3.66 | 149.0598 | -0.94 | C9H8O2 | trans-Cinnamate | S2,S6,S5,S1,S9,S8,S7,S10 |
| 4.79 | 4.26 | 381.0981 | 2.57 | C21H16O7 | Diphyllin | S2,S6,S5,S1,S9,S8,S7,S10 |
| 4.79 | 4.09 | 459.0917 | -1.54 | C22H18O11 | Epigallocatechin 3-gallate | S2,S6,S5,S1,S9,S8,S7,S10 |
| 4.81 | 4.81 | 621.2910 | 0.38 | C32H44O12 | Lanceotoxin A | S2,S6,S5,S1,S9,S8,S7,S10 |
| 4.84 | 4.18 | 441.0819 | 0.04 | C22H16O10 | 6a,12b-Dihydro-3,10,11,12-tetrahydroxy-6-(3,4,5-trihydroxyphenyl)-[2]benzopyrano[3,4-c]benzopyran-8(6H)-one | S2,S6,S5,S1,S9,S8,S7,S10 |
| 4.85 | 4.20 | 585.1239 | -0.30 | C28H24O14 | 2''-O-Galloylisovitexin | S2,S6,S5,S1,S8,S7,S10 |
| 4.87 | 5.07 | 331.2841 | -1.18 | C19H38O4 | [GL (16:0)] 1-hexadecanoyl-rac-glycerol | S1,S9 |
| 4.91 | 4.32 | 471.0916 | -1.72 | C23H18O11 | 3,5,7-Tris(acetyloxy)-2-[4-(acetyloxy)-3-hydroxyphenyl]-4H-1-benzopyran-4-one | S2,S6,S5,S1,S9,S8,S7,S10 |
| 4.92 | 4.34 | 623.1779 | 2.81 | C32H30O13 | [Fv hydroxy,acetyl(4:0)] 4,2',3',4'-Tetrahydroxychalcone 4'-O-(2''-O-p-coumaroyl-6''-O-acetyl)glucoside | S2,S6,S5,S1,S9,S8,S7,S10 |
| 4.94 | 4.39 | 533.1292 | 0.08 | C25H24O13 | [Fv hydroxy,diacetyl(4:0/2:0)] 6,7,3',4'-Tetrahydroxyaurone 6-(4'',6''-diacetylglucoside) | S2,S6,S5,S1,S9,S8,S7,S10 |
| 4.96 | 4.41 | 389.0505 | -0.01 | C18H12O10 | Repenol | S2,S6,S5,S1,S9,S8,S7 |
| 4.99 | 4.86 | 315.1230 | 0.17 | C18H18O5 | [Fv Hydroxy,trimethox] 2'-Hydroxy-2,4',6'-trimethoxychalcone | S2,S6,S5,S1,S9,S7,S10 |
| 4.99 | 4.97 | 675.6078 | 0.21 | C47H78O2 | [ST (20:3)] cholest-5-en-3beta-yl (8Z,11Z,14Z-eicosatrienoate) | S6,S1,S9,S7 |
| 5.04 | 3.74 | 183.0782 | -0.31 | C6H15O4P | triethyl phosphate | S2,S6,S5,S1,S9,S8,S7,S10 |
| 5.05 | 3.17 | 297.0967 | -1.50 | C14H16O7 | dehypoxanthine futalosine | S2,S6,S5,S1,S9,S8,S7,S10 |
| 5.10 | 4.16 | 307.0453 | 0.67 | C14H10O8 | 2,2',3,3'-tetrahydroxy-5,5'-dicarboxybiphenyl | S2,S6,S5,S1,S8,S7,S10 |
| 5.16 | 4.83 | 323.0765 | 0.44 | C15H14O8 | Elephantorrhizol | S2,S6,S5,S1,S9,S8,S7,S10 |
| 5.18 | 4.85 | 403.1025 | -0.19 | C20H18O9 | Frangulin B | S2,S6,S5,S1,S9,S8,S7,S10 |
| 5.24 | 5.24 | 551.0693 | 3.02 | C15H24N2O16P2 | UDP-L-rhamnose | S2,S6,S1 |
| 5.30 | 5.30 | 522.1253 | 2.03 | C18H28N5O7PS2 | lipol-AMP | S2,S6,S5,S9,S8 |
| 5.32 | 5.12 | 288.2902 | 1.07 | C17H37NO2 | [SP (17:0)] heptadecasphinganine | S2,S6,S5,S1,S9,S8,S7,S10 |
| 5.34 | 5.56 | 286.3107 | 0.18 | C18H39NO | [SP] 1-deoxy-sphinganine | S2,S6,S5,S1,S9,S8 |
| 5.36 | 6.13 | 285.2790 | -0.11 | C18H36O2 | Octadecanoic acid | S2,S6,S5,S1,S9,S8,S7,S10 |
| 5.40 | 5.79 | 705.5830 | 1.57 | C47H76O4 | [ST (20:4)] cholest-5-en-3beta-yl (15S-hydroperoxy-5Z,8Z,12E,14Z-eicosatetraenoate) | S2,S6,S5,S1,S9,S8,S7,S10 |
| 5.44 | 5.87 | 995.8047 | -1.76 | C66H106O6 | [GL (19:0/22:5/22:6)] 1-nonadecanoyl-2-(7Z,10Z,13Z,16Z,19Z-docosapentaenoyl)-3-(4Z,7Z,10Z,13Z,16Z,19Z-docosahexaenoyl)-sn-glycerol | S6,S5,S8,S7,S10 |
| 5.48 | 5.96 | 511.4346 | -2.61 | C31H58O5 | DG(28:1) | S2,S6,S5,S1,S9,S7 |
| 5.52 | 5.53 | 973.6727 | -1.50 | C53H97O13P | PI(44:3) | S8,S7,S10 |
| 5.52 | 5.54 | 656.1928 | -3.23 | C29H35O17 | Malvidin 3-diglucoside | S2,S6,S5,S1,S8,S7,S10 |
| 5.52 | 5.54 | 783.5160 | -1.67 | C43H75O10P | PG(15:1(9Z)/22:4(7Z,10Z,13Z,16Z)) | S2,S6,S1,S8,S7,S10 |
| 5.54 | 5.58 | 939.5984 | 2.64 | C51H87O13P | PI(42:6) | S5,S9,S8,S7 |
| 5.56 | 5.62 | 596.1546 | 3.18 | C30H27O13 | Pelargonidin 3-(6''-caffeylglucoside) | S2,S6,S5,S1,S9,S8,S7 |
| 5.57 | 5.84 | 391.2849 | 0.99 | C24H38O4 | [ST hydrox] 3alpha,12alpha-Dihydroxy-5beta-chol-6-en-24-oic Acid | S2,S6,S5,S1,S9,S8,S7,S10 |
| 5.57 | 6.14 | 113.1328 | 0.83 | C8H16 | 3-methyl-1-heptene | S2,S6,S5,S1,S9,S8,S7,S10 |
| 5.58 | 6.55 | 371.3162 | 0.94 | C22H42O4 | 2-monooleoylglycerol | S2,S6,S5,S1,S9,S8,S7,S10 |
| 5.58 | 6.16 | 419.3158 | 0.00 | C26H42O4 | [ST (3:0)] (5Z,7E)-(1S,3R,24R)-22-oxa-9,10-seco-5,7,10(19)-cholestatriene-1,3,24-triol | S2,S6,S5,S1,S9,S8,S7,S10 |
| 5.59 | 6.18 | 575.4676 | 0.60 | C36H62O5 | DG(15:0/18:4(6Z,9Z,12Z,15Z)/0:0) | S6,S5,S1,S9,S8,S7,S10 |
| 5.60 | 6.19 | 470.4213 | -2.87 | C61H110O6 | TG(58:4) | S2 |
| 5.60 | 5.69 | 593.4194 | 2.56 | C31H61O8P | PA(28:0) | S2,S6,S5,S1,S9,S8,S7,S10 |
| 5.61 | 6.21 | 127.1484 | 0.43 | C9H18 | 1-Nonene | S5,S1,S9 |
| 5.61 | 6.48 | 229.2160 | -2.00 | C14H28O2 | Tetradecanoic acid | S9 |
| 5.61 | 5.72 | 775.4391 | -0.43 | C39H67O13P | PI(12:0/18:4(6Z,9Z,12Z,15Z)) | S2,S5,S9,S8,S7,S10 |
| 5.62 | 6.05 | 372.3109 | -0.55 | C21H41NO4 | Tetradecanoylcarnitine | S2 |
| 5.63 | 5.75 | 653.4764 | 1.52 | C34H69O9P | PG(O-16:0/12:0) | S6,S9,S8,S7 |
| 5.63 | 6.26 | 477.3592 | 3.21 | C29H48O5 | [ST hydroxy(3:0)] (5Z,7E)-(1R,2R,3R)-2-(2-hydroxyethoxy)-9,10-seco-5,7,10(19)-cholestatriene-1,3,25-triol | S2,S6,S5,S1,S9,S8,S7,S10 |
| 5.63 | 5.76 | 433.3694 | 3.59 | C28H48O3 | Cathasterone | S2,S6,S5,S1,S9,S8,S7,S10 |
| 5.63 | 6.26 | 573.4514 | -0.31 | C36H60O5 | DG(13:0/20:5(5Z,8Z,11Z,14Z,17Z)/0:0)[iso2] | S2,S6,S5,S1,S9,S8,S7,S10 |
| 5.63 | 5.77 | 853.5017 | 2.89 | C42H78O13P2 | PGP(18:2(9Z,12Z)/18:1(11Z)) | S2,S6,S5,S8,S7,S10 |
| 5.64 | 6.69 | 323.2955 | 2.62 | C21H38O2 | [FA methyl(20:2)] 19-methyl-5E,9E-eicosadienoic acid | S2,S6,S5,S1,S9,S8,S7,S10 |
| 5.64 | 6.62 | 257.2476 | -0.34 | C16H32O2 | Hexadecanoic acid | S2,S6,S5,S1,S9,S8,S7,S10 |
| 5.64 | 6.66 | 283.2635 | 0.26 | C18H34O2 | [FA (18:1)] 9Z-octadecenoic acid | S2,S6,S5,S1,S9,S8,S7,S10 |
| 5.64 | 6.27 | 433.3310 | -0.97 | C27H44O4 | [ST (2:0)] (7E)-(1S,3R,6R)-6,19-epidioxy-9,10-seco-5(10),7-cholestadiene-1,3-diol | S2,S6,S5,S1,S9,S8,S7,S10 |
| 5.64 | 6.74 | 353.3418 | 0.54 | C23H44O2 | [FA (23:1)] 22-tricosenoic acid | S2,S6,S1,S9,S8,S7,S10 |
| 5.64 | 5.78 | 835.5495 | 1.04 | C47H79O10P | PG(19:1(9Z)/22:6(4Z,7Z,10Z,13Z,16Z,19Z)) | S2,S6,S5,S8,S7,S10 |
| 5.64 | 6.70 | 309.2791 | 0.18 | C20H36O2 | Icosadienoic acid | S2,S6,S5,S1,S9,S8,S7,S10 |
| 5.65 | 5.79 | 503.3142 | -3.29 | C33H42O4 | Kolanone | S2,S6,S5,S1,S9,S8,S7,S10 |
| 5.65 | 6.30 | 547.4017 | 3.97 | C33H54O6 | cholest-5,24-dien-3beta-ol 3-O-beta-D-glucopyranoside | S2,S6,S5,S1,S9,S8,S7,S10 |
| 5.65 | 5.80 | 149.0235 | -0.34 | C16H8O6 | Medicagol | S2,S6,S5,S1,S9,S8,S7,S10 |
| 5.65 | 5.30 | 776.5418 | -2.62 | C41H78NO10P | PS(13:0/22:1(11Z)) | S2,S6,S5,S1,S9,S8,S7,S10 |
| 5.65 | 5.80 | 747.5159 | -1.88 | C40H75O10P | PG(34:2) | S2,S6,S5,S1,S9,S8,S7,S10 |
| 5.65 | 6.30 | 453.3357 | -1.78 | C30H44O3 | [ST dimethyl(4:0/3:0)] (5Z,7E,17E)-(1S,3R)-26,27-dimethyl-24a-homo-9,10-seco-5,7,10(19),17(20)-cholestatetraen-22-yne-1,3,25-triol | S2,S6,S5,S1,S9,S8,S7,S10 |
| 5.65 | 6.73 | 311.2949 | 0.83 | C20H38O2 | [FA (20:0)] 11Z-eicosenoic acid | S2,S6,S5,S1,S9,S8,S7,S10 |
| 5.65 | 6.40 | 279.1594 | 0.38 | C16H22O4 | 2-Ethylhexyl phthalate | S2,S6,S5,S1,S9,S8,S7,S10 |
| 5.65 | 6.31 | 489.3935 | -1.05 | C31H52O4 | [ST hydroxy(2:0)] (5Z,7E)-(1R,2R,3R)-2-(4-hydroxybutoxy)-9,10-seco-5,7,10(19)-cholestatriene-1,3-diol | S2,S5,S1,S9,S7,S10 |
| 5.66 | 5.42 | 167.0340 | -0.60 | C8H6O4 | Phthalate | S2,S6,S5,S1,S9,S8,S7,S10 |
| 5.66 | 6.31 | 511.3770 | -2.85 | C33H50O4 | [ST (3:0)] (7E)-(1R,2S,3R)-2-(benzyloxy)-19-nor-9,10-seco-5,7-cholestadiene-1,3,25-triol | S2,S1,S9,S8,S7,S10 |
| 5.66 | 6.31 | 453.3585 | 1.84 | C27H48O5 | [ST (5:0)] 5beta-Cholestane-3alpha,7alpha,12alpha,25,26-pentol | S2,S6,S5,S1,S9,S8,S7,S10 |
| 5.66 | 6.32 | 431.3521 | -0.26 | C28H46O3 | [ST methoxy(2:0)] (5Z,7E)-(1S,3R)-25-methoxy-9,10-seco-5,7,10(19)-cholestatriene-1,3-diol | S2,S6,S5,S1,S9,S8,S7,S10 |
| 5.66 | 6.32 | 583.3974 | -3.74 | C36H54O6 | [PR] OH-Diaponeurosporene glucoside ester | S2,S6,S5,S1,S9,S8,S7,S10 |
| 5.66 | 6.65 | 279.2322 | 0.34 | C18H30O2 | [FA (18:3)] 9Z,12Z,15Z-octadecatrienoic acid | S2,S6,S5,S1,S9,S8,S10 |
| 5.66 | 5.96 | 389.3045 | -1.91 | C25H40O3 | [PR (2:0)] (+)-24,25-epoxy-16-scalaren-12alpha,25alpha-diol | S2,S6,S5,S1,S9,S8,S7,S10 |
| 5.66 | 5.83 | 703.4540 | -1.00 | C37H67O10P | PG(13:0/18:3(6Z,9Z,12Z)) | S2,S6,S5,S1,S9,S8,S7,S10 |
| 5.66 | 5.83 | 425.3411 | -1.20 | C29H44O2 | alpha-Tocotrienol | S2,S6,S5,S1,S9,S8,S7,S10 |
| 5.67 | 6.69 | 281.2479 | 0.77 | C18H32O2 | Linoleate | S2,S6,S5,S1,S9,S8,S7,S10 |
| 5.67 | 5.83 | 401.3266 | 0.51 | C23H44O5 | 1,2-Didecanoylglycerol | S2 |
| 5.67 | 6.73 | 307.2637 | 1.00 | C20H34O2 | Icosatrienoic acid | S2,S6,S5,S1,S9,S8,S7,S10 |
| 5.67 | 6.63 | 243.2319 | -0.59 | C15H30O2 | [FA methyl(14:0)] 12-methyl-tetradecanoic acid | S2,S5,S1,S9 |
| 5.67 | 5.84 | 619.4336 | 0.09 | C33H63O8P | PA(30:1) | S6,S5,S1,S9,S8,S7,S10 |
| 5.67 | 6.35 | 425.3048 | -0.96 | C28H40O3 | [ST (6:0/6:0/3:0)] (5Z,7E,22E,24E,26E)-(1S,3R)-26a,26b-dihomo-27-nor-9,10-seco-5,7,10(19),22,24,26(26a)-cholestahexaene-1,3,26b-triol | S2,S6,S5,S1,S9,S8,S7,S10 |
| 5.67 | 5.85 | 719.4870 | 1.43 | C38H71O10P | PG(32:2) | S2,S6,S5,S1,S9,S8,S7,S10 |
| 5.67 | 6.35 | 449.3263 | -0.21 | C27H44O5 | [ST (3:0)] 25R-spirostan-2beta,3beta,6beta-triol | S2,S6,S5,S1,S9,S8,S7,S10 |
| 5.67 | 5.85 | 467.3147 | 2.65 | C23H47O7P | PA(20:0/0:0) | S2,S6,S5,S1,S9,S8,S7,S10 |
| 5.67 | 5.95 | 321.2785 | -1.76 | C21H36O2 | Pregnanediol | S2,S6,S5,S1,S9,S8,S7,S10 |
| 5.68 | 5.85 | 449.2909 | 1.94 | C26H40O6 | 16-feruloyloxypalmitate | S2,S6,S5,S1,S9,S8,S7,S10 |
| 5.68 | 5.86 | 469.3306 | -1.78 | C30H44O4 | 3-Oxoglycyrrhetinate | S2,S6,S5,S1,S9,S8,S7,S10 |
| 5.68 | 5.96 | 361.2182 | 1.79 | C22H29FO3 | 9-Fluoro-16alpha-methylpregn-4-ene-3,11,20-trione | S2,S6,S5,S1,S8,S7,S10 |
| 5.68 | 6.69 | 255.2311 | -3.95 | C16H30O2 | (9Z)-Hexadecenoic acid | S2,S6,S5,S1,S9,S8,S7,S10 |
| 5.68 | 5.87 | 923.6583 | -0.29 | C49H95O13P | PI(40:0) | S6,S5,S1,S9,S8,S7 |
| 5.69 | 5.87 | 415.3577 | 1.08 | C28H46O2 | 22alpha-Hydroxy-campest-4-en-3-one | S2,S6,S5,S1,S9,S8,S7,S10 |
| 5.69 | 6.38 | 609.4496 | -3.20 | C39H60O5 | DG(18 | S2,S6,S5,S1,S9,S7,S10 |
| 5.69 | 5.71 | 293.2470 | -2.44 | C19H32O2 | [ST (2:0)] androstan-3beta,17beta-diol | S2,S6,S5,S1,S9,S8,S7,S10 |
| 5.69 | 5.88 | 663.4217 | -2.50 | C34H63O10P | PG(14:1(9Z)/14:1(9Z)) | S5,S8,S7,S10 |
| 5.69 | 5.88 | 537.3198 | 1.71 | C26H49O9P | PG(20:2(11Z,14Z)/0:0) | S2,S6,S5,S1,S8,S7,S10 |
| 5.69 | 6.61 | 295.2271 | 0.26 | C18H30O3 | [FA oxo(5:1/5:0/8:0)] (1S,2S)-3-oxo-2-(2'Z-pentenyl)-cyclopentaneoctanoic acid | S2,S6,S5,S1,S9,S8,S7,S10 |
| 5.70 | 5.89 | 727.4536 | -1.46 | C39H67O10P | PG(13:0/20:5(5Z,8Z,11Z,14Z,17Z)) | S5,S8,S7,S10 |
| 5.70 | 6.39 | 463.3425 | 0.98 | C28H46O5 | [ST Trihydroxy,methy] 3alpha,7alpha,12alpha-Trihydroxy-24-methyl-5beta-cholest-23-en-26-oic acid | S2,S6,S5,S1,S9,S8,S7,S10 |
| 5.70 | 6.75 | 379.2839 | -1.68 | C23H38O4 | 2-Arachidonoylglycerol | S2,S6,S5,S1,S9,S8,S7,S10 |
| 5.70 | 6.40 | 599.4648 | -4.04 | C38H62O5 | DG(13:0/22:6(4Z,7Z,10Z,13Z,16Z,19Z)/0:0)[iso2] | S2,S6,S5,S1,S9,S8,S7,S10 |
| 5.70 | 5.91 | 559.3052 | -0.81 | C35H42O6 | Denticulaflavonol | S2,S6,S5,S1,S9,S8,S7,S10 |
| 5.70 | 6.41 | 397.3865 | 1.24 | C51H100O5 | DG(24:0/24:0/0:0) | S2,S6,S5,S1,S9,S8,S7,S10 |
| 5.70 | 5.91 | 625.4083 | 0.91 | C31H61O10P | PG(13:0/12:0) | S2,S6,S5,S1,S9,S8,S7,S10 |
| 5.71 | 6.12 | 315.2316 | -1.45 | C21H30O2 | Progesterone | S2,S6,S5,S1,S9,S8,S7,S10 |
| 5.71 | 5.91 | 429.3728 | -0.38 | C29H48O2 | (24R,24'R)-Fucosterol epoxide | S2,S6,S5,S1,S9,S8,S7,S10 |
| 5.71 | 6.81 | 359.3158 | 0.03 | C21H42O4 | [GL (18:0)] 1-octadecanoyl-rac-glycerol | S2,S6,S5,S1,S9,S8,S7,S10 |
| 5.71 | 5.91 | 651.4240 | 0.94 | C33H63O10P | PG(12:0/15:1(9Z)) | S2,S6,S5,S1,S9,S8,S7,S10 |
| 5.71 | 6.82 | 354.3368 | -0.29 | C22H43NO2 | [FA (20:0)] N-(11Z-eicosaenoyl)-ethanolamine | S2,S6,S5,S1,S9,S8,S7,S10 |
| 5.71 | 6.21 | 359.2582 | -0.16 | C23H34O3 | Pregnenolone acetate | S2,S6,S5,S1,S9,S8,S7,S10 |
| 5.71 | 5.92 | 539.3357 | 2.15 | C26H51O9P | PG(20:1(11Z)/0:0) | S2,S6,S5,S1,S9,S8,S7,S10 |
| 5.71 | 6.48 | 301.2165 | 0.33 | C20H28O2 | [PR] Tretinoin/All-Trans Retinoic Acid | S2,S6,S5,S1,S9,S8,S7,S10 |
| 5.71 | 6.71 | 297.2424 | -0.74 | C18H32O3 | [FA hydroxy(18:2)] 9S-hydroxy-10E,12Z-octadecadienoic acid | S2,S6,S5,S1,S9,S8,S7,S10 |
| 5.71 | 6.42 | 457.3311 | -0.75 | C29H44O4 | [ST dimethyl(4:0/4:0/4:0)] (5Z,7E)-(1S,3R,22S)-26,27-dimethyl-9,10-seco-5,7,10(19)-cholestatrien-23-yne-1,3,22,25-tetrol | S2,S6,S5,S1,S9,S8,S7,S10 |
| 5.71 | 6.16 | 389.2688 | -0.04 | C24H36O4 | [ST hydroxy(3:0)] (5Z,7E)-(1S,3R)-1,3-dihydroxy-9,10-seco-5,7,10(19)-cholatrien-24-oic acid | S2,S6,S5,S1,S9,S8,S7,S10 |
| 5.71 | 6.14 | 377.2681 | -1.91 | C23H36O4 | 3-Acetyl-5alpha-androstane-3beta,17beta-diol 3-acetate | S2,S6,S5,S1,S9,S8,S7,S10 |
| 5.71 | 6.80 | 284.2953 | 0.88 | C18H37NO | Octadecanamide | S2,S6,S5,S1,S9,S8,S7,S10 |
| 5.72 | 5.93 | 677.4731 | -3.42 | C36H69O9P | PG(P-16:0/14:1(9Z)) | S2,S6,S5,S1,S9,S8,S7,S10 |
| 5.72 | 6.82 | 352.3213 | 0.34 | C22H41NO2 | [FA (20:2)] N-(11Z,14Z-eicosadienoyl)-ethanolamine | S2,S6,S5,S1,S9,S8,S7,S10 |
| 5.72 | 5.94 | 629.4187 | 1.20 | C34H61O8P | PA(13:0/18:3(6Z,9Z,12Z)) | S2,S6,S5,S1,S9,S8,S7,S10 |
| 5.72 | 5.94 | 763.5500 | 1.85 | C41H79O10P | PG(13:0/22:1(11Z)) | S2,S6,S5,S1,S9,S8,S7,S10 |
| 5.72 | 6.44 | 617.4204 | 0.18 | C40H56O5 | [PR] Fucoxanthinol | S2,S6,S5,S1,S9,S8,S7,S10 |
| 5.72 | 5.94 | 735.5193 | 2.68 | C39H75O10P | PG(13:0/20:1(11Z)) | S2,S6,S5,S1,S9,S8,S7,S10 |
| 5.72 | 6.63 | 219.2108 | -0.62 | C16H26 | TMTT | S2,S6,S5,S1,S9,S8,S7,S10 |
| 5.72 | 6.39 | 331.2631 | -0.97 | C22H34O2 | Taxa-4(20),11(12)-dien-5alpha-yl acetate | S2,S6,S5,S1,S9,S8,S7,S10 |
| 5.72 | 5.94 | 757.5019 | 0.29 | C41H73O10P | PG(13:0/22:4(7Z,10Z,13Z,16Z)) | S2,S6,S5,S1,S9,S8,S7,S10 |
| 5.72 | 5.95 | 659.4649 | 0.06 | C36H67O8P | PA(13:0/20:2(11Z,14Z)) | S2,S6,S5,S1,S9,S8,S7,S10 |
| 5.72 | 5.95 | 481.2945 | 3.73 | C23H45O8P | [GP (10:0)] 1,2-didecanoyl-sn-glycero-3-phosphate | S2,S6,S5,S1,S9,S8,S7,S10 |
| 5.73 | 5.45 | 655.3877 | -1.80 | C35H60O7P2 | all-trans-Heptaprenyl diphosphate | S2,S6,S5,S1,S9,S8,S7,S10 |
| 5.73 | 5.96 | 361.2735 | -1.22 | C23H36O3 | [ST (3:0)] (5Z,7E)-(1S,3R)-24-nor-9,10-seco-5,7,10(19)-cholatriene-1,3,23-triol | S2,S6,S5,S1,S9,S8,S7,S10 |
| 5.73 | 5.87 | 329.2117 | 1.15 | C21H28O3 | 9,11alpha-Epoxypregn-4-ene-3,20-dione | S2,S6,S5,S1,S9,S8,S7,S10 |
| 5.73 | 6.72 | 399.2513 | 1.14 | C18H39O7P | Tris(butoxyethyl)phosphate | S2,S6,S5,S1,S9,S8,S7,S10 |
| 5.73 | 6.80 | 303.2321 | 0.17 | C20H30O2 | [FA (20:5)] 5Z,8Z,11Z,14Z,17Z-eicosapentaenoic acid | S2,S6,S5,S1,S9,S8,S7,S10 |
| 5.73 | 6.73 | 344.2792 | -1.53 | C19H37NO4 | 1,2-dioctanoyl-1-amino-2,3-propanediol | S2,S6,S5,S1,S9 |
| 5.74 | 6.82 | 282.2796 | 0.75 | C18H35NO | [FA (18:1)] 9Z-octadecenamide | S2,S6,S5,S1,S9,S8,S7,S10 |
| 5.74 | 6.47 | 477.3204 | -1.79 | C28H44O6 | [PR] (-)-Asbestinine 2 | S2,S6,S5,S1,S9,S8,S7,S10 |
| 5.74 | 5.97 | 553.1713 | 1.15 | C29H28O11 | Cucumerin B | S2,S6,S5,S1,S9,S8,S7 |
| 5.74 | 5.98 | 833.6794 | -1.79 | C58H88O3 | 2-decaprenyl-3-methyl-6-methoxy-1,4-benzoquinone | S6,S5,S8,S7,S10 |
| 5.74 | 5.78 | 212.0833 | -2.56 | C11H14ClNO | propachlor | S10 |
| 5.74 | 6.79 | 256.2639 | 0.69 | C16H33NO | Palmiticamide | S2,S6,S5,S1,S9,S8,S7,S10 |
| 5.74 | 5.99 | 847.5693 | -0.53 | C45H83O12P | PI(O-18:0/18:3(6Z,9Z,12Z)) | S2,S6,S5,S9,S8,S7,S10 |
| 5.75 | 6.06 | 170.0963 | -2.31 | C12H11N | Diphenylamine | S6,S9,S8 |
| 5.75 | 5.32 | 117.0185 | 0.61 | C4H4O4 | Fumarate | S6,S1,S8 |
| 5.75 | 6.00 | 771.5183 | 1.31 | C42H75O10P | PG(36:4) | S2,S6,S5,S1,S9,S8,S7,S10 |
| 5.75 | 6.05 | 331.2270 | -0.09 | C21H30O3 | [ST hydroxy(2:0)] 21-hydroxypregn-4-ene-3,20-dione | S2,S6,S5,S1,S9,S8,S7,S10 |
| 5.75 | 6.50 | 457.2941 | -2.22 | C28H40O5 | Minabeolide-7 | S2,S6,S5,S1,S9,S8,S7,S10 |
| 5.75 | 6.00 | 503.3350 | -3.78 | C30H46O6 | 16-alpha-hydroxygypsogenate | S2,S6,S5,S1,S9,S8,S7,S10 |
| 5.75 | 6.51 | 499.3052 | -0.98 | C30H42O6 | Cucurbitacin S | S2,S6,S5,S1,S9,S8,S7,S10 |
| 5.76 | 6.16 | 315.1960 | 1.03 | C20H26O3 | momilactone A | S2,S6,S5,S1,S9,S8,S7,S10 |
| 5.76 | 6.01 | 485.3258 | -1.20 | C30H44O5 | Propapyriogenin A2 | S2,S6,S5,S1,S9,S8,S7,S10 |
| 5.76 | 6.01 | 479.2794 | -0.09 | C30H38O5 | [Fv hydroxy(4:0)] 3-Geranyl-4,2',4',6'-tetrahydroxy-5-prenyldihydrochalcone | S2,S6,S5,S1,S9,S8,S7,S10 |
| 5.76 | 6.02 | 557.2897 | 3.80 | C28H45O9P | PG(22:6(4Z,7Z,10Z,13Z,16Z,19Z)/0:0) | S2,S6,S5,S1,S9,S8,S7,S10 |
| 5.76 | 6.87 | 241.2527 | -0.31 | C16H32O | Hexadecanal | S2,S5,S1,S8 |
| 5.76 | 6.07 | 317.2465 | -3.91 | C21H32O2 | [ST hydrox] 3beta-hydroxypregn-5-ene-20-one | S2,S6,S5,S1,S9,S8,S7,S10 |
| 5.77 | 6.80 | 355.2260 | -2.95 | C23H30O3 | [PR] Etretinate | S2,S6,S5,S1,S9,S8,S7,S10 |
| 5.77 | 6.59 | 379.2273 | 0.92 | C25H30O3 | 17beta-(Benzoyloxy)-B-norandrost-4-en-3-one | S2,S6,S5,S1,S9,S8,S7,S10 |
| 5.77 | 6.04 | 755.5570 | -2.36 | C43H79O8P | PA(40:3) | S2,S6,S5,S1,S8,S7,S10 |
| 5.77 | 6.90 | 373.2944 | -1.79 | C21H40O5 | 1,2-dioctanoyl-3-methyl-1,2,3-butanetriol | S2,S6,S5,S1,S9,S8,S7,S10 |
| 5.77 | 6.84 | 321.2422 | -1.30 | C20H32O3 | [FA hydroxy(20:4)] 15S-hydroxy-5Z,8Z,11Z,13E-eicosatetraenoic acid | S2,S6,S5,S1,S9,S8,S7,S10 |
| 5.77 | 6.54 | 185.1537 | -0.51 | C11H20O2 | [FA (11:1)] 10-undecenoic acid | S2,S6,S5,S1,S9,S8,S7,S10 |
| 5.77 | 6.04 | 439.3943 | 1.44 | C31H50O | (24R)-24-Methylcycloarta-25-en-3-beta-ol | S2,S6,S5,S1,S9,S8,S7,S10 |
| 5.78 | 6.05 | 419.2788 | -1.54 | C25H38O5 | 3alpha,12alpha-Dihydroxy-5beta-pregnan-20-one diacetate | S2,S6,S5,S1,S9,S8,S7,S10 |
| 5.78 | 5.57 | 483.2735 | 3.23 | C22H43O9P | 2-16:1-lysoPG | S2,S6,S5,S1,S9,S8,S7,S10 |
| 5.78 | 6.07 | 811.6194 | -2.46 | C47H87O8P | PA(44:3) | S2,S6,S5,S1,S9,S8,S7,S10 |
| 5.79 | 6.57 | 529.2931 | 0.77 | C27H45O8P | 2-deoxyecdysone 22-phosphate | S2,S6,S5,S1,S9,S8,S7,S10 |
| 5.79 | 6.58 | 417.2634 | -1.02 | C25H36O5 | [PR] Manoalide | S2,S6,S5,S1,S9,S8,S7,S10 |
| 5.79 | 6.58 | 214.2167 | -0.12 | C13H27NO | Tridecanamide | S2,S6,S5,S1,S9,S8,S7,S10 |
| 5.79 | 6.51 | 389.2323 | -0.37 | C23H32O5 | [FA trihydroxy(2:0)] 9S,11R,15S-trihydroxy-17-phenyl-18,19,20-trinor-5Z,13E-prostadienoic acid | S2,S6,S5,S1,S9,S8,S7,S10 |
| 5.79 | 6.87 | 391.2476 | -1.48 | C23H34O5 | [FA methyl,oxo,hydroxy(3:0)] methyl 9-oxo-11R-hydroxy-15R-acetoxy-5Z,10,13E-prostatrienoate | S2,S6,S5,S1,S9,S8,S7,S10 |
| 5.79 | 5.59 | 653.2978 | 0.84 | C37H40N4O7 | Haplophytine | S2,S6,S5,S9 |
| 5.79 | 6.79 | 228.2321 | -1.32 | C14H29NO | myristic amide | S2,S6,S5,S1,S9,S8,S7,S10 |
| 5.79 | 6.52 | 253.1225 | 0.03 | C17H16O2 | cis-Hinokiresinol | S2,S6,S5,S1,S9,S8,S7,S10 |
| 5.80 | 6.77 | 335.2216 | -0.93 | C20H30O4 | Prostaglandin A2 | S2,S6,S5,S1,S9,S8,S7,S10 |
| 5.80 | 6.10 | 439.2472 | -2.19 | C27H34O5 | 16alpha,17-Dihydroxypregn-4-ene-3,20-dione cyclic acetal with 2-furyl methyl ketone | S2,S6,S5,S1,S9,S8,S7,S10 |
| 5.80 | 5.60 | 419.2445 | 0.34 | C25H30N4O2 | Naphthyl dipeptide | S2,S6,S5,S1,S9,S8,S7,S10 |
| 5.80 | 6.10 | 579.4402 | -1.44 | C38H58O4 | 2-Hexaprenyl-3-methyl-5-hydroxy-6-methoxy-1-4-benzoquinol | S2,S6,S5,S1,S9,S8,S7,S10 |
| 5.80 | 6.11 | 615.4792 | 2.91 | C42H62O3 | 3-heptaprenyl-4-hydroxybenzoate | S2,S6,S5,S9,S8 |
| 5.80 | 6.38 | 165.0700 | -0.70 | C13H8 | 1-Phenylhepta-1,3,5-triyne | S2,S6,S5,S9,S8,S7,S10 |
| 5.80 | 6.11 | 433.2590 | 0.63 | C25H36O6 | Glycinoeclepin A | S2,S6,S5,S1,S9,S8,S7,S10 |
| 5.80 | 6.11 | 437.2316 | -2.01 | C27H32O5 | [Fv (9:1)] Lespedezaflavanone F | S2,S6,S5,S1,S9,S8,S7,S10 |
| 5.80 | 6.11 | 743.4847 | -1.72 | C40H71O10P | PG(34:4) | S2,S6,S5,S1,S9,S8,S7,S10 |
| 5.80 | 5.52 | 265.0497 | -0.14 | C16H8O4 | Frutinone A | S2,S6,S5,S8,S7 |
| 5.81 | 6.17 | 205.0860 | -0.55 | C12H12O3 | 3-Butylidene-7-hydroxyphthalide | S2,S6,S5,S1,S9,S8,S7,S10 |
| 5.81 | 6.64 | 197.1323 | -2.11 | C15H16 | 1,3-Diphenylpropane | S2,S6,S5,S1,S9,S8,S7,S10 |
| 5.81 | 6.13 | 415.2116 | -0.35 | C24H30O6 | Magnoshinin | S2,S6,S5,S1,S9,S8,S7,S10 |
| 5.82 | 6.22 | 219.1745 | -0.30 | C15H22O | Solavetivone | S2,S6,S5,S1,S9,S8,S7,S10 |
| 5.82 | 6.14 | 691.4536 | -1.57 | C36H67O10P | PG(30:2) | S2,S6,S5,S1,S9,S8,S7,S10 |
| 5.82 | 4.48 | 189.0395 | -0.47 | C7H8O6 | (Z)-But-2-ene-1,2,3-tricarboxylate | S8 |
| 5.82 | 7.06 | 333.2786 | -1.23 | C22H36O2 | [FA (22:4)] 7Z,10Z,13Z,16Z-docosatetraenoic acid | S2,S6,S5,S1,S9,S8,S7,S10 |
| 5.82 | 6.84 | 327.1591 | -0.50 | C20H22O4 | [Fv Trihydrox] 2',4',6'-Trihydroxy-3'-prenyldihydrochalcone | S2,S6,S5,S1,S9,S8,S7,S10 |
| 5.82 | 6.65 | 407.2426 | -1.01 | C23H34O6 | diginatigenin | S2,S6,S5,S1,S9,S8,S7,S10 |
| 5.83 | 6.15 | 723.4094 | 2.21 | C38H54N6O8 | Myxochromide S1 | S2,S6,S5,S1,S9,S8,S7,S10 |
| 5.83 | 5.93 | 347.2216 | -0.88 | C21H30O4 | [ST hydroxy(2:0)] 17,21-dihydroxypregn-4-ene-3,20-dione | S2,S6,S5,S1,S9,S8,S7,S10 |
| 5.83 | 5.16 | 653.2610 | 0.31 | C36H36N4O8 | Dehydroisocoproporphyrinogen | S2,S6,S5,S9,S8,S7,S10 |
| 5.83 | 6.89 | 291.1956 | -0.51 | C18H26O3 | [FA hydroxy(18:1/2:0)] 8-hydroxy-13Z-octadecene-9,11-diynoic acid | S2,S6,S5,S1,S9,S8,S7,S10 |
| 5.83 | 6.16 | 441.2266 | -1.77 | C26H32O6 | [Fv hydroxy,methoxy(4:0/2:0)] 3,2',4',6'-Tetrahydroxy-4-methoxy-3',5-diprenyldihydrochalcone | S2,S6,S5,S1,S9,S8,S7,S10 |
| 5.83 | 5.48 | 170.0364 | -2.99 | C8H8NOCl | 4-Chloroacetanilide | S8,S7 |
| 5.83 | 4.74 | 256.2279 | 2.14 | C15H29NO2 | 9-Decenoylcholine | S2,S6,S5,S1,S9,S8,S7,S10 |
| 5.83 | 6.67 | 531.2961 | 1.27 | C30H42O8 | Proscillaridin A | S2,S6,S1,S9,S8,S7,S10 |
| 5.83 | 6.03 | 333.2417 | -2.96 | C21H32O3 | 17alpha,20alpha-Dihydroxypregn-4-en-3-one | S6,S5,S1,S9,S8,S7,S10 |
| 5.84 | 5.67 | 812.5207 | -2.47 | C47H74NO8P | PE(42:10) | S2,S6,S5,S1,S9,S8,S7,S10 |
| 5.84 | 6.56 | 359.9831 | -2.09 | C14H8FNO5Cl2 | MC-7181 | S5 |
| 5.84 | 5.69 | 529.2216 | -1.42 | C32H32O7 | Karwinskione | S2,S6,S5,S7,S10 |
| 5.84 | 5.96 | 227.1068 | -0.41 | C15H14O2 | (2S)-Flavan-4-ol | S2,S6,S5,S1,S9,S8,S7,S10 |
| 5.85 | 6.77 | 203.1797 | 0.09 | C15H22 | alpha-Curcumene | S2,S6,S5,S1,S9,S8,S7,S10 |
| 5.85 | 6.69 | 477.3789 | 0.13 | C26H52O7 | [FA (20:0)] 1-O-alpha-D-glucopyranosyl-eicosan-2-ol | S8 |
| 5.85 | 5.80 | 289.1801 | 0.22 | C18H24O3 | Estriol | S2,S6,S5,S1,S9,S8,S7,S10 |
| 5.85 | 5.70 | 526.3366 | -2.06 | C28H47NO8 | Pikromycin | S2,S6,S5,S1,S9,S8,S7,S10 |
| 5.85 | 6.50 | 536.5053 | 2.61 | C34H65NO3 | Cer(d18:2/16:0) | S5,S1,S9 |
| 5.85 | 6.70 | 255.1953 | -1.65 | C15H26O3 | formyl 7-oxo-11E-tetradecenoate | S2,S6,S5,S1,S9,S8,S7 |
| 5.85 | 6.20 | 459.2377 | -0.56 | C26H34O7 | Fumagillin | S2,S6,S5,S1,S9,S8,S7,S10 |
| 5.85 | 4.01 | 221.0600 | 3.38 | C7H12N2O4S | AMCC | S2,S6,S5,S1,S9,S8,S7 |
| 5.85 | 4.79 | 369.2637 | -0.15 | C21H36O5 | Cortol | S2,S6,S5,S1,S9,S8,S7,S10 |
| 5.86 | 6.74 | 200.2011 | 0.12 | C12H25NO | Dodecanamide | S2,S6,S5,S1,S9,S8,S7,S10 |
| 5.86 | 5.11 | 171.1382 | -0.13 | C10H18O2 | [PR] Limonene-1,2-diol | S2,S6,S5,S1,S8,S10 |
| 5.86 | 6.53 | 399.1081 | 1.11 | C21H18O8 | Dihydro-NAME | S2,S6,S5,S1,S9,S8,S7,S10 |
| 5.86 | 6.72 | 447.2380 | -0.01 | C25H34O7 | [FA methyl,oxo(4:0/4:0)] methyl 4R,12S-diacetoxy-9-oxo-5Z,7E,10Z,13Z-prostatetraenoate-cyclo[8,12] | S2,S6,S5,S1,S9,S8,S7,S10 |
| 5.86 | 6.09 | 217.0861 | -0.26 | C13H12O3 | Nepodin | S2,S6,S5,S1,S9,S8,S7,S10 |
| 5.86 | 3.71 | 149.0813 | 1.42 | C6H12O4 | [FA methyl,hydroxy(5:0)] 3R-methyl-3,5-dihydroxy-pentanoic acid | S2,S6,S5,S1,S8,S7 |
| 5.86 | 6.22 | 489.2116 | -1.01 | C26H32O9 | Ichangin | S2,S6,S5,S1,S9,S8,S7,S10 |
| 5.86 | 6.22 | 423.2379 | -0.23 | C23H34O7 | Picrasin C | S2,S6,S5,S1,S9,S8,S7,S10 |
| 5.86 | 6.75 | 221.1900 | -0.99 | C15H24O | 2-trans,6-trans-Farnesal | S2,S6,S5,S1,S9,S8,S7,S10 |
| 5.86 | 5.86 | 449.2539 | -0.96 | C18H36N6O5S | Glutathionylaminopropylcadaverine | S2,S6,S1,S9,S8,S7,S10 |
| 5.86 | 5.96 | 203.1068 | -0.20 | C13H14O2 | Tremetone | S2,S6,S5,S1,S9,S8,S7,S10 |
| 5.86 | 6.94 | 309.2061 | -0.40 | C18H28O4 | 5-O-Methylembelin | S2,S6,S5,S1,S9,S8,S7,S10 |
| 5.86 | 5.49 | 365.2324 | -0.09 | C21H32O5 | Urocortisone | S2,S6,S5,S1,S9,S8,S7,S10 |
| 5.87 | 6.54 | 213.1275 | -0.33 | C15H16O | p-Cumylphenol | S2,S6,S5,S1,S9,S8,S7,S10 |
| 5.87 | 6.23 | 433.2003 | -2.12 | C27H28O5 | Aspulvinone H | S2,S6,S5,S1,S9,S8,S7,S10 |
| 5.87 | 6.69 | 171.1169 | -0.80 | C13H14 | Aethusin | S2,S6,S5,S1,S9,S8,S7,S10 |
| 5.87 | 5.19 | 393.2269 | -1.22 | C22H32O6 | Isodomedin | S2,S6,S5,S1,S9,S8,S7,S10 |
| 5.87 | 6.84 | 351.2165 | -0.82 | C20H30O5 | 15-Keto-prostaglandinE2 | S2,S6,S5,S1,S9,S8,S7,S10 |
| 5.87 | 6.09 | 331.1907 | 0.17 | C20H26O4 | Gibberellin A15 | S2,S6,S5,S1,S9,S8,S7,S10 |
| 5.87 | 6.01 | 227.0704 | -0.58 | C14H10O3 | Anthralin | S2,S6,S5,S1,S9,S8,S7,S10 |
| 5.88 | 6.34 | 235.1693 | -0.84 | C15H22O2 | [PR] 1,13-Dihydroxy-herbertene | S2,S6,S5,S1,S9,S8,S7,S10 |
| 5.88 | 5.62 | 237.1850 | -0.54 | C15H24O2 | Capsidiol | S2,S6,S5,S1,S9,S8,S7,S10 |
| 5.89 | 6.48 | 275.1644 | 0.04 | C17H22O3 | Podocarpic acid | S2,S6,S5,S1,S9,S8,S7,S10 |
| 5.89 | 6.27 | 639.3539 | 1.44 | C37H50O9 | Pimelea factor P2 | S2,S6,S5,S9,S8,S7,S10 |
| 5.89 | 5.85 | 253.0498 | 0.26 | C15H8O4 | Morindaparvin A | S2,S6,S5,S1,S8,S7 |
| 5.89 | 6.78 | 189.1276 | 0.04 | C13H16O | [FA (13:1/2:0)] 2-tridecene-4,7-diynal | S2,S6,S5,S1,S9,S8,S7,S10 |
| 5.89 | 5.73 | 351.2527 | -1.54 | C21H34O4 | [ST trihydrox] 3alpha,11beta,21-5alpha-trihydroxy-pregnane-20-one | S2,S6,S5,S1,S9,S8,S7,S10 |
| 5.89 | 6.26 | 289.1438 | 0.37 | C17H20O4 | Karwinaphthol B | S2,S6,S5,S1,S9,S8,S7,S10 |
| 5.89 | 4.87 | 253.1799 | -0.46 | C15H24O3 | [PR] (+)-2-Sterpurene-6,12,15-triol | S2,S6,S5,S1,S9,S8,S7,S10 |
| 5.90 | 6.79 | 426.2852 | -0.10 | C23H39NO6 | [FA hydroxy,hydroxy,oxo(3:0/2:0)] N-(1,3-dihydroxypropan-2-yl)-9S,15S-dihydroxy-11-oxo-5Z,13E-prostadienoyl amine | S2,S6,S5,S1,S9,S8,S7,S10 |
| 5.90 | 6.40 | 277.1799 | -0.51 | C17H24O3 | Buddledin A | S2,S6,S5,S1,S9,S8,S7,S10 |
| 5.90 | 6.83 | 353.2313 | -3.22 | C20H32O5 | [FA oxo,hydroxy(2:0)] 9-oxo-11R,15S-dihydroxy-5Z,13E-prostadienoic acid | S2,S6,S5,S1,S9,S8,S7,S10 |
| 5.90 | 6.28 | 373.1284 | -0.09 | C20H20O7 | Sesamolinol | S2,S6,S5,S1,S9,S8,S7,S10 |
| 5.90 | 6.31 | 433.2225 | 0.33 | C24H32O7 | Inflexin | S2,S6,S5,S1,S9,S8,S7,S10 |
| 5.91 | 5.99 | 305.1386 | -0.06 | C17H20O5 | Angustibalin | S2,S6,S5,S1,S9,S8,S7,S10 |
| 5.91 | 6.32 | 503.2270 | -1.60 | C27H34O9 | Verrucarin A | S2,S6,S5,S1,S9,S8,S7,S10 |
| 5.91 | 6.44 | 333.2062 | -0.33 | C20H28O4 | Gibberellin A12 | S2,S6,S5,S1,S9,S8,S7,S10 |
| 5.92 | 6.33 | 833.6599 | -3.96 | C47H93O9P | PG(P-20:0/21:0) | S6,S5,S1,S8,S7,S10 |
| 5.92 | 6.33 | 401.1594 | -0.86 | C22H24O7 | [Fv] Melafolone | S2,S6,S5,S1,S9,S8,S7,S10 |
| 5.92 | 4.25 | 175.0236 | -1.74 | C6H6O6 | trans-Aconitate | S2,S8 |
| 5.92 | 6.86 | 295.1904 | -0.62 | C17H26O4 | [6]-Gingerol | S2,S6,S5,S1,S9,S8,S7,S10 |
| 5.92 | 6.09 | 221.1537 | -0.38 | C14H20O2 | Oblongolide | S2,S6,S5,S1,S9,S8,S7,S10 |
| 5.92 | 6.34 | 747.6237 | -3.72 | C43H87O7P | PA(O-20:0/20:0) | S2,S6,S5,S1,S8,S7,S10 |
| 5.92 | 3.65 | 107.0706 | 1.07 | C4H10O3 | Diethylene glycol | S2,S6,S5,S1,S9,S8 |
| 5.92 | 4.24 | 157.0134 | 0.19 | C6H4O5 | 2-5-Furandicarboxylicacid | S2,S8,S7 |
| 5.93 | 6.45 | 225.1487 | -0.32 | C13H20O3 | [FA] Methyl jasmonate | S2,S6,S5,S1,S9,S8,S7,S10 |
| 5.93 | 6.35 | 745.5727 | -2.32 | C42H81O8P | PA(17:0/22:1(11Z)) | S2,S6,S5,S9,S8,S7,S10 |
| 5.93 | 5.33 | 163.0391 | -0.64 | C9H6O3 | Umbelliferone | S2,S6,S5,S1,S9,S8,S7,S10 |
| 5.93 | 6.36 | 409.2220 | -0.76 | C22H32O7 | Cascarillin | S2,S6,S5,S1,S9,S8,S7,S10 |
| 5.93 | 6.52 | 271.1895 | -4.09 | C15H26O4 | (10S)-Juvenile hormone III acid diol | S2,S6,S5,S1,S9,S8 |
| 5.93 | 5.15 | 162.0551 | -0.72 | C9H7NO2 | 4,6-Dihydroxyquinoline | S2,S1,S9 |
| 5.94 | 6.76 | 289.1069 | -1.25 | C16H16O5 | Asebogenin | S2,S6,S5,S1,S9,S8,S7,S10 |
| 5.94 | 6.37 | 699.4080 | -0.13 | C33H63O13P | PI(12:0/12:0) | S2,S6,S5,S1,S9,S8,S7,S10 |
| 5.94 | 6.60 | 259.0966 | -0.26 | C15H14O4 | [Fv] Davidigenin | S2,S6,S5,S1,S9,S8,S7,S10 |
| 5.94 | 6.16 | 113.0964 | 0.71 | C7H12O | [FA (7:1)] 2-heptenal | S2,S6,S5,S1,S9,S8,S7,S10 |
| 5.94 | 6.87 | 519.2015 | -0.13 | C30H30O8 | Gossypol | S2,S6,S5,S1,S9,S8,S7,S10 |
| 5.94 | 5.87 | 153.1275 | -0.60 | C10H16O | Perillyl alcohol | S2,S6,S5,S1,S9,S8,S7,S10 |
| 5.94 | 5.38 | 583.2547 | -1.08 | C33H34N4O6 | Biliverdin | S2,S6,S5,S1,S9,S8,S10 |
| 5.94 | 5.88 | 616.3126 | 1.15 | C33H45NO10 | Hypaconitine | S6,S9,S8,S10 |
| 5.94 | 6.88 | 172.1698 | -0.31 | C10H21NO | decanamide | S2,S6,S5,S1,S9,S8,S7,S10 |
| 5.94 | 5.85 | 251.1643 | -0.20 | C15H22O3 | Xanthoxin | S2,S6,S5,S1,S9,S8,S7,S10 |
| 5.94 | 5.80 | 169.0860 | -0.74 | C9H12O3 | 1,3,5-trimethoxybenzene | S2,S6,S5,S1,S9,S8,S7,S10 |
| 5.94 | 6.59 | 319.1539 | -1.18 | C18H22O5 | Zearalenone | S2,S6,S5,S1,S9,S8,S7,S10 |
| 5.94 | 6.40 | 361.1645 | -0.81 | C20H24O6 | Lariciresinol | S2,S6,S5,S1,S9,S8,S7,S10 |
| 5.94 | 6.27 | 347.1854 | -0.42 | C20H26O5 | Gibberellin A24 | S2,S6,S5,S1,S9,S8,S7,S10 |
| 5.95 | 6.82 | 291.1591 | -0.64 | C17H22O4 | [FA oxo(4:0/4:0)] 9-oxo-12S-acetoxy-2,3,4,5-tetranor-7Z,10Z,14Z-prostatetrienaldehyde-cyclo[8,12] | S2,S6,S5,S1,S9,S8,S7,S10 |
| 5.95 | 6.39 | 467.1704 | 0.30 | C26H26O8 | Polystachin (flavone) | S2,S6,S5,S1,S9,S8,S7 |
| 5.95 | 6.92 | 237.1487 | -0.32 | C14H20O3 | 4-Heptyloxybenzoic acid | S2,S6,S5,S1,S9,S8,S7,S10 |
| 5.95 | 5.59 | 233.0808 | -1.28 | C13H12O4 | Goniothalenol | S2,S6,S5,S1,S9,S8,S7,S10 |
| 5.95 | 6.40 | 335.0402 | 0.68 | C15H10O9 | 3,5,6,7,2',3',4'-Heptahydroxyflavone | S7 |
| 5.95 | 6.90 | 724.5338 | -3.11 | C41H73NO9 | [PR] bacteriohopane-31,32,33,34-tetrol-35-cyclitol | S2,S6,S5,S1,S9,S7 |
| 5.95 | 6.40 | 825.5841 | -1.52 | C43H85O12P | PI(O-20:0/14:0) | S6,S5,S1,S7,S10 |
| 5.96 | 6.39 | 287.0914 | -0.87 | C16H14O5 | [Fv] Licodione 2'-methyl ether | S2,S6,S5,S1,S9,S8,S7,S10 |
| 5.96 | 6.91 | 639.4992 | 1.09 | C41H66O5 | DG(38:7) | S2,S6,S5,S1,S9,S8,S7,S10 |
| 5.96 | 6.36 | 235.0965 | -0.91 | C13H14O4 | 1'-Acetoxychavicol acetate | S2,S6,S5,S1,S9,S8,S7,S10 |
| 5.96 | 6.09 | 309.1696 | -0.79 | C17H24O5 | Inulicin | S2,S6,S5,S1,S9,S8,S7,S10 |
| 5.96 | 6.42 | 775.5834 | -2.05 | C43H83O9P | PG(O-20:0/17:2(9Z,12Z)) | S2,S6,S5,S1,S9,S8,S7,S10 |
| 5.96 | 5.82 | 249.1487 | -0.35 | C15H20O3 | [PR] 1,2-Dihydrosantonin | S2,S6,S5,S1,S9,S8,S7,S10 |
| 5.96 | 5.89 | 131.0339 | -1.29 | C5H6O4 | Mesaconate | S2 |
| 5.96 | 6.43 | 439.1749 | -1.00 | C25H26O7 | [Fv hydroxy,hydroxy,methyl,dimethyl(4:2/9:1)] 5,4-Dihydroxy-8-(1-hydroxy-2,3-epoxy-3-methylbutyl)-6'',6''-dimethylpyrano[2'',3'':7,6]flavanone | S2,S6,S5,S1,S9,S8,S7,S10 |
| 5.96 | 5.93 | 293.1749 | -0.18 | C17H24O4 | Trichodermin | S2,S6,S5,S1,S9,S8,S7,S10 |
| 5.96 | 6.55 | 223.0964 | -1.18 | C12H14O4 | [FA (12:4/2:0)] 2E,4E,8E,10E-Dodecatetraenedioic acid | S2,S6,S5,S1,S9,S8,S7,S10 |
| 5.96 | 6.43 | 437.2173 | 0.13 | C23H32O8 | Polhovolide | S2,S6,S5,S1,S9,S8,S7 |
| 5.97 | 6.43 | 401.1232 | -0.33 | C21H20O8 | alpha-Peltatin | S2,S6,S5,S1,S9,S8,S7 |
| 5.97 | 6.60 | 253.1435 | -0.78 | C14H20O4 | ubiquinol-1 | S2,S6,S5,S1,S9,S8,S7,S10 |
| 5.97 | 5.61 | 208.0968 | -1.13 | C11H13NO3 | N-Acetyl-L-phenylalanine | S2,S1,S9 |
| 5.97 | 5.82 | 363.1801 | -0.90 | C20H26O6 | Gibberellin A19 | S2,S6,S5,S1,S9,S8,S7,S10 |
| 5.97 | 6.31 | 331.1175 | -0.88 | C18H18O6 | [Fv Hydroxy,trimethoxy(9:1)] 4'-Hydroxy-5,6,7-trimethoxyflavanone | S2,S6,S5,S1,S9,S8,S7,S10 |
| 5.98 | 4.77 | 349.1641 | -1.94 | C19H24O6 | Gibberellin A1 | S2,S6,S5,S1,S9,S8,S7,S10 |
| 5.98 | 6.95 | 681.5438 | -2.52 | C44H72O5 | DG(19:1(9Z)/22:6(4Z,7Z,10Z,13Z,16Z,19Z)/0:0)[iso2] | S2,S6,S5,S1,S8,S7 |
| 5.98 | 5.89 | 263.1279 | -0.60 | C15H18O4 | [PR] Parthenin | S2,S6,S5,S1,S9,S8,S7,S10 |
| 5.98 | 6.24 | 256.1340 | 2.25 | C16H17NO2 | 6-(1,2,3,4-Tetrahydro-6-methoxy-2-naphthyl)-2(1H)-pyridone | S2,S6,S5,S1,S9,S8,S7 |
| 5.98 | 6.53 | 244.2272 | -0.68 | C14H29NO2 | [FA amino(14:0)] 2-amino-tetradecanoic acid | S2,S5,S1,S9,S8,S7,S10 |
| 5.98 | 4.47 | 744.5910 | 0.77 | C42H82NO7P | 1-Hexadecanoyl-2-(9Z-octadecenoyl)-sn-glycero-3-phosphonocholine | S2,S6,S5,S1,S9,S8,S7,S10 |
| 5.98 | 5.76 | 319.0452 | 0.39 | C15H10O8 | Myricetin | S2,S6,S8,S7 |
| 5.98 | 6.39 | 299.0909 | -2.32 | C17H14O5 | Afrormosin | S2,S6,S5,S1,S9,S8,S7,S10 |
| 5.99 | 4.47 | 788.6150 | -2.01 | C44H86NO8P | PC(36:1) | S2,S6,S5,S1,S9,S8,S7,S10 |
| 5.99 | 6.89 | 213.1479 | -3.82 | C12H20O3 | [FA oxo(12:1)] 12-oxo-10E-dodecenoic acid | S2,S6,S5,S1,S9,S8,S7,S10 |
| 5.99 | 7.05 | 367.2113 | -1.17 | C20H30O6 | [FA hydroxy(20:4/2:0)] 5S,12R-dihydroxy-6Z,8E,10E,14Z-eicosatetraene-1,20-dioic acid | S2,S6,S5,S1,S9,S8,S7,S10 |
| 5.99 | 6.71 | 343.1174 | -1.16 | C19H18O6 | [Fv] Tinosporinone | S2,S6,S5,S1,S9,S8,S7,S10 |
| 5.99 | 5.56 | 281.1383 | -1.03 | C15H20O5 | Phaseic acid | S2,S6,S5,S1,S9,S8,S7,S10 |
| 6.00 | 6.89 | 341.1384 | -0.63 | C20H20O5 | 6-Prenylnaringenin | S2,S6,S5,S1,S9,S8,S7,S10 |
| 6.00 | 5.85 | 223.1330 | -0.54 | C13H18O3 | (+/-)-6-Hydroxy-3-oxo-alpha-ionone | S2,S6,S5,S1,S9,S7,S10 |
| 6.00 | 5.22 | 395.1703 | -0.02 | C20H26O8 | GA43 | S2,S6,S5,S1,S9,S8,S7,S10 |
| 6.00 | 5.28 | 307.1539 | -0.95 | C17H22O5 | Matricin | S2,S6,S5,S1,S9,S8,S7,S10 |
| 6.00 | 6.34 | 221.0811 | 0.31 | C12H12O4 | 2,6-Dioxo-6-phenylhexanoate | S2,S6,S5,S1,S9,S8,S7,S10 |
| 6.00 | 6.00 | 537.2116 | -0.99 | C30H32O9 | Schisantherin A | S2,S6,S5,S1,S9,S8,S7,S10 |
| 6.00 | 6.15 | 289.0710 | 0.53 | C15H12O6 | Eriodictyol | S2,S6,S5,S1,S9,S8,S7,S10 |
| 6.00 | 6.22 | 349.2010 | -0.45 | C20H28O5 | Gibberellin A53 | S2,S6,S5,S1,S9,S8,S7,S10 |
| 6.00 | 5.84 | 137.0599 | -0.49 | C8H8O2 | Phenylacetic acid | S2,S6,S5,S1,S9,S8,S7,S10 |
| 6.01 | 6.51 | 569.2015 | 2.41 | C27H32N6O4S2 | bistratamide B | S2,S6,S5,S1,S9,S8,S7 |
| 6.01 | 6.51 | 543.1872 | 1.57 | C28H30O11 | Ikarisoside D | S2,S6,S5,S1,S9,S8,S7,S10 |
| 6.01 | 6.35 | 209.1173 | -0.52 | C12H16O3 | Benzyl (2R,3S)-2-methyl-3-hydroxybutanoate | S2,S6,S5,S1,S9,S8,S7,S10 |
| 6.01 | 6.81 | 482.4574 | 0.79 | C30H59NO3 | [SP (12:0)] N-(dodecanoyl)-sphing-4-enine | S2,S6,S5,S1,S9,S10 |
| 6.01 | 5.79 | 247.0966 | -0.55 | C14H14O4 | Columbianetin | S2,S6,S5,S1,S9,S8,S7,S10 |
| 6.01 | 6.82 | 275.0915 | -0.57 | C15H14O5 | [Fv] Phloretin | S2,S6,S5,S9,S8,S7,S10 |
| 6.01 | 6.02 | 541.1708 | 0.30 | C28H28O11 | Cleistanthin A | S2,S6,S5,S1,S9,S8,S7,S10 |
| 6.01 | 4.27 | 379.1748 | -1.39 | C20H26O7 | Chaparrinone | S2,S6,S5,S1,S9,S8,S7,S10 |
| 6.01 | 5.36 | 153.0548 | -0.61 | C8H8O3 | 3,4-Dihydroxyphenylacetaldehyde | S2,S6,S5,S1,S9,S8,S7,S10 |
| 6.01 | 6.52 | 587.2491 | 0.26 | C31H38O11 | Baccatin III | S2,S6,S5,S8 |
| 6.01 | 6.52 | 527.1905 | -1.73 | C28H30O10 | Pilosanol B | S2,S6,S5,S1,S9,S8,S7,S10 |
| 6.02 | 4.90 | 113.0236 | 0.20 | C5H4O3 | 2-Furoate | S2,S6,S5,S1,S9,S8,S7,S10 |
| 6.02 | 7.30 | 399.3620 | -0.92 | C28H46O | [ST methy] 24-methylene-cholest-8-en-3beta-ol | S2,S6,S1,S8,S7,S10 |
| 6.02 | 3.54 | 381.1909 | -0.19 | C20H28O7 | Chaparrin | S2,S6,S5,S1,S9,S8,S7,S10 |
| 6.02 | 6.85 | 193.1225 | -0.19 | C12H16O2 | [FA (12:4)] 2E,4E,8Z,10E-dodecatetraenoic acid | S2,S6,S5,S1,S9,S8,S7,S10 |
| 6.02 | 6.61 | 345.1334 | -0.40 | C19H20O6 | Deoxyelephantopin | S2,S6,S5,S1,S9,S8,S7,S10 |
| 6.02 | 6.99 | 244.1908 | -0.43 | C13H25NO3 | N-Undecanoylglycine | S2,S6,S5,S1,S9,S8,S7,S10 |
| 6.02 | 6.55 | 777.5990 | -2.07 | C43H85O9P | PG(O-18:0/19:1(9Z)) | S2,S6,S5,S1,S9,S8,S7,S10 |
| 6.03 | 3.41 | 124.0759 | -0.01 | C7H9NO | 2-amino-m-cresol | S2,S6,S5,S1,S9,S8,S7,S10 |
| 6.03 | 6.53 | 211.1331 | -0.18 | C12H18O3 | (+)-7-Isojasmonic acid | S2,S6,S5,S1,S9,S8,S10 |
| 6.03 | 5.83 | 207.0653 | -0.70 | C11H10O4 | Scoparone | S2,S6,S5,S1,S9,S8,S7,S10 |
| 6.03 | 6.38 | 144.1384 | -0.71 | C8H17NO | valpromide | S2,S6,S5,S1,S9,S8,S7,S10 |
| 6.03 | 5.03 | 279.1226 | -1.25 | C15H18O5 | Artecanin | S2,S6,S5,S1,S9,S8,S7,S10 |
| 6.03 | 5.79 | 323.1488 | -1.11 | C17H22O6 | Tetraneurin A | S2,S6,S5,S1,S9,S8,S7,S10 |
| 6.04 | 6.43 | 219.1017 | -0.53 | C13H14O3 | 6-Hydroxytremetone | S2,S6,S5,S1,S9,S8,S7,S10 |
| 6.04 | 4.85 | 347.1486 | -1.63 | C19H22O6 | Gibberellin A3 | S2,S6,S5,S1,S9,S8,S7,S10 |
| 6.04 | 6.36 | 193.0860 | -0.69 | C11H12O3 | Carpacin | S2,S6,S5,S1,S9,S8,S7,S10 |
| 6.04 | 6.34 | 261.0401 | 1.96 | C13H8O6 | Demethylbellidifolin | S2,S6,S5,S1,S9,S8,S7 |
| 6.04 | 5.95 | 339.1437 | -1.20 | C17H22O7 | 1-Peroxyferolide | S2,S6,S5,S1,S9,S8,S7,S10 |
| 6.04 | 6.58 | 319.1171 | -2.27 | C17H18O6 | [Fv] Protofarrerol | S2,S6,S5,S1,S9,S8,S7,S10 |
| 6.04 | 6.06 | 227.1274 | -2.53 | C12H18O4 | [FA oxo,hydroxy(5:1/5:0)] (1S,2R)-3-oxo-2-(5'-hydroxy-2'Z-pentenyl)-cyclopentaneacetic acid | S2,S6,S5,S1,S9,S8,S7,S10 |
| 6.04 | 5.50 | 177.1122 | -0.73 | C8H16O4 | [FA hydroxy(8:0)] 6,8-dihydroxy-octanoic acid | S6,S5,S1,S9,S8,S10 |
| 6.04 | 6.70 | 271.0965 | -0.77 | C16H14O4 | [Fv] Isoliquiritigenin 2'-methy ether | S2,S6,S5,S1,S9,S8,S7,S10 |
| 6.04 | 6.94 | 371.1126 | -0.39 | C20H18O7 | [Fv Hydroxy,dimethoxy,methy] 2'-Hydroxy-5',6'-dimethoxy-3,4-methylenedioxyfurano[2'',3'':4',3'] dihydrochalcone | S8,S7 |
| 6.04 | 6.86 | 257.1175 | 0.13 | C16H16O3 | Pterostilbene | S2,S6,S5,S1,S9,S8,S7,S10 |
| 6.05 | 4.59 | 816.6464 | -1.81 | C46H90NO8P | PC(38:1) | S2,S6,S5,S1,S9,S8,S7,S10 |
| 6.05 | 7.10 | 384.3115 | 1.22 | C22H41NO4 | N-oleoyl threonine | S2,S6,S5,S1,S9,S8,S7,S10 |
| 6.05 | 3.71 | 365.1588 | -2.52 | C19H24O7 | [PR] Gibberellin A8 | S2,S6,S5,S1,S9,S8,S7,S10 |
| 6.05 | 6.82 | 400.2330 | -0.38 | C20H33NO7 | Candoxatrilat | S2,S6,S5,S1,S9,S8,S7,S10 |
| 6.05 | 6.61 | 409.1859 | -0.03 | C21H28O8 | Vernoflexuoside | S2,S6,S5,S1,S9,S8,S7,S10 |
| 6.05 | 7.11 | 423.2005 | -2.51 | C22H30O8 | Valtratum | S2,S6,S5,S1,S9,S8,S7,S10 |
| 6.06 | 6.91 | 728.5964 | 1.25 | C42H82NO6P | [SP (24:0)] N-(15Z-tetracosenoyl)-sphing-4-enine-1-phosphate | S2,S6,S5,S1,S9,S8,S7,S10 |
| 6.06 | 6.61 | 338.2690 | -3.07 | C38H75O7P | PA(O-16:0/19:1(9Z)) | S2,S6,S5,S1,S9,S8,S7,S10 |
| 6.06 | 5.13 | 140.0343 | -0.89 | C6H5NO3 | 6-Hydroxynicotinate | S2,S6,S5,S1,S9,S8,S7 |
| 6.06 | 6.62 | 727.5280 | 0.69 | C41H75O8P | PA(38:3) | S2,S6,S5,S1,S9,S8,S7,S10 |
| 6.06 | 5.98 | 291.1226 | -1.22 | C16H18O5 | [PK] 5-O-Methylvisamminol | S2,S6,S5,S1,S9,S8,S7,S10 |
| 6.06 | 6.62 | 799.5494 | 1.03 | C44H79O10P | PG(38:4) | S2,S6,S5,S1,S9,S8,S7,S10 |
| 6.06 | 6.55 | 202.1437 | -1.20 | C10H19NO3 | Capryloylglycine | S2,S5,S1,S9 |
| 6.06 | 6.90 | 293.0302 | -0.39 | C14H12O3S2 | 5-(3-Hydroxy-4-acetoxybut-1-ynyl)-2,2'-bithiophene | S2,S6,S5,S1,S8,S7 |
| 6.06 | 7.53 | 269.2840 | -0.40 | C18H36O | Octadecanal | S2,S6,S5,S1,S9,S8 |
| 6.07 | 6.94 | 357.1327 | -2.20 | C20H20O6 | Kievitone | S2,S6,S5,S1,S9,S8,S7,S10 |
| 6.07 | 7.39 | 339.2529 | -0.99 | C20H34O4 | [FA hydroxy(20:3)] 8,9-dihydroxy-5Z,11Z,14Z-eicosatrienoic acid | S2,S6,S5,S1,S9,S8,S7,S10 |
| 6.07 | 7.14 | 409.1487 | -2.06 | C20H24O9 | Ginkgolide A | S2,S6,S5,S1,S9,S8,S7,S10 |
| 6.08 | 5.51 | 325.1643 | -1.58 | C17H24O6 | Tetraneurin E | S2,S6,S5,S1,S9,S8,S7,S10 |
| 6.08 | 6.49 | 251.1276 | -1.53 | C14H18O4 | ubiquinone-1 | S2,S6,S5,S1,S9,S8,S7,S10 |
| 6.08 | 6.67 | 359.1124 | -0.90 | C19H18O7 | [Fv Methyl,trimethoxy,hydrox] 3,4-Methylenedioxy-2',4',6'-trimethoxy-beta-hydroxychalcone | S2,S6,S5,S1,S9,S8,S7,S10 |
| 6.09 | 6.61 | 327.1428 | -4.00 | C16H22O7 | [FA hydroxy,dioxo(4:0/2:0)] 11R-hydroxy-9,15-dioxo-2,3,4,5-tetranor-prostan-1,20-dioic acid | S2,S6,S5,S1,S9,S8,S10 |
| 6.09 | 6.21 | 201.0374 | 1.40 | C12H8OS | dibenzothiophene-5-oxide | S2,S8,S7 |
| 6.09 | 6.78 | 375.1439 | -0.31 | C20H22O7 | Kievitone hydrate | S2,S6,S5,S1,S9,S8,S7,S10 |
| 6.10 | 3.95 | 295.1173 | -1.84 | C15H18O6 | Tutin | S2,S6,S5,S1,S9,S8,S7,S10 |
| 6.10 | 6.69 | 487.1965 | 0.09 | C26H30O9 | [Fv Trihydrox] 2',4',4-Trihydroxy-3'-prenylchalcone 4'-O-glucoside | S2,S6,S5,S1,S9,S8,S7,S10 |
| 6.10 | 5.42 | 87.0446 | 3.94 | C4H6O2 | 2-Butenoate | S2,S6,S5,S1,S9,S8,S7,S10 |
| 6.10 | 5.83 | 321.1329 | -1.88 | C17H20O6 | PR-toxin | S2,S6,S5,S1,S9,S8,S7,S10 |
| 6.11 | 5.97 | 255.1227 | -1.06 | C13H18O5 | [FA methyl,hydroxy,oxo(5:2/4:0)] methyl 4-[2-(2-formyl-vinyl)-3-hydroxy-5-oxo-cyclopentyl]-butanoate | S2,S6,S5,S1,S9,S8,S7,S10 |
| 6.11 | 6.72 | 499.1609 | 1.57 | C26H26O10 | Phylloflavan | S2,S6,S5,S1,S9,S8,S7 |
| 6.12 | 5.83 | 199.0598 | -2.47 | C9H10O5 | Syringic acid | S2,S6,S5,S1,S9,S8,S7 |
| 6.12 | 7.14 | 315.1229 | -0.02 | C18H18O5 | [Fv Hydroxy,methox] 2'-Hydroxy-3,4,5-methoxychalcone | S2,S6,S5,S1,S9,S8,S7,S10 |
| 6.13 | 6.16 | 261.0755 | -1.98 | C14H12O5 | Khellin | S2,S6,S5,S1,S9,S8,S7,S10 |
| 6.13 | 7.03 | 225.1122 | -0.70 | C12H16O4 | Aspidinol | S2,S6,S5,S1,S9,S8,S7,S10 |
| 6.13 | 6.26 | 961.5859 | 1.50 | C48H84N2O17 | Megalomicin C1 | S7,S10 |
| 6.13 | 6.27 | 243.0497 | -3.26 | C6H13NO7P | [PE] 2-acyl-sn-glycero-3-phosphoethanolamine | S2,S6,S9,S8 |
| 6.13 | 5.76 | 139.0391 | -0.84 | C7H6O3 | 4-Hydroxybenzoate | S2,S6,S5,S1,S9,S8,S7,S10 |
| 6.14 | 6.58 | 329.1022 | -0.08 | C18H16O6 | Betagarin | S2,S6,S5,S1,S9,S8,S7,S10 |
| 6.14 | 6.77 | 529.1706 | -0.16 | C27H28O11 | Tremulacin | S2,S6,S5,S1,S9,S8,S7 |
| 6.14 | 5.06 | 227.0549 | -1.72 | C10H10O6 | Prephenate | S2,S6,S5,S1,S9,S8,S7 |
| 6.15 | 6.11 | 118.0655 | 1.04 | C8H7N | Indole | S2,S6,S5,S1,S9 |
| 6.15 | 6.80 | 389.0871 | 0.53 | C19H16O9 | Gossypetin 7,4'-dimethyl ether 8-acetate | S2,S6,S5,S1,S9,S8,S7 |
| 6.15 | 6.30 | 483.1654 | -3.36 | C27H28FN2OClS | SSR 125543 | S2,S6,S5,S1,S9,S8,S7 |
| 6.15 | 5.63 | 216.1960 | -0.12 | C12H25NO2 | [FA amino(12:0)] 12-amino-dodecanoic acid | S2,S6,S5,S1,S9,S8,S7,S10 |
| 6.16 | 6.48 | 188.1282 | -0.91 | C9H17NO3 | N-Heptanoylglycine | S2,S6,S5,S1,S9 |
| 6.17 | 4.92 | 185.0446 | -0.50 | C8H8O5 | 3,4-Dihydroxymandelate | S2,S6,S5,S1,S8,S7 |
| 6.17 | 6.21 | 130.1229 | 0.00 | C7H15NO | N-Methylhexanamide | S2,S6,S5,S1,S9 |
| 6.17 | 6.91 | 357.2055 | -2.14 | C22H28O4 | (17Z)-3,11-Dioxopregna-4,17(20)-dien-21-oic acid methyl ester | S2,S6,S5,S1,S9,S8,S7,S10 |
| 6.17 | 7.14 | 812.6945 | -3.84 | C48H93NO8 | [SP (24:0)] N-(tetracosanoyl)-1-beta-glucosyl-sphing-4-enine | S2,S6,S5,S1,S9,S8,S7,S10 |
| 6.17 | 6.85 | 349.1281 | -0.90 | C18H20O7 | Machaerol C | S2,S6,S5,S1,S9,S8,S7,S10 |
| 6.17 | 6.85 | 527.1559 | 1.66 | C27H26O11 | Viscutin 1 | S2,S6,S5,S9,S8,S7 |
| 6.19 | 4.98 | 313.1273 | -3.49 | C15H20O7 | 4-Hydroxycinnamyl alcohol 4-D-glucoside | S2,S6,S5,S1,S9,S8,S7 |
| 6.19 | 6.38 | 854.5877 | -3.57 | C47H84NO10P | PS(19:0/22:4(7Z,10Z,13Z,16Z)) | S2,S6,S5,S1,S9,S8,S7,S10 |
| 6.19 | 5.27 | 174.0916 | 0.02 | C11H11NO | 1,3-Dimethyl-8-isoquinolinol | S2,S6,S1,S9,S8,S7 |
| 6.20 | 6.89 | 515.1916 | 0.33 | C27H30O10 | Icariside II | S2,S6,S5,S1,S9,S8,S7,S10 |
| 6.20 | 4.66 | 221.0446 | -0.41 | C11H8O5 | Purpurogallin | S2,S6,S5,S1,S9,S8,S7,S10 |
| 6.21 | 6.46 | 359.0766 | 0.58 | C18H14O8 | versiconal | S2,S6,S5,S9,S8,S7 |
| 6.22 | 6.90 | 375.1074 | -0.68 | C19H18O8 | Chrysosplenetin | S2,S6,S5,S1,S9,S8,S7,S10 |
| 6.22 | 6.59 | 275.0549 | -1.41 | C14H10O6 | Athyriol | S2,S6,S5,S1,S9,S8,S7 |
| 6.22 | 6.36 | 391.1386 | -0.98 | C20H22O8 | Piceid | S2,S6,S5,S1,S9,S8,S7,S10 |
| 6.22 | 6.94 | 713.5119 | 0.12 | C40H73O8P | PA(15:1(9Z)/22:2(13Z,16Z)) | S2,S6,S5,S1,S9,S8,S7,S10 |
| 6.22 | 4.77 | 146.0813 | -0.92 | C6H11NO3 | 4-Acetamidobutanoate | S2,S9 |
| 6.22 | 6.44 | 681.6150 | 1.20 | C41H80N2O5 | ornithine lipid | S2,S6,S5,S1,S8 |
| 6.23 | 5.60 | 103.0394 | 2.17 | C4H6O3 | 2-Oxobutanoate | S2,S6,S5,S1,S9 |
| 6.24 | 6.97 | 363.1073 | -1.09 | C18H18O8 | [Fv Trihydroxy,trimethoxy(9:1)] 5,7,3'-Trihydroxy-6,4',5'-trimethoxyflavanone | S2,S6,S5,S1,S9,S8,S7 |
| 6.25 | 6.69 | 232.0976 | 2.21 | C13H13NO3 | Benzoyldehydro-2,3-dihydroxy-benzone | S2,S6,S5,S1,S9 |
| 6.25 | 6.99 | 310.2377 | -3.20 | C34H67O7P | PA(O-16:0/15:1(9Z)) | S2,S6,S5,S1,S9,S8,S7,S10 |
| 6.25 | 6.30 | 160.0760 | 0.73 | C10H9NO | Indole-3-acetaldehyde | S2,S6,S5,S1,S9,S8,S7 |
| 6.29 | 6.67 | 347.1130 | 0.56 | C18H18O7 | Reduced-(S)-usnate | S2,S6,S5,S1,S9,S8,S7,S10 |
| 6.32 | 7.53 | 319.0811 | -1.25 | C16H14O7 | [PK] Lecanoric acid | S2,S6,S5,S1,S9,S8,S7,S10 |
| 6.33 | 6.86 | 293.1493 | -1.73 | C15H20N2O4 | Phenylbutyrylglutamine | S2 |
| 6.35 | 7.20 | 773.5339 | 1.20 | C42H77O10P | PG(36:3) | S2,S6,S5,S1,S9,S8,S7,S10 |
| 6.37 | 7.68 | 229.1429 | -3.18 | C12H20O4 | Traumatic acid | S2,S6,S5,S1,S9,S8,S7,S10 |
| 6.38 | 7.26 | 581.1674 | 3.09 | C30H28O12 | [Fv hydroxy(4:0)] 4,2',3',4'-Tetrahydroxychalcone 4'-O-(2''-O-p-coumaroyl)glucoside | S2,S6,S1,S9,S8,S7 |
| 6.39 | 7.29 | 729.4696 | -0.97 | C39H69O10P | PG(13:0/20:4(5Z,8Z,11Z,14Z)) | S2,S5,S1,S9,S8,S7,S10 |
| 6.40 | 7.29 | 731.5583 | -0.61 | C41H79O8P | PA(38:1) | S2,S6,S5,S1,S9,S8,S7,S10 |
| 6.40 | 6.96 | 127.1119 | -0.49 | C8H14O | Sulcatone | S6,S5,S9,S8,S7,S10 |
| 6.41 | 7.31 | 703.5284 | 1.31 | C39H75O8P | PA(36:1) | S2,S6,S5,S1,S9,S8,S7,S10 |
| 6.41 | 7.32 | 729.5439 | 1.02 | C41H77O8P | PA(38:2) | S2,S6,S5,S1,S9,S8,S7,S10 |
| 6.41 | 6.79 | 131.0706 | 0.66 | C6H10O3 | (S)-3-Methyl-2-oxopentanoic acid | S2,S6,S5,S1,S9,S8,S7,S10 |
| 6.43 | 7.86 | 503.4099 | 0.31 | C32H54O4 | [ST hydroxy(2:0)] (5Z,7E)-(1R,2R,3R)-2-(5-hydroxypentoxy)-9,10-seco-5,7,10(19)-cholestatriene-1,3-diol | S2,S5,S1,S9,S8,S7,S10 |
| 6.43 | 5.22 | 396.1713 | 1.18 | C25H21N3O2 | Oxidized Renilla luciferin | S2,S6,S5,S1,S9,S8,S7,S10 |
| 6.44 | 5.01 | 148.0606 | -0.11 | C5H9NO4 | N-hydroxy-N-isopropyloxamate | S2 |
| 6.45 | 7.36 | 265.1070 | -0.99 | C14H16O5 | 1'-Acetoxyeugenol acetate | S2,S6,S5,S1,S9,S8,S7,S10 |
| 6.45 | 7.91 | 411.3626 | 0.44 | C29H46O | [ST dimethyl(3:0)] 4,4-dimethylcholesta-8,11,24-trien-3beta-ol | S2,S6,S5,S1,S9,S8,S7,S10 |
| 6.47 | 6.95 | 828.5765 | 1.70 | C45H82NO10P | PS(17:1(9Z)/22:2(13Z,16Z)) | S2,S6,S5,S1,S8,S7,S10 |
| 6.48 | 7.46 | 417.1188 | 1.23 | C21H20O9 | Chrysophanol 8-O-beta-D-glucoside | S2,S6,S5,S1,S9,S8,S7 |
| 6.50 | 6.99 | 636.4608 | 1.12 | C33H66NO8P | PE(28:0) | S2,S6,S5,S1,S9,S8,S7,S10 |
| 6.50 | 3.61 | 298.1146 | -0.80 | C11H15N5O5 | 2-Methylguanosine | S2,S6,S5,S1,S9,S8,S7,S10 |
| 6.52 | 5.42 | 186.1126 | -0.33 | C9H15NO3 | 8-keto-7-aminoperlagonate | S2,S6,S5,S1,S9 |
| 6.54 | 8.08 | 547.4510 | -0.38 | C38H58O2 | [PR] all-trans-retinyl linolate | S2,S6,S5,S1,S9,S8,S7,S10 |
| 6.55 | 7.83 | 323.0917 | 0.35 | C19H14O5 | [Fv] Ovalitenin C | S2,S6,S5,S1,S9,S8,S7,S10 |
| 6.55 | 6.41 | 393.1544 | -0.50 | C20H24O8 | Eupatoroxin | S2,S6,S5,S1,S9,S8,S7,S10 |
| 6.58 | 6.52 | 307.1172 | -1.96 | C16H18O6 | Cimifugin | S2,S6,S5,S1,S9,S8,S7,S10 |
| 6.58 | 8.53 | 301.3108 | 1.59 | C19H40O2 | [FA methyl(18:0/2:0)] 2-methyloctadecane-1,2-diol | S7,S10 |
| 6.61 | 8.21 | 273.2576 | -1.19 | C20H32 | Taxa-4(5),11(12)-diene | S5,S1,S9,S8,S7,S10 |
| 6.62 | 7.73 | 925.6155 | -1.30 | C51H89O12P | PI(O-20:0/22:6(4Z,7Z,10Z,13Z,16Z,19Z)) | S8,S7,S10 |
| 6.64 | 6.78 | 99.0444 | 1.54 | C5H6O2 | [FA (5:2)] 2,4-pentadienoic acid | S2,S6,S5,S1,S9,S8,S7,S10 |
| 6.68 | 7.86 | 705.5443 | 1.66 | C39H77O8P | PA(36:0) | S2,S6,S5,S1,S9,S8,S7,S10 |
| 6.68 | 6.31 | 185.0805 | -3.09 | C9H12O4 | 3-Methoxy-4-hydroxyphenylethyleneglycol | S2,S6,S5,S1,S9,S8,S7,S10 |
| 6.74 | 8.45 | 389.3048 | -1.27 | C25H40O3 | [ST (2:0)] (5Z,7E)-(1S,3R)-21-nor-20-oxa-9,10-seco-5,7,10(19)-cholestatriene-1,3-diol | S2,S5,S1,S9,S8,S7,S10 |
| 6.75 | 7.21 | 135.0442 | -0.56 | C8H6O2 | [FA (8:1/2:0)] 6E-Octene-2,4-diynoic acid | S2,S6,S5,S1,S9,S8,S7,S10 |
| 6.76 | 8.51 | 413.3783 | 0.68 | C29H48O | [ST dimethyl(2:0)] 4,4-dimethyl-cholesta-8,24-dien-3beta-ol | S2,S6,S5,S1,S9,S8,S7,S10 |
| 6.76 | 8.01 | 771.5182 | 1.18 | C42H75O10P | [PG (16:0/20:4)] 1-hexadecanoyl-2-(5Z,8Z,11Z,14Z-eicosatetraenoyl)-sn-glycero-3-phospho-(1'-sn-glycerol) | S2,S6,S5,S1,S9,S8,S7,S10 |
| 6.76 | 8.78 | 379.1906 | -0.14 | C24H26O4 | Albafuran A | S2,S6,S5,S1,S9,S8,S7,S10 |
| 6.77 | 7.84 | 303.0866 | 0.15 | C16H14O6 | Hesperetin | S2,S6,S5,S1,S9,S8,S7,S10 |
| 6.77 | 8.04 | 603.3669 | -2.24 | C38H50O6 | Garcinol | S2,S6,S5,S9,S8,S7,S10 |
| 6.77 | 8.99 | 341.3060 | 2.23 | C21H40O3 | [FA oxo(21:0)] 2-oxo-heneicosanoic acid | S2,S6,S5,S1,S9,S8,S7,S10 |
| 6.77 | 8.91 | 305.2465 | -3.88 | C20H32O2 | [FA (20:4)] 5Z,8Z,11Z,14Z-eicosatetraenoic acid | S2,S6,S5,S1,S9,S8,S7,S10 |
| 6.79 | 7.58 | 991.6197 | 0.73 | C55H92O11P2 | decaprenyl-phosphoryl-&beta;-D-5-phosphoribose | S5,S7,S10 |
| 6.80 | 8.09 | 442.1700 | 3.32 | C24H27NO5S | troglitazone | S2,S6,S5,S1,S9,S8,S7 |
| 6.80 | 8.53 | 359.2581 | -0.69 | C23H34O3 | 16-Butyl-3-methoxy-estra-1,3,5(10)-triene-16beta,17beta-diol | S2,S6,S5,S1,S9,S8,S7,S10 |
| 6.80 | 8.10 | 417.2639 | 0.26 | C25H36O5 | 16alpha,17beta-Dihydroxyandrost-4-en-3-one dipropionate | S2,S6,S5,S1,S9,S8,S7,S10 |
| 6.80 | 8.61 | 459.3487 | 3.38 | C29H46O4 | [ST (2:0)] (5Z,7E)-(1S,3R)-18-acetoxy-9,10-seco-5,7,10(19)-cholestatriene-1,3-diol | S2,S6,S5,S1,S9,S8,S7,S10 |
| 6.80 | 8.91 | 321.2422 | -1.26 | C20H32O3 | [FA hydroxy(20:4)] 5S-hydroxy-6E,8Z,11Z,14Z-eicosatetraenoic acid | S2,S6,S5,S1,S9,S8,S7,S10 |
| 6.81 | 8.61 | 475.3416 | -0.89 | C29H46O5 | [ST (3:0)] (5Z,7E)-(1S,3R)-18-acetoxy-9,10-seco-5,7,10(19)-cholestatriene-1,3,25-triol | S2,S6,S5,S1,S9,S8,S7,S10 |
| 6.81 | 8.12 | 483.2739 | 3.99 | C22H43O9P | PG(16:1(9Z)/0:0) | S2,S6,S5,S1,S9,S8,S7,S10 |
| 6.81 | 8.63 | 457.2947 | -0.85 | C28H40O5 | Minabeolide-7 | S2,S6,S5,S1,S9,S8,S7,S10 |
| 6.82 | 7.88 | 335.0764 | 0.23 | C16H14O8 | [Fv hydroxy,methoxy,methyl,trihydroxy(9:1)] (-)-2-[(3,5-Dihydroxy-4-methoxyphenyl)methyl]-2,4,6-trihydroxy-3(2H)-benzofuranone | S2,S6,S5,S1,S9,S8,S7 |
| 6.83 | 8.16 | 443.3511 | -2.58 | C29H46O3 | 4alpha-Methylzymosterol-4-carboxylate | S2,S6,S5,S1,S9,S8,S7,S10 |
| 6.83 | 7.43 | 187.0392 | -0.23 | C11H6O3 | Angelicin | S2,S6,S5,S1,S9,S8,S7 |
| 6.83 | 6.83 | 436.3294 | 2.12 | C23H41N5O3 | Philanthotoxin 343 | S5,S1,S9,S7,S10 |
| 6.83 | 8.66 | 573.4536 | 3.52 | C36H60O5 | DG(13:0/20:5(5Z,8Z,11Z,14Z,17Z)/0:0)[iso2] | S2,S6,S5,S1,S9,S8,S7,S10 |
| 6.84 | 6.90 | 180.0658 | 0.07 | C9H9NO3 | Hippurate | S2,S6,S5,S1,S9,S7,S10 |
| 6.84 | 8.17 | 335.2571 | -3.45 | C21H34O3 | 5-alpha-THDOC | S6,S5,S1,S9,S8,S7,S10 |
| 6.84 | 8.19 | 433.2591 | 1.00 | C25H36O6 | Glycinoeclepin A | S2,S6,S5,S1,S9,S8,S7,S10 |
| 6.84 | 8.13 | 137.1326 | -0.38 | C10H16 | [PR] (-)-Limonene | S2,S6,S5,S1,S9,S8,S7,S10 |
| 6.85 | 9.01 | 295.2272 | 0.78 | C18H30O3 | [FA (18:2)] 13-keto-9Z,11E-octadecadienoic acid | S2,S6,S5,S1,S9,S8,S7,S10 |
| 6.85 | 8.70 | 487.2692 | -0.19 | C28H38O7 | 15beta-Hydroxynicandrin B | S2,S6,S5,S1,S9,S8,S7,S10 |
| 6.85 | 8.47 | 395.2789 | -1.22 | C23H38O5 | Tylactone | S2,S6,S5,S1,S9,S8,S7,S10 |
| 6.85 | 8.70 | 687.4974 | -1.66 | C45H66O5 | [GL (20:5/22:6)] 1-(5Z,8Z,11Z,14Z,17Z-eicosapentaenoyl)-2-(4Z,7Z,10Z,13Z,16Z,19Z-docosahexaenoyl)-sn-glycerol | S2,S6,S5,S1,S9,S8,S7,S10 |
| 6.86 | 8.21 | 407.2796 | 0.37 | C24H38O5 | 3alpha,12alpha-Dihydroxy-7-oxo-5beta-cholanate | S2,S6,S5,S1,S9,S8,S7,S10 |
| 6.86 | 7.88 | 151.0755 | -0.74 | C9H10O2 | Phenylpropanoate | S2,S6,S5,S1,S9,S8,S7,S10 |
| 6.86 | 8.23 | 675.4969 | 1.12 | C37H71O8P | PA(34:1) | S2,S6,S5,S1,S9,S8,S7,S10 |
| 6.86 | 8.73 | 463.3413 | -1.49 | C28H46O5 | Stoloniferone F | S6,S5,S1,S9,S8,S7,S10 |
| 6.87 | 7.78 | 391.2109 | -2.10 | C22H30O6 | Neoquassin | S2,S6,S5,S1,S9,S8,S7,S10 |
| 6.87 | 8.46 | 328.3216 | 1.18 | C20H41NO2 | N,N-Dimethylsphing-4-enine | S2,S5,S1,S9 |
| 6.88 | 7.43 | 179.0341 | -0.27 | C9H6O4 | 7,8-Dihydroxycoumarin | S2,S6,S1,S9,S8,S7,S10 |
| 6.88 | 8.17 | 219.1745 | -0.13 | C15H22O | albaflavenone | S2,S6,S5,S1,S9,S8,S7,S10 |
| 6.89 | 8.28 | 503.3355 | -2.94 | C30H46O6 | medicagenate | S2,S6,S5,S1,S9,S8,S7,S10 |
| 6.89 | 7.72 | 381.2627 | -2.72 | C22H36O5 | [ST trihydrox] 24-dinor-3alpha,7alpha,12alpha-trihydroxy-5beta-cholan-22-oic acid | S2,S6,S5,S1,S9,S8,S7,S10 |
| 6.89 | 7.51 | 209.0444 | -1.32 | C10H8O5 | Fraxetin | S2,S6,S5,S1,S9,S8,S7,S10 |
| 6.90 | 7.45 | 365.2322 | -0.69 | C21H32O5 | 11beta,17alpha,21-Trihydroxy-5beta-pregnane-3,20-dione | S2,S6,S5,S1,S9,S8,S7,S10 |
| 6.90 | 6.90 | 435.2386 | -0.16 | C17H34N6O5S | Glutathionylspermidine | S2,S6,S5,S1,S9,S8,S7,S10 |
| 6.90 | 9.01 | 309.2062 | -0.23 | C18H28O4 | 5-O-Methylembelin | S2,S6,S5,S1,S9,S8,S7,S10 |
| 6.90 | 8.30 | 423.1302 | 3.22 | C20H22O10 | [Fv hydroxy(5:0)] 2',4',3,4,alpha-Pentahydroxydihydrochalcone 3'-C-xyloside | S2,S6,S5,S1,S9,S8,S7 |
| 6.90 | 8.30 | 419.2788 | -1.57 | C25H38O5 | 3alpha,12alpha-Dihydroxy-5beta-pregnan-20-one diacetate | S6,S5,S1,S9,S8,S7,S10 |
| 6.90 | 6.29 | 293.1018 | -1.26 | C15H16O6 | [PR] (-)-Picrotoxinin | S2,S6,S5,S1,S9,S8,S7,S10 |
| 6.91 | 8.06 | 203.1069 | -0.06 | C13H14O2 | Tremetone | S2,S6,S5,S1,S9,S8,S7,S10 |
| 6.91 | 8.47 | 201.1120 | -1.72 | C10H16O4 | [FA (10:1/2:0)] 2E-Decenedioic acid | S2,S6,S5,S1,S8,S7,S10 |
| 6.91 | 8.33 | 653.3306 | -2.56 | C37H48O10 | Gnididilatin | S2,S6,S5,S9,S8,S7,S10 |
| 6.92 | 8.99 | 213.1848 | -1.69 | C13H24O2 | [FA methyl(12:1)] 2-methyl-2-dodecenoic acid | S2,S6,S5,S1,S9,S8,S7,S10 |
| 6.92 | 8.33 | 801.6337 | -4.07 | C46H89O8P | PA(21:0/22:1(11Z)) | S6,S5,S1,S9,S8,S7,S10 |
| 6.92 | 8.33 | 663.4230 | -0.52 | C34H63O10P | PG(14:1(9Z)/14:1(9Z)) | S8,S7,S10 |
| 6.92 | 8.79 | 237.1849 | -0.90 | C15H24O2 | Farnesoic acid | S2,S6,S5,S1,S9,S8,S7,S10 |
| 6.92 | 5.37 | 157.0498 | 0.14 | C7H8O4 | 2,3-Dihydro-2,3-dihydroxybenzoate | S2,S6,S5,S1,S9,S8,S7,S10 |
| 6.92 | 8.84 | 660.5679 | 0.50 | C40H73N3O4 | [PR] N-ornithinyl-35-aminobacteriohopane-32,33,34-triol | S2,S6,S5,S1,S9,S8,S7,S10 |
| 6.92 | 8.51 | 187.1330 | -0.77 | C10H18O3 | 10-Oxodecanoate | S2,S5,S1,S9,S8,S7,S10 |
| 6.92 | 8.46 | 171.1381 | -0.40 | C10H18O2 | [FA dimethyl(8:1)] 3,7-dimethyl-6-octenoic acid | S2,S6,S5,S1,S9,S8,S7,S10 |
| 6.93 | 8.35 | 471.2739 | -0.96 | C28H38O6 | Withanolide D | S2,S6,S5,S1,S9,S8,S7,S10 |
| 6.93 | 8.36 | 377.1233 | -0.15 | C19H20O8 | [Fv hydroxy,tetramethoxy(4:0/9:1)] 5,3'-Dihydroxy-6,7,4',5'-tetramethoxyflavanone | S2,S6,S5,S1,S9,S8,S7,S10 |
| 6.93 | 8.32 | 235.1693 | -0.81 | C15H22O2 | [PR] 12-Hydroxy-chiloscyphone | S2,S6,S5,S1,S9,S8,S7,S10 |
| 6.93 | 9.15 | 397.2578 | -2.33 | C22H36O6 | [FA methyl(5:1/5:2/8:0)] methyl 8-[3,5-epidioxy-2-(3-hydroperoxy-1-pentenyl)-cyclopentyl]-octanoate | S2,S6,S5,S1,S9,S8,S7,S10 |
| 6.93 | 8.86 | 426.2853 | 0.08 | C23H39NO6 | [FA hydroxy,oxo,hydroxy(3:0/2:0)] N-(1,3-dihydroxypropan-2-yl)-9-oxo-11R,15S-dihydroxy-5Z,13E-prostadienoyl amine | S2,S6,S5,S1,S9,S8,S7,S10 |
| 6.93 | 7.31 | 393.2270 | -1.10 | C22H32O6 | Isodomedin | S2,S6,S5,S1,S9,S8,S7,S10 |
| 6.94 | 8.37 | 531.2595 | 0.79 | C29H38O9 | Uscharidin | S2,S6,S5,S1,S9,S8,S7,S10 |
| 6.94 | 8.34 | 289.1437 | 0.18 | C17H20O4 | Karwinaphthol B | S2,S6,S5,S1,S9,S8,S7,S10 |
| 6.94 | 8.88 | 519.3320 | 0.33 | C30H46O7 | Cucurbitacin F | S2,S6,S5,S1,S9,S8,S7,S10 |
| 6.94 | 6.71 | 363.1440 | -0.25 | C19H22O7 | Gibberellin A8-catabolite | S2,S6,S5,S1,S9,S8,S7,S10 |
| 6.94 | 8.07 | 239.0913 | -1.33 | C12H14O5 | 3-4-5-Trimethoxycinnamicacid | S2,S6,S5,S1,S9,S8,S7,S10 |
| 6.95 | 8.41 | 277.1436 | -0.37 | C16H20O4 | Scorpioidin | S2,S6,S5,S1,S9,S8,S7,S10 |
| 6.95 | 8.89 | 543.4753 | -3.96 | C36H62O3 | [PR] 2-methyl-32,35-anhydrobacteriohopanetetrol | S2,S6,S5,S1,S9,S7,S10 |
| 6.95 | 7.06 | 387.1072 | -1.14 | C20H18O8 | Glucosyloxyanthraquinone | S2,S6,S5,S1,S9,S8,S7,S10 |
| 6.95 | 8.96 | 237.1487 | -0.37 | C14H20O3 | Heptyl p-hydroxybenzoate | S2,S6,S5,S1,S9,S8,S7,S10 |
| 6.95 | 8.39 | 181.1225 | -0.39 | C11H16O2 | 3-tert-Butyl-5-methylcatechol | S2,S6,S5,S1,S9,S8,S7,S10 |
| 6.95 | 7.80 | 321.0971 | -0.17 | C16H16O7 | 4-Coumaroylshikimate | S2,S6,S5,S1,S9,S8,S7,S10 |
| 6.95 | 7.36 | 183.0653 | -0.71 | C9H10O4 | 3-(4-Hydroxyphenyl)lactate | S2,S6,S5,S1,S9,S8,S7,S10 |
| 6.95 | 8.41 | 439.2475 | -1.54 | C27H34O5 | 7-O-Methyllicoricidin | S2,S6,S5,S1,S9,S8,S7,S10 |
| 6.96 | 7.93 | 526.3374 | -0.54 | C28H47NO8 | Pikromycin | S2,S5,S1,S9,S8,S7,S10 |
| 6.97 | 7.74 | 237.0758 | -0.86 | C12H12O5 | 5,6,7-Trimethoxycoumarin | S2,S6,S5,S1,S9,S8,S7,S10 |
| 6.97 | 8.93 | 172.1697 | -0.46 | C10H21NO | decanamide | S2,S6,S5,S1,S9,S8,S7 |
| 6.97 | 3.93 | 560.3082 | 0.14 | C28H41N5O7 | Glu-Leu-Leu-Trp | S2,S6,S1,S9,S8,S7,S10 |
| 6.97 | 8.97 | 357.2629 | -2.50 | C20H36O5 | Prostaglandin F1alpha | S2,S6,S5,S1,S9,S8,S7,S10 |
| 6.97 | 8.78 | 239.1641 | -1.05 | C14H22O3 | [FA oxo(5:1/5:0/4:0)] (1R,2R)-3-oxo-2-(2'Z-pentenyl)-cyclopentanebutanoic acid | S2,S6,S5,S1,S9,S8,S7,S10 |
| 6.97 | 8.92 | 369.2635 | -0.76 | C21H36O5 | [FA trihydroxy,methyl(2:0)] 9S,11R,15S-trihydroxy-15-methyl-5Z,13E-prostadienoic acid | S2,S6,S5,S1,S9,S8,S7,S10 |
| 6.97 | 8.90 | 251.2001 | -2.89 | C16H26O2 | Triton X-100 | S2,S6,S5,S9,S8,S7,S10 |
| 6.97 | 8.76 | 185.1536 | -1.11 | C11H20O2 | [PR] Citronellyl formate | S2,S6,S5,S1,S9,S8,S7,S10 |
| 6.98 | 8.08 | 113.0964 | 0.96 | C7H12O | [FA (7:1)] 3-heptenal | S2,S6,S5,S1,S9,S8,S7,S10 |
| 6.98 | 8.97 | 197.1323 | -2.17 | C15H16 | 1,3-Diphenylpropane | S2,S6,S5,S1,S9,S8,S7,S10 |
| 6.98 | 9.25 | 325.2374 | -0.61 | C19H32O4 | Decylubiquinol | S2,S6,S5,S1,S9,S8,S7,S10 |
| 6.98 | 8.46 | 459.3852 | 3.81 | C30H50O3 | Soyasapogenol B | S2,S6,S5,S1,S9,S8,S7,S10 |
| 6.98 | 8.46 | 731.5214 | -1.28 | C40H75O9P | PG(O-16:0/18:3(9Z,12Z,15Z)) | S2,S6,S5,S1,S9,S8,S7,S10 |
| 6.98 | 8.46 | 701.5116 | -0.36 | C39H73O8P | PA(36:2) | S2,S6,S5,S1,S9,S8,S7,S10 |
| 6.98 | 8.46 | 349.0923 | 0.69 | C17H16O8 | Dryopteric acid | S2,S6,S5,S1,S9,S8,S7 |
| 6.98 | 7.96 | 419.2452 | 1.85 | C25H30N4O2 | Naphthyl dipeptide | S2,S6,S5,S1,S9,S8,S7,S10 |
| 6.98 | 8.84 | 285.1123 | -0.06 | C17H16O4 | [Fv] Isoliquiritigenin 4,4'-dimethyl ether | S2,S6,S5,S1,S9,S8,S7,S10 |
| 6.98 | 8.97 | 689.5148 | 0.91 | C45H68O5 | DG(42:10) | S2,S6,S5,S1,S9,S8,S7,S10 |
| 6.99 | 8.93 | 241.1796 | -1.93 | C14H24O3 | [FA oxo(5:2/5:0/4:0)] (1S,2S)-3-oxo-2-pentyl-cyclopentanebutanoic acid | S2,S6,S5,S9,S8,S7,S10 |
| 6.99 | 7.86 | 309.1697 | -0.68 | C17H24O5 | Cinnamodial | S2,S6,S5,S1,S9,S8,S7,S10 |
| 6.99 | 6.79 | 168.0656 | -1.05 | C8H9NO3 | 3-Methoxyanthranilate | S2,S6,S5,S1,S9,S8,S7,S10 |
| 6.99 | 7.77 | 305.0658 | 0.03 | C15H12O7 | Taxifolin | S2,S6,S5,S1,S9,S8,S7 |
| 6.99 | 7.44 | 263.1278 | -0.79 | C15H18O4 | [PR] (+)-Marmasmic acid | S2,S6,S5,S1,S9,S8,S7,S10 |
| 6.99 | 8.49 | 773.6400 | -2.77 | C45H89O7P | PA(O-20:0/22:1(11Z)) | S2,S6,S5,S9,S8,S7,S10 |
| 7.00 | 8.88 | 355.1174 | -1.15 | C20H18O6 | Licoisoflavone A | S2,S6,S5,S1,S9,S8,S7,S10 |
| 7.00 | 8.52 | 257.0652 | 2.06 | C10H7F3N4O | carbonyl cyanide p-trifluoromethoxyphenylhydrazone | S2,S6,S5,S1,S9,S8,S7,S10 |
| 7.00 | 8.50 | 407.2060 | -1.72 | C22H30O7 | Isomontanolide | S2,S6,S5,S1,S9,S8,S7,S10 |
| 7.00 | 6.97 | 241.0708 | -0.37 | C11H12O6 | (1R,6R)-6-Hydroxy-2-succinylcyclohexa-2,4-diene-1-carboxylate | S2,S6,S5,S1,S9,S8,S7,S10 |
| 7.00 | 8.66 | 253.1436 | -0.38 | C14H20O4 | ubiquinol-1 | S2,S6,S5,S1,S9,S8,S7,S10 |
| 7.00 | 8.30 | 249.1486 | -0.64 | C15H20O3 | [PR] Parthenolide | S2,S6,S5,S1,S9,S8,S7,S10 |
| 7.00 | 4.01 | 481.1944 | 2.72 | C21H28N4O9 | Asp-Pro-Ser-Tyr | S2,S5,S1,S9,S7,S10 |
| 7.00 | 7.86 | 247.0597 | -2.60 | C13H10O5 | Isopimpinellin | S2,S6,S5,S1,S9,S8,S7 |
| 7.00 | 9.01 | 469.4026 | -3.45 | C32H52O2 | Lupeol acetate | S2,S5,S1,S9,S8,S7,S10 |
| 7.01 | 7.58 | 255.1956 | -0.28 | C15H26O3 | 3-hydroxy-15-dihydrolubimin | S2,S6,S5,S1,S9,S8,S7 |
| 7.01 | 9.02 | 535.3267 | -0.11 | C30H46O8 | Cucurbitacin H | S2,S6,S5,S1,S9,S8,S7,S10 |
| 7.01 | 8.41 | 223.0964 | -1.59 | C12H14O4 | Apiole | S2,S6,S5,S1,S9,S8,S7,S10 |
| 7.01 | 8.53 | 289.1070 | -0.84 | C16H16O5 | Alkannin | S2,S6,S5,S1,S9,S8,S7,S10 |
| 7.01 | 9.02 | 405.2640 | 0.55 | C24H36O5 | [ST hydroxy,ox] 7alpha,12alpha-Dihydroxy-3-oxochol-4-en-24-oic Acid | S6,S5,S1,S9,S8,S7,S10 |
| 7.01 | 8.96 | 342.0779 | 1.95 | C15H17N3O2Cl2 | Propiconazole | S2,S6,S5,S1,S9,S8,S7 |
| 7.02 | 8.98 | 219.1382 | 0.00 | C14H18O2 | [FA (14:5)] 5,7,9,11,13-tetradecapentaenoic acid | S2,S6,S5,S1,S9,S8,S7,S10 |
| 7.02 | 8.53 | 461.2173 | 0.27 | C25H32O8 | Aspidin | S2,S6,S5,S1,S9,S8,S7,S10 |
| 7.02 | 6.54 | 265.1435 | -0.58 | C15H20O4 | [PR trihydroxy(2:0)] (-)-1alpha,7beta,12-trihydroxy-2,9-illudadien-8-one | S2,S6,S5,S1,S9,S8,S7,S10 |
| 7.02 | 8.54 | 415.1753 | -0.13 | C23H26O7 | [Fv Trihydroxy,methoxy,hydroxy,ethyl(9:1)] 5,7,4'-Trihydroxy-3'-methoxy-6-(beta-hydroxyethyl)-8-prenylflavanone | S2,S6,S5,S1,S9,S8,S7,S10 |
| 7.02 | 8.55 | 409.2219 | -0.92 | C22H32O7 | Cascarillin | S2,S6,S5,S1,S9,S8,S7,S10 |
| 7.03 | 8.55 | 447.3473 | 0.53 | C28H46O4 | 3-Dehydroteasterone | S2,S6,S5,S1,S9,S8,S7,S10 |
| 7.03 | 8.06 | 541.1713 | 1.14 | C28H28O11 | Cleistanthin A | S2,S6,S5,S1,S9,S7 |
| 7.03 | 6.55 | 227.0912 | -2.04 | C11H14O5 | Genipin | S2,S6,S5,S1,S9,S8,S7,S10 |
| 7.03 | 6.92 | 229.1067 | -2.74 | C11H16O5 | [FA hydroxy(11:2/11:2)] 2R,9R-dihydroxy-3S,4S,7S,8S-diepoxy-5E,10-undecadien-1-ol | S2,S6,S5,S1,S9,S8,S7,S10 |
| 7.03 | 9.06 | 519.2010 | -1.03 | C30H30O8 | Gossypol | S2,S6,S5,S1,S9,S8,S7,S10 |
| 7.03 | 4.07 | 659.2837 | 1.71 | C34H38N6O8 | Gln-Trp-Tyr-Tyr | S2,S6,S8,S7,S10 |
| 7.04 | 8.95 | 195.1380 | -0.95 | C12H18O2 | 4-Hexyloxyphenol | S2,S6,S5,S1,S9,S8,S7,S10 |
| 7.04 | 8.69 | 349.1644 | -1.08 | C19H24O6 | 3-Hydroxy-5,9,17-trioxo-4,5:9,10-disecoandrosta-1(10),2-dien-4-oate | S2,S6,S5,S1,S9,S8,S7,S10 |
| 7.04 | 8.57 | 503.4268 | 3.69 | C36H54O | 2-Hexaprenylphenol | S2,S6,S5,S1,S9,S8,S7,S10 |
| 7.04 | 9.08 | 241.1070 | -1.14 | C12H16O5 | 3-carboxy-4-methyl-5-propyl-2-furanpropanoic acid | S2,S6,S5,S1,S9,S8,S7,S10 |
| 7.04 | 8.58 | 565.4621 | 0.61 | C38H60O3 | 2-Octaprenyl-3-methyl-6-methoxy-1-4-benzoquinol | S2,S6,S5,S1,S9,S8,S7,S10 |
| 7.04 | 8.36 | 221.1174 | -0.35 | C13H16O3 | Precocene 2 | S2,S6,S5,S1,S9,S8,S7,S10 |
| 7.04 | 8.13 | 379.1753 | -0.19 | C20H26O7 | Cnicin | S2,S6,S5,S1,S9,S8,S7,S10 |
| 7.04 | 8.58 | 419.0983 | 1.96 | C20H18O10 | 6-C-beta-D-Xylopyranosylluteolin | S2,S6,S5,S1,S9,S8,S7 |
| 7.05 | 8.60 | 645.2334 | 0.13 | C36H36O11 | 4'-O-Methylneobavaisoflavone 7-O-(2''-p-coumaroylglucoside) | S2,S6,S5,S9,S8,S7,S10 |
| 7.05 | 7.84 | 116.1072 | 0.27 | C6H13NO | hexanamide | S2,S6,S5,S1,S9,S8,S10 |
| 7.05 | 8.43 | 237.1122 | -0.88 | C13H16O4 | 3-Dimethylallyl-4-hydroxymandelic acid | S2,S6,S5,S1,S9,S8,S7,S10 |
| 7.05 | 4.10 | 509.1816 | 0.23 | C21H28N6O7S | Asn-Trp-Cys-Ser | S2,S6,S5,S1,S9,S7,S10 |
| 7.05 | 8.98 | 259.0967 | 0.13 | C15H14O4 | [Fv Trihydrox] 2',4',6'-Trihydroxydihydrochalcone | S2,S6,S5,S1,S9,S8,S7,S10 |
| 7.05 | 6.26 | 395.1338 | -0.33 | C19H22O9 | Aloesin | S2,S6,S5,S1,S9,S8,S7,S10 |
| 7.05 | 8.47 | 131.1070 | 1.00 | C7H14O2 | [FA methyl(6:0)] 2-methyl-hexanoic acid | S2,S6,S5,S1,S9,S8,S7,S10 |
| 7.06 | 9.11 | 617.5527 | 3.46 | C40H72O4 | [GL methyl(16:0/8:0)] 1-(10-methyl-hexadecanoyl-2-(8-[3]-ladderane-octanyl)-sn-glycerol | S6,S5,S1,S9,S8,S7,S10 |
| 7.06 | 8.15 | 191.0704 | -0.42 | C11H10O3 | 7-Ethoxycoumarin | S2,S6,S5,S1,S9,S8,S7,S10 |
| 7.06 | 6.67 | 149.0813 | 1.68 | C6H12O4 | (R)-2,3-Dihydroxy-3-methylpentanoate | S6,S5,S1,S9,S8,S7 |
| 7.06 | 6.99 | 286.2015 | 0.10 | C15H27NO4 | 2-Octenoylcarnitine | S2,S6,S5,S1,S9,S8,S7 |
| 7.06 | 6.62 | 772.6209 | -1.01 | C44H86NO7P | PC(18:0/P-18:1(11Z)) | S2,S6,S5,S1,S9,S8,S7,S10 |
| 7.06 | 8.62 | 491.2267 | -2.17 | C26H34O9 | deoxylimononic acid D-ring-lactone | S2,S6,S5,S1,S9,S8,S10 |
| 7.07 | 8.64 | 403.1030 | 0.99 | C20H18O9 | 1-Hydroxy-2-(beta-D-glucosyloxy)-9,10-anthraquinone | S2,S6,S5,S1,S9,S8,S7 |
| 7.07 | 5.42 | 395.1704 | 0.45 | C20H26O8 | Glaucarubolone | S2,S6,S5,S1,S9,S8,S7,S10 |
| 7.07 | 9.08 | 355.2476 | -1.42 | C20H34O5 | [FA trihydroxy(2:0)] 9S,11R,15S-trihydroxy-5Z,13E-prostadienoic acid | S2,S6,S5,S1,S9,S8,S7,S10 |
| 7.08 | 5.67 | 381.1911 | 0.15 | C20H28O7 | Chaparrin | S2,S6,S5,S1,S9,S8,S7,S10 |
| 7.08 | 8.66 | 433.2225 | 0.50 | C24H32O7 | Grandisin | S2,S6,S5,S1,S9,S8,S7,S10 |
| 7.08 | 8.02 | 205.0496 | -0.56 | C11H8O4 | 1,4-Dihydroxy-2-naphthoate | S2,S6,S5,S1,S9,S8,S7,S10 |
| 7.08 | 7.90 | 169.0860 | -0.85 | C9H12O3 | (2E)-2-butylidene-4-hydroxy-5-methyl-3(2H)-furanone | S2,S6,S5,S1,S9,S8,S7,S10 |
| 7.08 | 6.00 | 295.0814 | -0.23 | C14H14O7 | cyclic dehypoxanthinylfutalosine | S2,S6,S5,S1,S9,S8,S7 |
| 7.08 | 8.67 | 447.1650 | -0.51 | C23H26O9 | [Fv Hydroxy,dimethoxy(9:1)] 5-Hydroxy-7,8-dimethoxyflavanone 5-rhamnoside | S2,S6,S5,S1,S9,S8,S7,S10 |
| 7.09 | 7.56 | 309.0967 | -1.28 | C15H16O7 | Allamandin | S2,S6,S5,S1,S9,S8,S7,S10 |
| 7.09 | 8.67 | 593.4567 | 0.03 | C39H60O4 | Ubiquinol-6 | S2,S6,S5,S1,S9,S8,S7,S10 |
| 7.09 | 8.06 | 219.0654 | -0.12 | C12H10O4 | 4-Methylumbelliferyl acetate | S2,S6,S5,S1,S9,S8,S7,S10 |
| 7.09 | 4.18 | 425.1822 | 0.14 | C22H24N4O5 | Trp-Gly-Tyr | S2,S6,S5,S1,S9,S8,S7 |
| 7.09 | 7.39 | 169.0496 | -0.82 | C8H8O4 | 3,4-Dihydroxyphenylacetate | S2,S6,S5,S1,S9,S8,S7,S10 |
| 7.09 | 8.69 | 375.0722 | 2.42 | C18H14O9 | Gossypetin 7-methyl ether 8-acetate | S2,S6,S5,S1,S9,S8,S7 |
| 7.09 | 4.19 | 523.1973 | 0.27 | C22H30N6O7S | Asn-Thr-Trp-Cys | S2,S6,S5,S1,S9,S8,S7,S10 |
| 7.10 | 8.00 | 181.0858 | -1.74 | C10H12O3 | Coniferyl alcohol | S2,S6,S5,S1,S9,S8,S7,S10 |
| 7.10 | 9.20 | 693.5434 | -2.95 | C45H72O5 | DG(42:8) | S2,S6,S5,S1,S9,S8,S7,S10 |
| 7.10 | 9.20 | 275.2731 | -1.71 | C20H34 | 3,7,11,15-Tetramethylhexadeca-1,3E,6E,10E,14-pentaene | S6,S5,S1,S9,S8,S7,S10 |
| 7.10 | 9.54 | 331.2849 | 1.27 | C19H38O4 | [GL (16:0)] 1-hexadecanoyl-sn-glycerol | S2,S6,S5,S1,S9,S8 |
| 7.10 | 7.21 | 722.3977 | 2.42 | C34H59NO15 | [SP] Fumonisin B1 | S7,S10 |
| 7.11 | 9.01 | 672.5401 | -1.49 | C38H73NO8 | Galactosylceramide (d18:1/14:0) | S2,S6,S5,S1,S9,S8,S7,S10 |
| 7.11 | 8.72 | 401.1233 | -0.07 | C21H20O8 | Flavonol 3-O-D-galactoside | S2,S6,S5,S1,S9,S8,S7,S10 |
| 7.11 | 8.72 | 513.1757 | -0.03 | C27H28O10 | 6'',6''-Dimethylpyraono[2'',3'':7,8]kaempferol 4'-methyl ether 3-rhamnoside | S2,S6,S5,S1,S9,S8,S7,S10 |
| 7.11 | 7.19 | 160.0968 | -1.23 | C7H13NO3 | 5-Acetamidopentanoate | S2,S6,S5,S1,S9 |
| 7.12 | 8.20 | 384.2745 | -0.49 | C21H37NO5 | 3-Hydroxy-5, 8-tetradecadiencarnitine | S2,S6,S5,S1,S9,S8,S7,S10 |
| 7.12 | 8.74 | 583.1447 | -0.19 | C29H26O13 | 2''-O-Vanilloylvitexin | S2,S6,S9,S8,S7 |
| 7.12 | 8.80 | 357.1333 | -0.43 | C20H20O6 | [Fv] Brosimacutin G | S2,S6,S5,S1,S9,S8,S7,S10 |
| 7.12 | 8.74 | 459.1647 | -0.96 | C24H26O9 | 7-Hydroxy-5,4'-dimethoxy-8-methylisoflavone 7-O-rhamnoside | S2,S6,S5,S1,S9,S8,S7,S10 |
| 7.12 | 8.22 | 365.1590 | -1.86 | C19H24O7 | Vernomygdin | S2,S6,S5,S1,S9,S8,S7,S10 |
| 7.12 | 8.19 | 195.0650 | -1.98 | C10H10O4 | Ferulate | S6,S5,S1,S9,S8,S7,S10 |
| 7.12 | 4.24 | 569.2382 | -1.52 | C24H36N6O8S | Gln-Met-Gln-Tyr | S2,S6,S5,S1,S9,S8,S7 |
| 7.12 | 8.63 | 202.1439 | -0.68 | C10H19NO3 | Valproylglycine | S2,S5,S1,S9 |
| 7.12 | 8.12 | 339.1437 | -1.20 | C17H22O7 | 1-Peroxyferolide | S2,S6,S5,S1,S9,S8,S7,S10 |
| 7.12 | 4.25 | 512.2864 | -1.10 | C27H37N5O5 | Ile-Trp-Pro-Pro | S2,S6,S5,S1,S9,S8,S7,S10 |
| 7.13 | 8.04 | 219.1018 | -0.18 | C13H14O3 | Toxol | S2,S6,S5,S1,S9,S8,S7,S10 |
| 7.13 | 5.35 | 137.0712 | 0.14 | C7H8N2O | N-Methylnicotinamide | S2,S6,S5,S1,S9,S8,S7,S10 |
| 7.13 | 8.26 | 545.1665 | 1.78 | C27H28O12 | Premithramycin A1 | S2,S6,S5,S1,S9,S8,S7 |
| 7.13 | 7.74 | 181.0497 | -0.50 | C9H8O4 | 2-Hydroxy-3-(4-hydroxyphenyl)propenoate | S2,S6,S5,S1,S9,S8,S7,S10 |
| 7.13 | 8.51 | 221.0812 | 0.41 | C12H12O4 | Polygonolide | S2,S6,S5,S1,S9,S8,S7,S10 |
| 7.13 | 8.14 | 145.0864 | 1.53 | C7H12O3 | 7-oxoheptanoate | S2,S6,S1,S9,S7,S10 |
| 7.13 | 8.77 | 411.2377 | -0.70 | C22H34O7 | Nigakihemiacetal A | S2,S6,S5,S1,S9,S8,S7,S10 |
| 7.13 | 9.21 | 280.1914 | 1.80 | C16H25NO3 | N-vanillyl octanamide | S2,S6,S5,S1,S9,S8,S7,S10 |
| 7.14 | 8.78 | 345.1333 | -0.47 | C19H20O6 | [Fv Tetramethoxy(4:0/9:1)] 5,6,7,4'-Tetramethoxyflavanone | S2,S6,S5,S1,S9,S8,S7,S10 |
| 7.14 | 8.78 | 543.1874 | 1.97 | C28H30O11 | Ikarisoside D | S2,S6,S5,S1,S9,S8,S7 |
| 7.14 | 8.76 | 319.1177 | -0.45 | C17H18O6 | (+)-(&beta;S)-MPHPV | S2,S6,S5,S1,S9,S8,S7,S10 |
| 7.14 | 8.64 | 225.0399 | 3.74 | C8H16O2Se | 6-seleno-octanoate | S2,S6,S5,S1,S9,S8,S7 |
| 7.14 | 8.11 | 363.1804 | 0.00 | C20H26O6 | Gibberellin A36 | S2,S6,S5,S1,S9,S8,S7,S10 |
| 7.15 | 9.02 | 343.1175 | -1.11 | C19H18O6 | [Fv Methyl,trimethox] 3,4-Methylenedioxy-2',4',6'-trimethoxychalcone | S2,S6,S5,S1,S9,S8,S7,S10 |
| 7.15 | 8.79 | 437.1446 | 0.30 | C21H24O10 | Phlorizin | S2,S6,S5,S1,S9,S8,S7 |
| 7.15 | 8.79 | 467.1706 | 0.64 | C26H26O8 | Polystachin (flavone) | S2,S6,S5,S1,S9,S8,S7,S10 |
| 7.15 | 9.45 | 271.2427 | 1.79 | C20H30 | [PR] Axerophthene | S6,S5,S1,S9,S7,S10 |
| 7.15 | 8.30 | 478.2912 | -3.85 | C23H44NO7P | LysoPE(0:0/18:2(9Z,12Z)) | S2,S6,S5,S1,S9,S8,S7 |
| 7.15 | 5.64 | 297.1332 | -0.97 | C15H20O6 | [PR] Vomitoxin | S2,S6,S5,S1,S9,S8,S7,S10 |
| 7.16 | 4.32 | 517.2432 | -3.00 | C29H32N4O5 | Phe-Phe-Phe-Gly | S2,S6,S5,S1,S9,S8,S7,S10 |
| 7.16 | 8.30 | 217.0704 | -2.44 | C9H12O6 | cis-(homo)3aconitate | S2,S6,S5,S1,S9,S8,S7 |
| 7.16 | 8.58 | 371.1124 | -0.94 | C20H18O7 | Styraxin | S8,S7 |
| 7.16 | 8.82 | 471.1284 | -0.83 | C24H22O10 | Pongamoside B | S2,S6,S5,S1,S9,S8,S7 |
| 7.16 | 8.33 | 483.1654 | -3.29 | C27H28FN2OClS | SSR 125543 | S2,S6,S5,S1,S9,S8,S7,S10 |
| 7.16 | 8.33 | 818.5927 | 2.38 | C44H84NO10P | PS(38:1) | S6,S5,S1,S8,S7,S10 |
| 7.17 | 9.33 | 883.6791 | -2.44 | C58H90O6 | TG(15:1(9Z)/20:5(5Z,8Z,11Z,14Z,17Z)/20:5(5Z,8Z,11Z,14Z,17Z))[iso3] | S6,S5,S8,S7,S10 |
| 7.17 | 8.85 | 493.2437 | 0.57 | C26H36O9 | Caryoptin | S2,S6,S5,S1,S9,S8 |
| 7.18 | 9.27 | 375.1442 | 0.36 | C20H22O7 | [Fv] Rubone | S2,S6,S5,S1,S9,S8,S7,S10 |
| 7.18 | 6.12 | 295.1173 | -1.87 | C15H18O6 | Tutin | S2,S6,S5,S1,S9,S8,S7,S10 |
| 7.18 | 6.53 | 117.0185 | 0.01 | C4H4O4 | Formylpyruvate | S2,S8,S7 |
| 7.19 | 5.04 | 358.1493 | -1.54 | C16H23NO8 | Bakankoside | S2,S6,S5,S1,S9 |
| 7.19 | 8.87 | 545.2381 | -0.49 | C29H36O10 | 10-Deacetylbaccatin III | S2,S6,S5,S1,S9,S8,S7,S10 |
| 7.19 | 8.88 | 783.5565 | 3.60 | C44H79O9P | PG(O-18:0/20:5(5Z,8Z,11Z,14Z,17Z)) | S6,S5,S1,S9,S8,S7,S10 |
| 7.19 | 8.23 | 211.0964 | -1.48 | C11H14O4 | Sinapyl alcohol | S2,S6,S5,S1,S9,S8,S7,S10 |
| 7.19 | 8.47 | 287.0550 | -0.78 | C15H10O6 | [Fv] Luteolin | S2,S6,S5,S1,S9,S8,S7 |
| 7.19 | 8.36 | 261.0401 | 1.77 | C13H8O6 | Norathyriol | S2,S6,S5,S1,S9,S8,S7 |
| 7.20 | 4.27 | 313.1276 | -2.52 | C15H20O7 | Nivalenol | S2,S6,S5,S1,S9,S8,S7,S10 |
| 7.20 | 9.39 | 409.1497 | 0.42 | C20H24O9 | Ginkgolide A | S2,S6,S5,S1,S9,S8,S7,S10 |
| 7.20 | 4.98 | 130.0500 | -0.43 | C5H7NO3 | L-1-Pyrroline-3-hydroxy-5-carboxylate | S2 |
| 7.20 | 8.37 | 200.0704 | -2.10 | C12H9NO2 | Dictamnine | S2,S6,S5,S1,S9,S8,S7 |
| 7.20 | 9.12 | 400.2329 | -0.65 | C20H33NO7 | Candoxatrilat | S2,S6,S5,S1,S9,S8,S7,S10 |
| 7.20 | 9.21 | 862.6263 | 1.27 | C46H87NO13 | Lactosylceramide(d18 | S6,S5,S1,S9,S8,S7,S10 |
| 7.21 | 8.63 | 277.0704 | -1.74 | C14H12O6 | heptaketide pyrone | S2,S6,S5,S1,S9,S8,S7 |
| 7.21 | 7.91 | 265.1184 | -0.38 | C13H16N2O4 | alpha-N-Phenylacetyl-L-glutamine | S2 |
| 7.21 | 8.72 | 361.1284 | 0.06 | C19H20O7 | Elephantopin | S2,S6,S5,S1,S9,S8,S7,S10 |
| 7.22 | 8.93 | 405.1189 | 1.57 | C20H20O9 | [Fv] Chalconaringenin 2'-xyloside | S2,S6,S5,S1,S9,S8,S7 |
| 7.22 | 9.43 | 723.5916 | -1.17 | C47H78O5 | [GL (22:1/22:6)] 1-(13Z-docosenoyl)-2-(4Z,7Z,10Z,13Z,16Z,19Z-docosahexaenoyl)-sn-glycerol | S2,S6,S5,S1,S9,S8,S7,S10 |
| 7.22 | 9.02 | 323.1279 | -0.43 | C20H18O4 | Abyssinone I | S2,S6,S5,S1,S9,S8,S7,S10 |
| 7.22 | 8.94 | 501.1395 | 0.32 | C25H24O11 | Epigallocatechin 5,3',5'-trimethyl ether 3-O-gallate | S2,S6,S5,S1,S9,S8,S7 |
| 7.22 | 8.26 | 233.0446 | -0.54 | C12H8O5 | 5-Hydroxyxanthotoxin | S2,S6,S5,S1,S9,S8,S7 |
| 7.23 | 9.22 | 293.0302 | -0.27 | C14H12O3S2 | 5-(3-Hydroxy-4-acetoxybut-1-ynyl)-2,2'-bithiophene | S2,S6,S5,S1,S8,S7 |
| 7.23 | 7.30 | 227.0553 | 0.11 | C10H10O6 | Chorismate | S2,S6,S5,S1,S9,S8,S7 |
| 7.23 | 7.23 | 294.2069 | 1.17 | C17H27NO3 | Pramoxine | S2,S6,S5,S1,S9 |
| 7.24 | 8.70 | 363.1078 | 0.36 | C18H18O8 | hydratedusnate | S2,S6,S5,S1,S9,S8,S7,S10 |
| 7.24 | 8.98 | 283.0811 | -1.31 | C26H28O14 | [Fv] Leptosidin 6-xylosyl-(1->4)-arabinoside | S2,S6,S5,S1,S9,S8,S7,S10 |
| 7.24 | 8.98 | 377.0872 | 0.59 | C18H16O9 | 5,7,3',4'-Tetrahydroxy-3,6,5'-trimethoxyflavone | S2,S6,S5,S1,S9,S8,S7 |
| 7.24 | 8.48 | 221.0447 | 0.18 | C11H8O5 | (3E)-4-(2-Carboxyphenyl)-2-oxobut-3-enoate | S2,S6,S5,S1,S9,S8,S7 |
| 7.24 | 9.49 | 457.4060 | 3.78 | C31H52O2 | [PR (2:0)] 8(26),14(27)-onoceradiene-3beta,21alpha-diol | S2,S5,S1,S8,S7,S10 |
| 7.25 | 9.46 | 277.0350 | -1.16 | C14H12O2S2 | 5-(4-Acetoxybut-1-ynyl)-2,2'-bithiophene | S2,S6,S5,S1,S9,S8,S7 |
| 7.25 | 9.50 | 665.5156 | 2.14 | C43H68O5 | DG(40:8) | S2,S6,S5,S1,S9,S8,S7,S10 |
| 7.25 | 8.07 | 117.0552 | 2.92 | C5H8O3 | 3-Methyl-2-oxobutanoic acid | S6,S5,S1,S9,S8,S7,S10 |
| 7.25 | 9.51 | 505.3903 | 2.55 | C31H52O5 | [ST hydroxy(3:0)] (5Z,7E)-(1R,2R,3R)-2-(4-hydroxybutoxy)-9,10-seco-5,7,10(19)-cholestatriene-1,3,25-triol | S2,S5,S1,S9,S8,S7,S10 |
| 7.26 | 8.98 | 359.1127 | -0.28 | C19H18O7 | [Fv] Scaberin | S2,S6,S5,S1,S9,S8,S7,S10 |
| 7.26 | 8.75 | 307.0453 | 0.78 | C14H10O8 | 2-Protocatechoylphloroglucinolcarboxylate | S2,S6,S5,S1,S9,S8,S7 |
| 7.27 | 7.55 | 199.0600 | -1.65 | C9H10O5 | 3-Methoxy-4-hydroxymandelate | S2,S6,S5,S1,S9,S8,S7 |
| 7.27 | 7.49 | 172.0971 | 0.41 | C8H13NO3 | N-Butyryl-L-homoserine lactone | S2,S6,S5,S1,S9,S8,S7 |
| 7.28 | 9.51 | 298.2014 | -0.29 | C16H27NO4 | N-(3-Oxododecanoyl)homoserine lactone | S2,S6,S5,S1,S9 |
| 7.28 | 9.05 | 571.2166 | 1.36 | C27H34N6O4S2 | bistratamide A | S2,S6,S5,S1,S9,S8,S7,S10 |
| 7.28 | 9.06 | 664.6243 | 0.36 | C42H81NO4 | N-(2-hydroxytetracosanoyl)-4,8-sphingadienine | S2,S6,S5,S1,S9,S8,S7,S10 |
| 7.28 | 9.35 | 333.0613 | -1.41 | C15H22OClBr | [PR] 3beta-bromo-2alpha-chloro-7-chamigren-9-one | S2,S6,S5,S1,S9,S8,S7 |
| 7.28 | 7.22 | 174.0550 | -1.14 | C10H7NO2 | 2-Quinolinecarboxylic acid | S6,S5,S1,S9,S8,S7 |
| 7.28 | 7.47 | 371.1345 | 1.58 | C17H22O9 | sinapaldehyde glucoside | S2,S6,S5,S1,S9,S8 |
| 7.28 | 7.64 | 297.0966 | -1.85 | C14H16O7 | dehypoxanthine futalosine | S2,S6,S5,S1,S9,S8,S7,S10 |
| 7.28 | 8.64 | 345.0612 | 1.33 | C17H12O8 | 3,4,3'-Tri-O-methylellagic acid | S2,S6,S5,S9,S8,S7 |
| 7.29 | 9.08 | 389.0871 | 0.49 | C19H16O9 | 5,7,2',5'-Tetrahydroxy-3,4'-dimethoxyflavone 5'-acetate | S2,S6,S5,S1,S9,S8,S7 |
| 7.29 | 7.87 | 155.0340 | -0.51 | C7H6O4 | 2,5-Dihydroxybenzoate | S2,S6,S5,S1,S9,S8,S7,S10 |
| 7.30 | 6.19 | 116.0708 | 0.17 | C5H9NO2 | 3-acetamidopropanal | S2,S6,S5,S1,S9,S8,S7,S10 |
| 7.30 | 4.60 | 610.2885 | 1.81 | C31H39N5O8 | Glu-Leu-Trp-Tyr | S2,S6,S1,S9,S10 |
| 7.30 | 8.60 | 406.2953 | -0.37 | C24H39NO4 | Cassaine | S2,S1,S9,S8,S7,S10 |
| 7.30 | 6.82 | 130.0863 | -1.74 | C6H11NO2 | N4-Acetylaminobutanal | S2,S6,S5,S1,S9,S8,S7 |
| 7.31 | 7.31 | 464.2404 | -3.44 | C26H31N4O4 | ketoconazol | S6,S5,S1,S9,S7 |
| 7.31 | 8.35 | 337.0921 | 0.36 | C16H16O8 | 5-O-Caffeoylshikimic acid | S6,S8,S7 |
| 7.31 | 7.50 | 327.1073 | -1.22 | C15H18O8 | 4-O-beta-D-Glucosyl-4-hydroxycinnamate | S2,S6,S5,S1,S9,S8,S7 |
| 7.31 | 4.63 | 488.2518 | 2.48 | C24H33N5O6 | Gln-Phe-Pro-Pro | S2,S6,S1 |
| 7.32 | 9.63 | 455.3880 | -1.36 | C31H50O2 | [ST (4:0/4:0/4:0)] (5Z,7E)-(3S)-9,10-seco-5,7,10(19)-cholestatrien-3-butanoate | S2,S6,S5,S8,S7,S10 |
| 7.32 | 8.79 | 317.0662 | 1.34 | C16H12O7 | Isorhamnetin | S2,S6,S5,S1,S9,S8,S7,S10 |
| 7.32 | 9.15 | 487.1248 | 2.24 | C24H22O11 | Flavonol 3-O-(6-O-malonyl-beta-D-glucoside) | S2,S6,S5,S1,S9,S8,S7 |
| 7.33 | 8.65 | 806.5917 | 1.12 | C43H84NO10P | [PS (18:0/19:0)] 1-octadecanoyl-2-nonadecanoyl-sn-glycero-3-phosphoserine | S2,S6,S5,S1,S9,S8,S7,S10 |
| 7.33 | 7.00 | 147.0922 | 2.09 | C9H10N2 | Dimethylbenzimidazole | S6,S1,S9,S10 |
| 7.33 | 9.15 | 391.1035 | 2.35 | C19H18O9 | Clitoriacetal | S2,S6,S5,S1,S9,S8,S7 |
| 7.33 | 7.78 | 198.1129 | 1.01 | C10H15NO3 | Tenuazonic acid | S2,S6,S5,S1,S9,S8,S7,S10 |
| 7.33 | 7.52 | 87.0446 | 3.72 | C4H6O2 | Diacetyl | S6,S5,S1,S9,S8,S7,S10 |
| 7.33 | 9.66 | 441.1401 | 1.72 | C20H24O11 | Ginkgolide C | S2,S6,S5,S1,S9,S8,S7 |
| 7.34 | 4.67 | 534.2344 | 0.30 | C20H35N7O8S | Gln-Met-Gln-Gln | S2,S6,S5,S8,S7,S10 |
| 7.34 | 8.68 | 719.3795 | -1.48 | C43H50N4O6 | Vobtusine | S2,S7,S10 |
| 7.34 | 7.60 | 323.0763 | -0.26 | C15H14O8 | Leucodelphinidin | S2,S6,S5,S1,S9,S8,S7 |
| 7.35 | 9.19 | 457.3663 | -3.40 | C30H48O3 | Ursolic acid | S2,S6,S5,S1,S9,S8,S7,S10 |
| 7.35 | 8.69 | 856.6089 | 2.88 | C47H86NO10P | PS(19:1(9Z)/22:2(13Z,16Z)) | S5,S8,S7,S10 |
| 7.35 | 6.15 | 260.1129 | -0.68 | C11H17NO6 | Proacacipetalin | S6,S5,S1,S9,S8 |
| 7.36 | 4.92 | 175.0598 | -2.97 | C7H10O5 | Shikimate | S2,S6,S5,S1,S9,S8,S7,S10 |
| 7.36 | 9.72 | 547.4016 | 3.86 | C33H54O6 | cholest-5,24-dien-3beta-ol 3-O-beta-D-glucopyranoside | S2,S6,S5,S1,S9,S8,S7,S10 |
| 7.36 | 6.92 | 386.3049 | -1.89 | C25H39NO2 | 3beta-(1-Pyrrolidinyl)-5alpha-pregnane-11,20-dione | S2,S5,S9,S8,S7 |
| 7.37 | 9.23 | 873.6614 | 3.68 | C49H93O10P | PG(21:0/22:2(13Z,16Z)) | S6,S5,S1,S9,S8,S7,S10 |
| 7.37 | 8.75 | 533.1307 | 2.85 | C25H24O13 | Biochanin A 7-O-(6-O-malonyl-beta-D-glucoside) | S2,S6,S5,S1,S9,S8,S7 |
| 7.38 | 8.00 | 171.0289 | -0.84 | C7H6O5 | 2,3,4-trihydroxybenzoate | S6,S5,S1,S9,S8,S7,S10 |
| 7.39 | 7.68 | 355.1040 | 4.07 | C16H18O9 | Chlorogenate | S2,S6,S5,S1,S9,S8,S10 |
| 7.39 | 9.79 | 603.5708 | -0.81 | C40H74O3 | [GL methyl(16:0/8:0)] 1-(10-methyl-hexadecanyl-2-(8-[3]-ladderane-octanyl)-sn-glycerol | S6,S1,S9,S8,S7,S10 |
| 7.39 | 8.79 | 690.5096 | 3.72 | C37H72NO8P | PE(32:1) | S2,S6,S5,S1,S9,S8,S7,S10 |
| 7.40 | 8.29 | 476.2784 | 2.21 | C23H42NO7P | 1-18:3-lysophosphatidylethanolamine | S2,S6,S5,S1,S9,S8,S7 |
| 7.40 | 10.12 | 359.2933 | -3.94 | C24H38O2 | [FA (12:0)] 12-[5]-ladderane-dodecanoic acid | S2,S1,S9,S10 |
| 7.40 | 9.30 | 807.5529 | -0.90 | C46H79O9P | PG(P-18:0/22:6(4Z,7Z,10Z,13Z,16Z,19Z)) | S2,S6,S5,S1,S9,S8,S7,S10 |
| 7.40 | 9.31 | 475.1252 | 3.11 | C23H22O11 | Fujikinetin 7-O-glucoside | S2,S6,S5,S1,S9,S8,S7 |
| 7.41 | 9.31 | 423.0933 | 2.16 | C19H18O11 | Norswertianolin | S2,S6,S5,S1,S9,S8,S7 |
| 7.41 | 9.32 | 549.4146 | -1.05 | C33H56O6 | Cholesteryl-beta-D-glucoside | S2,S5,S1,S9,S7 |
| 7.42 | 8.26 | 174.1126 | -0.24 | C8H15NO3 | N-Acetyl-L-leucine | S2,S6,S5,S1,S9,S8,S7 |
| 7.42 | 8.84 | 830.5931 | 2.80 | C45H84NO10P | PS(17:0/22:2(13Z,16Z)) | S5,S7,S10 |
| 7.42 | 4.84 | 614.2474 | 1.02 | C30H39N5O5S2 | Met-Met-Phe-Trp | S2,S6,S1 |
| 7.42 | 9.34 | 641.1498 | -0.88 | C31H28O15 | Quercetin 3-(6''-ferulylglucoside) | S6,S8 |
| 7.42 | 8.76 | 233.0806 | -2.04 | C13H12O4 | aloesone | S2,S6,S5,S1,S9,S8,S7,S10 |
| 7.43 | 7.27 | 216.0869 | 0.25 | C9H13NO5 | Succinyl proline | S2,S6,S5,S1,S9,S8 |
| 7.43 | 7.04 | 301.0917 | -1.13 | C13H16O8 | 4-(beta-D-Glucosyloxy)benzoate | S2,S6,S5,S1,S9,S7 |
| 7.43 | 9.36 | 363.0716 | 0.84 | C17H14O9 | 5,7,3',4',5'-Pentahydroxy-3,6-dimethoxyflavone | S2,S6,S5,S1,S9,S8,S7 |
| 7.44 | 6.49 | 288.1954 | -2.34 | C18H25NO2 | Isolobinine | S2,S6,S5,S1,S9,S8 |
| 7.44 | 9.38 | 587.1420 | 3.83 | C28H26O14 | [Fv] Prunin 6''-O-gallate | S2,S6,S8 |
| 7.44 | 9.88 | 561.4886 | 1.12 | C36H64O4 | [PR] 2-methylbacteriohopane-32,33,34,35-tetrol | S6,S5,S1,S9,S8,S7,S10 |
| 7.44 | 7.57 | 187.0597 | -3.37 | C8H10O5 | 3-hydroxy-3-carboxy-4,5-cyclopropylhex-5-enoate | S2,S6,S5,S1,S9,S8,S7,S10 |
| 7.44 | 8.22 | 213.0394 | -0.85 | C9H8O6 | 2-Hydroxy-6-ketononatrienedioate | S2,S6,S5,S8,S7 |
| 7.44 | 6.00 | 374.1069 | -4.05 | C15H19NO10 | DIMBOA-glucoside | S2,S6,S5,S1,S9,S8,S7 |
| 7.44 | 8.27 | 196.0607 | -0.04 | C9H9NO4 | Salicyluric acid | S2,S6,S5,S1,S9,S8,S7 |
| 7.45 | 8.90 | 243.0498 | -2.57 | C6H13NO7P | [PE] 1-acyl-sn-glycero-3-phosphoethanolamine | S2,S6,S5,S1,S9,S8,S7 |
| 7.45 | 7.45 | 447.0952 | -1.34 | C18H22O11S | Paederoside | S2,S6,S5,S1,S9,S8,S7 |
| 7.45 | 6.82 | 388.1248 | 1.87 | C16H21NO10 | HMDBOA-Glc | S2,S6,S5,S1,S9,S8 |
| 7.46 | 8.12 | 329.0870 | 0.23 | C14H16O9 | 2-Succinyl-5-enolpyruvyl-6-hydroxy-3-cyclohexene-1-carboxylate | S2,S6,S5,S1,S9,S8,S7 |
| 7.46 | 7.08 | 183.0287 | -1.58 | C8H6O5 | Stipitatate | S2,S6,S5,S1,S8,S7 |
| 7.46 | 7.55 | 144.0656 | -1.04 | C6H9NO3 | Vinylacetylglycine | S2,S6,S5,S1,S9 |
| 7.47 | 4.29 | 248.2017 | 2.33 | C16H25NO | Lycopodine | S2,S6,S5,S9,S7 |
| 7.48 | 6.93 | 147.0287 | -2.01 | C5H6O5 | 2-Oxoglutarate | S2,S6,S9,S8,S7 |
| 7.48 | 9.47 | 407.0985 | 2.41 | C19H18O10 | 5,7,3',5'-Tetrahydroxy-3,6,8,4'-tetramethoxyflavone | S2,S6,S5,S1,S9,S8,S7 |
| 7.49 | 4.35 | 155.0109 | 2.07 | C3H7O5P | Propanoyl phosphate | S2,S9 |
| 7.49 | 9.49 | 335.0402 | 0.58 | C15H10O9 | Hibiscetin | S2,S6,S5,S8,S7 |
| 7.49 | 9.87 | 315.1229 | -0.11 | C18H18O5 | [Fv] Flavokawin A | S2,S6,S5,S1,S9,S8,S7,S10 |
| 7.49 | 9.51 | 127.1119 | -0.53 | C8H14O | [FA (8:1)] 2-octenal | S2,S6,S5,S1,S9,S8,S7,S10 |
| 7.50 | 7.47 | 341.0871 | 0.32 | C15H16O9 | Daphnin | S2,S6,S5,S1,S9,S8,S7 |
| 7.51 | 9.51 | 656.6181 | -1.34 | C40H81NO5 | N-(2-hydroxydocosanoyl)-phytosphingosine | S2,S6,S5,S1,S9,S8,S7,S10 |
| 7.52 | 5.48 | 144.9821 | 2.35 | C2H6ClO3P | 2-chloroethylphosphonate | S6,S5,S1,S9,S8,S7,S10 |
| 7.52 | 10.04 | 551.5040 | 0.68 | C35H66O4 | 1-O-(1Z-Tetradecenyl)-2-(9Z-octadecenoyl)-sn-glycerol | S2,S6,S5,S1,S9,S8,S7 |
| 7.53 | 9.57 | 507.1502 | 0.50 | C24H26O12 | [Fv] Okanin 4-methyl ether 3'-(6''-acetylglucoside) | S6,S5,S1,S9 |
| 7.53 | 8.17 | 124.0759 | -0.33 | C7H9NO | 4-hydroxylaminotoluene | S2,S6,S5,S1,S9,S7,S10 |
| 7.53 | 10.07 | 418.3329 | 2.61 | C26H43NO3 | [ST (3:0)] (5Z,7E)-(1S,3R)-23-aza-9,10-seco-5,7,10(19)-cholestatriene-1,3,25-triol | S5 |
| 7.54 | 5.08 | 582.2568 | 1.18 | C29H35N5O8 | Asp-Trp-Val-Tyr | S2,S6,S5,S1,S9,S8,S7,S10 |
| 7.55 | 10.09 | 407.3671 | -0.91 | C30H46 | [PR] 4,4'-Diapophytofluene | S6,S5,S1,S9,S8,S7,S10 |
| 7.55 | 9.89 | 564.5368 | 2.73 | C36H69NO3 | [SP (18:0)] N-(9Z-octadecenoyl)-sphing-4-enine | S2,S5,S1,S9,S10 |
| 7.55 | 7.55 | 517.0972 | -2.59 | C16H26N2O13P2 | dTDP-beta-L-rhodinose | S2,S6,S1,S9,S8,S7 |
| 7.55 | 6.68 | 142.0503 | 1.23 | C6H7NO3 | 6-oxo-1,4,5,6-tetrahydronicotinate | S2,S6,S5,S1,S9,S8,S7 |
| 7.55 | 9.61 | 678.6384 | -1.87 | C43H83NO4 | N-(2-hydroxypentacosanoyl)-4,8-sphingadienine | S2,S6,S5,S1,S9,S8,S7,S10 |
| 7.56 | 9.11 | 463.0877 | 0.82 | C21H18O12 | Luteolin 7-O-glucuronide | S2,S6,S5,S1,S9,S8,S7 |
| 7.56 | 9.29 | 186.0914 | -0.88 | C12H11NO | N-Hydroxy-4-aminobiphenyl | S2,S6,S5,S1,S9,S8,S7,S10 |
| 7.57 | 9.63 | 451.0879 | 1.37 | C20H18O12 | Quercetin 4'-galactoside | S2,S6,S5,S1,S9,S8,S7 |
| 7.57 | 8.02 | 114.0552 | 0.40 | C5H7NO2 | 1-Pyrroline-2-carboxylate | S2,S6,S5,S1,S9,S8,S7 |
| 7.57 | 8.66 | 190.0502 | 0.47 | C10H7NO3 | Kynurenate | S2,S6,S5,S1,S9,S8,S7 |
| 7.58 | 9.65 | 572.5248 | -0.45 | C34H69NO5 | N-(2-hydroxyhexadecanoyl)-phytosphingosine | S6,S5,S1,S9,S10 |
| 7.58 | 6.53 | 188.0556 | -0.09 | C7H9NO5 | 2-(Acetamidomethylene)succinate | S2,S6,S9,S8,S7 |
| 7.58 | 7.56 | 177.1124 | 0.02 | C8H16O4 | L-Cladinose | S2,S6,S5,S1,S9,S8,S7,S10 |
| 7.58 | 7.56 | 154.0502 | 0.47 | C7H7NO3 | 3-Hydroxyanthranilate | S2,S6,S5,S9,S8,S7,S10 |
| 7.59 | 9.29 | 297.0603 | -1.34 | C13H12O8 | Phaseolic acid | S2,S6,S1,S9,S8,S7 |
| 7.59 | 8.77 | 303.0504 | 0.77 | C15H10O7 | Delphinidin | S2,S6,S5,S1,S9,S8,S7 |
| 7.60 | 7.94 | 338.0883 | 3.09 | C15H15NO8 | 2-8-Dihydroxyquinoline-beta-D-glucuronide | S2,S6,S5,S1,S9,S8,S7 |
| 7.61 | 8.46 | 128.0708 | -0.03 | C6H9NO2 | delta1-Piperideine-2-carboxylate | S2,S6,S5,S1,S9,S8,S7,S10 |
| 7.63 | 7.82 | 214.1115 | -3.72 | C8H15N5S | Simetryn | S2,S6,S5,S1,S9,S8,S10 |
| 7.64 | 7.70 | 353.0864 | -1.47 | C16H16O9 | 4-Methylumbelliferone glucuronide | S2,S6,S5,S1,S8,S7,S10 |
| 7.64 | 7.79 | 440.2786 | 2.73 | C20H42NO7P | [PC (6:2/6:0)] 1-hexyl-2-hexanoyl-sn-glycero-3-phosphocholine | S2,S6,S5,S1,S9,S8 |
| 7.65 | 9.81 | 483.1151 | 3.27 | C21H22O13 | [Fv hydroxy(7:0)] 3,4,2',3',4',6',alpha-Heptahydroxychalcone 2'-glucoside | S2,S6,S5,S1,S9,S8,S7,S10 |
| 7.66 | 7.66 | 547.1102 | 2.10 | C17H28N2O14P2 | dTDP-beta-L-mycarose | S2,S6,S5,S1,S9,S8,S7 |
| 7.67 | 8.69 | 194.0960 | -3.32 | C14H11N | 2-Anthramine | S2,S6,S5,S1,S9,S8 |
| 7.67 | 9.33 | 832.6071 | 0.83 | C45H86NO10P | PS(17:0/22:1(11Z)) | S2,S6,S5,S8,S7,S10 |
| 7.67 | 10.35 | 662.4992 | -2.14 | C40H63N5O3 | [PR] adenosylhopane | S2,S6,S5,S1,S9,S8,S7,S10 |
| 7.70 | 9.40 | 493.0981 | 0.42 | C22H20O13 | Carmine | S2,S6,S5,S1,S9,S8,S7 |
| 7.71 | 7.92 | 468.2721 | -0.41 | C21H42NO8P | [PC (6:0/7:0)] 1-hexanoyl-2-heptanoyl-sn-glycero-3-phosphocholine | S2,S6,S5,S1,S9,S8 |
| 7.71 | 10.43 | 603.5348 | -0.11 | C39H70O4 | [GL methyl(15:0/8:0)] 1-(14-methyl-pentadecanoyl)-2-(8-[3]-ladderane-octanyl)-sn-glycerol | S2,S6,S5,S1,S9,S8,S7,S10 |
| 7.72 | 9.94 | 670.6343 | -0.55 | C41H83NO5 | N-(2-hydroxytricosanoyl)-phytosphingosine | S6,S5,S1,S9,S8,S7 |
| 7.72 | 9.94 | 569.3821 | 1.06 | C28H57O9P | PG(22:0/0:0) | S2,S1,S9,S7 |
| 7.73 | 8.96 | 452.2769 | -1.01 | C21H42NO7P | 1-16:1-lysophosphatidylethanolamine | S2,S6,S5,S1,S9,S8,S7 |
| 7.73 | 5.70 | 294.1176 | -3.34 | C11H19NO8 | N-Acetylmuramate | S6,S5,S1,S9,S8,S7 |
| 7.77 | 9.53 | 480.3076 | -2.26 | C23H46NO7P | [PE (18:1)] 1-(9Z-octadecenoyl)-sn-glycero-3-phosphoethanolamine | S2,S6,S5,S1,S9,S8,S7,S10 |
| 7.77 | 8.28 | 209.0088 | 2.56 | C9H4O6 | Stipitatonate | S2,S6,S5,S1,S9,S8,S7 |
| 7.79 | 8.99 | 89.0603 | 3.84 | C4H8O2 | Butanoic acid | S2,S6,S5,S1,S9,S8,S7,S10 |
| 7.82 | 10.15 | 217.1049 | 2.51 | C27H28O5 | Aspulvinone H | S2,S6,S5,S9,S8,S7,S10 |
| 7.84 | 10.18 | 479.0828 | 1.19 | C21H18O13 | Isoetin 4'-glucuronide | S2,S6,S5,S1,S9,S8,S7 |
| 7.88 | 7.59 | 122.0967 | 0.41 | C8H11N | N,N-Dimethylaniline | S2,S6,S5,S1,S9,S8,S7,S10 |
| 7.91 | 10.81 | 774.5892 | 1.50 | C46H79NO8 | DGCC(16:0/20:5) | S2,S6,S5,S1,S9,S8,S7,S10 |
| 7.96 | 8.73 | 227.1760 | 1.79 | C12H22N2O2 | 1,8-Diazacyclotetradecane-2,9-dione | S2,S6,S5,S1,S9,S8,S7,S10 |
| 7.96 | 9.52 | 321.0610 | 0.84 | C15H12O8 | Dihydromyricetin | S2,S6,S5,S8,S7 |
| 7.97 | 8.45 | 494.3240 | -0.70 | C24H48NO7P | [PC (16:1)] 1-(9Z-hexadecenoyl)-sn-glycero-3-phosphocholine | S2,S6,S5,S1,S9,S10 |
| 7.98 | 7.77 | 107.0706 | 1.16 | C4H10O3 | Diethylene glycol | S2,S6,S5,S1,S9,S8,S7,S10 |
| 8.00 | 9.59 | 146.0601 | -1.20 | C9H7NO | 3-Methyleneoxindole | S2,S6,S5,S1,S9,S8,S7,S10 |
| 8.04 | 6.75 | 302.1392 | 0.88 | C17H19NO4 | 6-O-Methylnorlaudanosoline | S2,S6,S5,S1,S9,S8,S10 |
| 8.05 | 10.04 | 265.0974 | -0.08 | C16H12N2O2 | Perlolyrine | S2,S6,S1,S9 |
| 8.05 | 6.09 | 433.2042 | -0.43 | C16H28N6O8 | Asn-Val-Asn-Ser | S2,S6,S1,S9,S7,S10 |
| 8.05 | 10.61 | 684.6500 | -0.37 | C42H85NO5 | N-(2-hydroxytetracosanoyl)-phytosphingosine | S2,S6,S5,S1,S9,S8,S7 |
| 8.06 | 10.63 | 832.2409 | -1.68 | C39H43O20 | Alatanin 2 | S2,S6,S5,S1,S9,S8,S7,S10 |
| 8.07 | 10.63 | 359.0409 | 2.51 | C17H10O9 | Distemonanthin | S2,S6,S5,S1,S9,S8,S7 |
| 8.07 | 11.15 | 705.5829 | 1.53 | C47H76O4 | [ST (20:4)] cholest-5-en-3beta-yl (15S-hydroperoxy-5Z,8Z,12E,14Z-eicosatetraenoate) | S2,S6,S5,S1,S9,S8,S7,S10 |
| 8.08 | 9.62 | 295.0447 | -1.18 | C13H10O8 | Tricrozarin A | S2,S6,S5,S1,S9,S8,S7 |
| 8.08 | 10.66 | 395.0610 | -0.29 | C34H28O22 | 1,2,3,6-Tetrakis-O-galloyl-beta-D-glucose | S2,S6,S5,S1,S9,S8,S7 |
| 8.10 | 11.01 | 220.1121 | -1.00 | C16H13N | N-Phenyl-1-naphthylamine | S2,S6,S5,S1,S9,S8,S7,S10 |
| 8.11 | 10.73 | 980.2784 | -1.06 | C44H51O25 | Cyanidin 3-(6''-sinapylsophoroside)-5-glucoside | S2,S6 |
| 8.16 | 10.82 | 809.5172 | -0.65 | C41H77O13P | PI(32:1) | S2,S5,S7,S10 |
| 8.18 | 9.87 | 565.2824 | 2.16 | C34H36N4O4 | protoporphyrinogen | S2,S6,S1,S9,S8,S7,S10 |
| 8.21 | 10.53 | 263.0812 | -2.10 | C16H10N2O2 | indigo | S6,S1,S9 |
| 8.24 | 8.97 | 782.5717 | 2.55 | C44H80NO8P | PC(36:4) | S2,S6,S5,S8,S7,S10 |
| 8.24 | 9.60 | 353.0503 | -0.74 | C15H12O10 | OH-DDVA meta-cleavage product | S2,S6,S5,S1,S9,S8,S7 |
| 8.27 | 7.66 | 153.07 | -0.47 | C7H8N2O2 | N1-Methyl-2-pyridone-5-carboxamide | S2,S6,S5,S1,S9,S8,S7,S10 |
| 8.32 | 9.79 | 199.06 | -2.14 | C9H10O5 | 3-(3,4-Dihydroxyphenyl)lactate | S2,S6,S5,S1,S9,S8,S7,S10 |
| 8.34 | 7.93 | 250.18 | -1.10 | C15H23NO2 | Lophocerine | S6,S5,S1,S9,S8,S7,S10 |
| 8.35 | 9.30 | 248.08 | 0.62 | C9H13NO7 | N-Succinyl-L-glutamate | S2,S6,S5,S1,S9,S8,S7 |
| 8.36 | 11.71 | 736.61 | 1.40 | C44H81NO7 | [GL trimethyl(16:0/18:2)] 1-hexadecanoyl-2-(9Z,12Z-octadecadienoyl)-sn-glycero-3-O-(N,N,N-trimethyl)-homoserine | S6,S5,S8,S7 |
| 8.36 | 10.44 | 135.04 | -0.46 | C8H6O2 | [FA (8:1/2:0)] 6Z-Octene-2,4-diynoic acid | S2,S6,S5,S1,S9,S8,S7,S10 |
| 8.37 | 10.49 | 139.04 | -0.92 | C7H6O3 | Gentisate aldehyde | S2,S6,S5,S1,S9,S8,S7,S10 |
| 8.38 | 11.27 | 450.32 | 0.06 | C26H43NO5 | Glycodeoxycholate | S8,S7 |
| 8.39 | 10.57 | 254.07 | 1.04 | C11H11NO6 | N-Pyruvoyl-5-methoxy-3-hydroxyanthranilate | S2,S6,S5,S1,S9,S8,S7 |
| 8.40 | 8.80 | 113.03 | 0.42 | C4H4N2O2 | Uracil | S2,S6,S5,S1,S9,S8,S7 |
| 8.41 | 11.81 | 446.33 | 1.83 | C27H43NO4 | [ST oxo(3:0)] (5Z,7E)-(1S,3R,24R)-23-aza-22-oxo-9,10-seco-5,7,10(19)-ergostatriene-1,3,25-triol | S8,S7,S10 |
| 8.42 | 10.84 | 680.45 | -0.22 | C34H66NO10P | PS(28:0) | S2,S5,S1,S9,S10 |
| 8.42 | 10.53 | 163.04 | -0.83 | C9H6O3 | 4-hydroxycoumarin | S2,S6,S5,S1,S9,S8,S7,S10 |
| 8.44 | 10.66 | 291.09 | 0.69 | C15H14O6 | (+)-Catechin | S2,S6,S5,S1,S9,S8,S7 |
| 8.45 | 10.89 | 666.43 | -0.57 | C33H64NO10P | PS(12:0/15:0) | S2,S5,S1,S9,S8,S7,S10 |
| 8.47 | 10.80 | 149.06 | -0.89 | C9H8O2 | 4-Hydroxycinnamyl aldehyde | S2,S6,S5,S1,S9,S8,S7,S10 |
| 8.47 | 8.43 | 124.04 | 0.03 | C6H5NO2 | Nicotinate | S2,S6,S5,S1,S9,S8,S7 |
| 8.48 | 12.04 | 363.23 | -0.18 | C20H30N2O4 | Calpeptin | S2,S6,S1,S9,S10 |
| 8.52 | 12.03 | 737.66 | -3.57 | C46H88O6 | TG(12:0/12:0/19:0)[iso3] | S6,S7,S10 |
| 8.53 | 11.55 | 594.37 | -3.45 | C29H56NO9P | PC(16:0/5:0(CHO)) | S2,S6,S5,S9,S8,S7,S10 |
| 8.53 | 11.34 | 259.06 | -0.50 | C14H10O5 | Gentisin | S2,S6,S5,S1,S9,S8,S7,S10 |
| 8.57 | 6.81 | 354.17 | 0.32 | C21H23NO4 | (S)-N-Methylcanadine | S2,S6,S5,S1,S9,S8,S7,S10 |
| 8.58 | 9.61 | 206.04 | -1.13 | C10H7NO4 | Xanthurenic acid | S2,S6,S5,S1,S9,S8,S7 |
| 8.58 | 12.17 | 419.32 | 0.69 | C26H42O4 | [ST (3:0)] (5Z,7E)-(1S,3R,20R,24R)-22-oxa-9,10-seco-5,7,10(19)-cholestatriene-1,3,24-triol | S2,S6,S5,S1,S9,S8,S7,S10 |
| 8.60 | 12.20 | 643.53 | -3.74 | C41H70O5 | DG(38:5) | S2,S6,S5,S1,S9,S8,S7 |
| 8.61 | 7.22 | 518.30 | -2.81 | C26H39N5O6 | Ile-Leu-Trp-Ser | S2,S10 |
| 8.62 | 11.70 | 388.31 | -0.11 | C21H41NO5 | 2-Hydroxymyristoylcarnitine | S2,S6,S5,S1,S9,S8,S7,S10 |
| 8.68 | 9.99 | 340.16 | 2.98 | C20H21NO4 | (S)-Canadine | S2,S6,S5,S1,S9,S8,S7,S10 |
| 8.68 | 11.35 | 638.40 | -2.11 | C31H60NO10P | PS(13:0/12:0) | S2,S5,S8,S7,S10 |
| 8.68 | 9.20 | 274.20 | 0.18 | C14H27NO4 | Heptanoylcarnitine | S2,S6,S1,S9,S8,S7 |
| 8.68 | 8.68 | 422.29 | 0.38 | C24H39NO5 | Talatizamine | S2,S6,S5,S1,S9,S8,S7,S10 |
| 8.69 | 12.42 | 200.20 | 0.04 | C12H25NO | Dodecanamide | S2,S6,S5,S1,S9,S8,S7,S10 |
| 8.70 | 10.08 | 370.17 | 0.22 | C21H23NO5 | Allocryptopine | S2,S6,S5,S1,S9,S8,S7,S10 |
| 8.70 | 11.32 | 301.07 | 0.28 | C16H12O6 | Peonidin | S2,S6,S5,S1,S9,S8,S7 |
| 8.73 | 9.84 | 157.01 | -2.09 | C6H4O5 | 2-5-Furandicarboxylicacid | S2,S6,S8,S7,S10 |
| 8.77 | 11.96 | 344.28 | -0.07 | C19H37NO4 | Dodecanoylcarnitine | S2,S5,S1,S9,S8 |
| 8.79 | 11.29 | 174.05 | -1.79 | C10H7NO2 | 4-Quinolinecarboxylic acid | S2,S6,S5,S1,S9,S8,S7,S10 |
| 8.79 | 12.00 | 342.26 | 1.08 | C19H35NO4 | trans-2-Dodecenoylcarnitine | S2,S6,S5,S1,S9,S8,S10 |
| 8.80 | 10.11 | 844.72 | 2.93 | C49H98NO7P | PC(P-20:0/21:0) | S5,S1,S9,S8,S7 |
| 8.81 | 10.97 | 400.14 | 1.86 | C19H21N5O3S | Oxmetidine | S2,S6,S5,S1,S9,S8,S7,S10 |
| 8.81 | 12.63 | 551.48 | 0.67 | C38H62O2 | [PR] all-trans-retinyl oleate | S6,S5,S8,S7,S10 |
| 8.83 | 12.65 | 293.21 | 0.20 | C18H28O3 | [FA (18:3)] 13-keto-9Z,11E,15Z-octadecatrienoic acid | S2,S6,S1,S9,S8,S7,S10 |
| 8.85 | 12.70 | 186.11 | 2.44 | C10H17OS | 2E-decenoyl-[acp] | S6,S1,S9,S8 |
| 8.85 | 12.50 | 728.60 | 0.63 | C42H81NO8 | Glucosylceramide (d18:1/18:0) | S2,S6,S5,S1,S9,S8,S7,S10 |
| 8.86 | 12.52 | 462.34 | -1.29 | C24H47NO7 | Psychosine | S9,S8,S7 |
| 8.86 | 7.74 | 288.12 | -3.10 | C16H17NO4 | (S)-Norlaudanosoline | S2,S6,S5,S1,S9,S8,S7,S10 |
| 8.88 | 12.88 | 269.23 | 0.31 | C20H28 | anhydroretinol | S6,S5,S8,S7,S10 |
| 8.89 | 10.27 | 538.35 | -0.84 | C26H52NO8P | [PC acetyl(16:0)] 2-acetyl-3-hexadecanoyl-sn-glycero-1-phosphocholine | S2,S5 |
| 8.89 | 12.78 | 384.31 | -0.06 | C22H41NO4 | N-oleoyl threonine | S2,S1,S9,S8,S7 |
| 8.91 | 12.81 | 547.45 | -0.03 | C38H58O2 | [PR] all-trans-retinyl linolate | S6,S8,S7,S10 |
| 8.92 | 10.90 | 157.05 | 0.47 | C7H8O4 | 2-Hydroxy-6-keto-2,4-heptadienoate | S2,S6,S5,S1,S9,S8,S7,S10 |
| 8.92 | 10.81 | 246.08 | -1.91 | C13H11NO4 | Haplopine | S2,S6,S5,S1,S9,S8,S7,S10 |
| 8.93 | 12.86 | 342.30 | 3.66 | C20H39NO3 | N-palmitoyl GABA | S2,S6,S5,S1,S9,S8,S7,S10 |
| 8.95 | 12.41 | 376.31 | 3.34 | C52H78O3 | Nonaprenyl-4-hydroxybenzoate | S2,S6,S5,S1,S9,S8,S7,S10 |
| 8.96 | 12.88 | 277.22 | 1.00 | C18H28O2 | [FA (6:0)] 6-[3]-ladderane-hexanoic acid | S2,S6,S1,S9,S8,S7,S10 |
| 8.96 | 9.78 | 128.03 | -0.73 | C5H5NO3 | 2,3,6-Trihydroxypyridine | S2,S6,S5,S1,S9,S8,S7 |
| 8.96 | 7.92 | 532.28 | -2.85 | C26H37N5O7 | Asp-Leu-Trp-Val | S2,S6,S5,S1,S9,S8,S7,S10 |
| 8.97 | 10.71 | 187.02 | -3.57 | C7H6O6 | Maleylpyruvate | S6,S8,S7 |
| 8.97 | 12.85 | 272.26 | 0.19 | C16H33NO2 | [FA amino(16:0)] 2R-aminohexadecanoic acid | S2,S6,S5,S1,S9,S8,S7,S10 |
| 8.99 | 7.92 | 358.17 | 1.77 | C20H23NO5 | deacetylcolchicine | S2,S6,S5,S1,S9,S8,S7,S10 |
| 9.00 | 12.50 | 465.10 | -2.96 | C21H20O12 | [Fv] Bractein | S2,S6,S5,S1,S8,S7 |
| 9.02 | 13.03 | 417.30 | -4.07 | C26H40O4 | [ST (3:0)] (5Z,7E)-(1S,3R,24R,25S)-25,26-epoxy-27-nor-9,10-seco-5,7,10(19)-cholestatriene-1,3,24-triol | S2,S1,S9,S8,S10 |
| 9.07 | 13.15 | 877.72 | -4.07 | C57H96O6 | [GL (16:1/18:2/20:4)] 1-(9Z-hexadecenoyl)-2-(9Z,12Z-octadecadienoyl)-3-(5Z,8Z,11Z,14Z-eicosatetraenoyl)-sn-glycerol | S6,S5,S1,S9,S8,S7,S10 |
| 9.08 | 9.06 | 124.08 | -0.12 | C7H9NO | 2-amino-4-methylphenol | S2,S6,S5,S1,S9,S8,S7,S10 |
| 9.13 | 11.26 | 432.30 | -0.02 | C22H41NO7 | [SP amino,trihydroxy(20:0)] 2S-amino-5S-acetoxy-3R,4R,14R-trihydroxyeicos-6E-enoic acid | S6,S5,S1,S9,S8,S7,S10 |
| 9.14 | 8.28 | 276.09 | 3.96 | C24H30N4O7S2 | Cys-Cys-Tyr-Tyr | S2,S6,S5,S1,S9,S8,S7,S10 |
| 9.14 | 11.57 | 242.14 | 0.27 | C10H19N5S | prometryn | S8 |
| 9.17 | 12.83 | 233.10 | 0.12 | C27H28O7 | Pumilaisoflavone B | S6,S5,S9,S10 |
| 9.17 | 10.24 | 322.08 | -1.03 | C11H15NO10 | beta-Citryl-L-glutamic acid | S6,S5,S1,S9,S8,S7 |
| 9.21 | 8.42 | 268.11 | 1.15 | C20H34N6O9S | Glu-Met-Gln-Gln | S2,S6,S5,S1,S9,S7 |
| 9.21 | 10.93 | 536.37 | -0.06 | C27H54NO7P | [PC (16:2/3:0)] 1-hexadecyl-2-(2E-propionyl)-sn-glycero-3-phosphocholine | S2,S6,S5,S1,S8,S7 |
| 9.22 | 11.44 | 390.29 | 0.99 | C20H39NO6 | [SP amino,hydroxy(4:0/20:0)] 2S-amino-3R,4R,5S,14R-tetrahydroxyeicos-6E-enoic acid | S2,S6,S5,S1,S9,S8,S7,S10 |
| 9.24 | 12.97 | 473.23 | 0.77 | C30H32O5 | [Fv] Euchrenone a14 | S2,S6,S5,S1,S9,S8,S7,S10 |
| 9.26 | 10.10 | 333.12 | 2.81 | C13H21N4O2ClS | Tos-Arg-CH2Cl | S2,S6,S5,S1,S9,S8,S7,S10 |
| 9.27 | 11.48 | 342.17 | 0.43 | C20H23NO4 | 5,8,13,13a-Tetrahydrocolumbamine | S2,S6,S5,S1,S9,S8,S7,S10 |
| 9.27 | 12.32 | 160.08 | -1.35 | C10H9NO | 3-Methyl-quinolin-2-ol | S2,S6,S5,S1,S9,S8,S7,S10 |
| 9.28 | 12.56 | 406.17 | 1.92 | C24H23NO5 | (+/-)-6-Acetonyldihydrochelerythrine | S2,S6,S5,S1,S9,S8,S7,S10 |
| 9.30 | 11.52 | 217.03 | -2.41 | C8H8O7 | 5-Carboxy-2-oxohept-3-enedioate | S2,S6,S1,S8 |
| 9.33 | 13.15 | 737.57 | -2.30 | C40H81O9P | PG(O-20:0/14:0) | S2,S6,S5,S1,S9,S8,S10 |
| 9.34 | 13.17 | 737.55 | 1.17 | C43H77O7P | PA(O-20:0/20:5(5Z,8Z,11Z,14Z,17Z)) | S2,S6,S5,S1,S9,S8,S7,S10 |
| 9.34 | 11.18 | 522.35 | -2.05 | C26H52NO7P | 1-Oleoylglycerophosphocholine | S2,S6,S5,S1,S9,S8,S7,S10 |
| 9.34 | 10.43 | 295.12 | -2.30 | C15H18O6 | Tutin | S6,S5,S1,S9,S8,S7,S10 |
| 9.35 | 5.43 | 128.07 | -0.86 | C6H9NO2 | alpha-(Methylenecyclopropyl)glycine | S2,S6,S5,S1,S9,S8,S7,S10 |
| 9.35 | 12.62 | 260.09 | -0.66 | C14H13NO4 | Kokusaginine | S2,S6,S5,S1,S9,S8,S7 |
| 9.41 | 13.32 | 258.08 | -0.56 | C14H11NO4 | N-Benzoyl-4-hydroxyanthranilate | S2,S6,S5,S1,S9,S8,S7 |
| 9.42 | 12.20 | 194.10 | -0.49 | C14H11N | 2-Anthramine | S2,S6,S5,S1,S9,S8,S7 |
| 9.44 | 8.87 | 265.09 | -3.61 | C22H32N4O7S2 | Glu-Met-Phe-Cys | S2,S6,S5,S1,S9,S8,S7,S10 |
| 9.45 | 6.23 | 138.09 | -0.65 | C8H11NO | 1-(p-Hydroxyphenyl)ethylamine | S2,S6,S5,S1,S9,S8,S7,S10 |
| 9.45 | 10.11 | 153.04 | -0.64 | C5H4N4O2 | Xanthine | S6,S5,S9 |
| 9.47 | 11.45 | 430.32 | 0.84 | C23H43NO6 | Hexadecanedioicacidmono-L-carnitineester | S2,S1,S9,S8,S7 |
| 9.50 | 11.50 | 482.32 | -1.47 | C23H48NO7P | [PC (15:0)] 1-pentadecanoyl-sn-glycero-3-phosphocholine | S2,S6,S5,S8,S10 |
| 9.51 | 12.52 | 342.10 | -3.34 | C12H23NO6S2 | 4-methylthiobutyldesulfoglucosinolate | S2,S6,S5,S1,S9,S8,S7 |
| 9.53 | 12.12 | 356.19 | 2.13 | C21H25NO4 | Tetrahydropalmatine | S2,S6,S5,S1,S9,S8,S7,S10 |
| 9.59 | 11.67 | 761.65 | 0.90 | C43H89N2O6P | SM(d18:0/20:0) | S2,S6,S5,S1,S9,S8,S7,S10 |
| 9.62 | 12.34 | 370.20 | 2.25 | C22H27NO4 | Corydaline | S2,S6,S5,S1,S9,S8,S7,S10 |
| 9.64 | 13.79 | 315.12 | 0.02 | C18H18O5 | [Fv] Helilandin B | S2,S6,S5,S1,S9,S8,S7,S10 |
| 9.65 | 13.30 | 736.49 | -1.10 | C41H70NO8P | PE(36:6) | S2,S6,S5,S1,S9,S8,S7,S10 |
| 9.67 | 13.44 | 399.11 | 2.15 | C21H18O8 | Auramycinone | S2,S6,S5,S1,S9,S8,S7 |
| 9.68 | 13.35 | 710.48 | -0.20 | C39H68NO8P | PE(34:5) | S2,S6,S5,S1,S8,S7,S10 |
| 9.68 | 13.86 | 624.45 | -0.53 | C35H61NO8 | Antibiotic TA | S2,S6,S5,S1,S7 |
| 9.70 | 11.90 | 733.62 | 1.75 | C41H85N2O6P | SM(d18:0/18:0) | S2,S6,S5,S1,S8,S7 |
| 9.71 | 13.92 | 587.32 | 1.13 | C33H46O9 | 5-Oxoavermectin ''2b'' aglycone | S2,S6,S5,S1,S9,S8,S7,S10 |
| 9.72 | 13.93 | 433.17 | 0.73 | C26H24O6 | Artocommunol CA | S2,S6,S5,S1,S9,S8,S7,S10 |
| 9.72 | 11.94 | 524.37 | -0.18 | C26H54NO7P | [PC (18:0)] 1-octadecanoyl-sn-glycero-3-phosphocholine | S2,S6,S5,S1,S9,S8,S7,S10 |
| 9.74 | 11.99 | 496.34 | 0.77 | C24H50NO7P | [PC (16:0)] 1-hexadecanoyl-sn-glycero-3-phosphocholine | S2,S6,S5,S1,S9,S8,S7,S10 |
| 9.75 | 12.53 | 336.20 | -0.69 | C22H25NO2 | Lobelanine | S2,S6,S5,S1,S9,S8,S7,S10 |
| 9.77 | 14.05 | 543.49 | -0.66 | C40H62 | Phytofluene | S6,S5,S1,S8,S7,S10 |
| 9.82 | 12.13 | 732.55 | -0.48 | C40H78NO8P | PC(32:1) | S2,S6,S5,S1,S8,S7,S10 |
| 9.82 | 14.15 | 435.18 | -0.21 | C26H26O6 | Mundulone | S2,S9,S8,S7,S10 |
| 9.83 | 12.16 | 508.34 | 1.98 | C25H50NO7P | [PC (17:0)] 1-(10Z-heptadecenoyl)-sn-glycero-3-phosphocholine | S2,S6,S5,S7 |
| 9.83 | 14.16 | 773.51 | -0.49 | C45H73O8P | PA(42:8) | S2,S6,S5,S1,S8,S7,S10 |
| 9.83 | 10.50 | 266.12 | -0.90 | C17H15NO2 | (-)-Annonaine | S2,S6,S5,S1,S9,S8,S7,S10 |
| 9.84 | 12.18 | 720.59 | -1.33 | C40H82NO7P | [PC (14:2/18:0)] 1-tetradecyl-2-octadecanoyl-sn-glycero-3-phosphocholine | S2,S6,S5,S1,S9,S8,S7,S10 |
| 9.85 | 12.20 | 506.36 | 1.70 | C26H52NO6P | [PC (18:2)] 1-(1Z,9Z-octadecadienyl)-sn-glycero-3-phosphocholine | S2,S6,S5,S1,S9,S8,S7,S10 |
| 9.86 | 12.22 | 706.54 | 3.55 | C38H76NO8P | PC(30:0) | S2,S6,S5,S1,S8,S7,S10 |
| 9.86 | 12.23 | 734.57 | 2.61 | C40H80NO8P | PC(32:0) | S2,S6,S5,S1,S8,S10 |
| 9.86 | 12.23 | 789.68 | -0.01 | C45H93N2O6P | SM(d18:0/22:0) | S2,S6,S5,S1,S9,S8,S7,S10 |
| 9.87 | 14.24 | 385.09 | 0.72 | C20H16O8 | hydroxyversicolorone | S2,S6,S5,S1,S9,S8,S7 |
| 9.88 | 12.27 | 816.59 | 3.27 | C48H82NO7P | PC(22:6(4Z,7Z,10Z,13Z,16Z,19Z)/P-18:1(9Z)) | S2,S6,S5,S1,S9,S8,S7,S10 |
| 9.89 | 13.72 | 394.29 | -1.17 | C25H35N3O | Undecylprodigiosin | S2,S1,S9 |
| 9.89 | 12.28 | 704.52 | -0.82 | C38H74NO8P | [PC (14:0/16:1)] 1-tetradecanoyl-2-(9Z-hexadecenoyl)-sn-glycero-3-phosphocholine | S2,S5,S1,S9,S8,S7,S10 |
| 9.89 | 14.29 | 707.50 | -0.63 | C41H71O7P | PA(O-16:0/22:6(4Z,7Z,10Z,13Z,16Z,19Z)) | S2,S6,S5,S1,S9,S8,S7,S10 |
| 9.90 | 14.31 | 769.49 | -0.88 | C38H73O13P | PI(12:0/17:0) | S2,S6,S5,S1,S9,S8,S7,S10 |
| 9.91 | 13.82 | 402.41 | 0.63 | C28H51N | 3beta,4-Dimethyl-4-aza-5alpha-cholestane | S2,S6,S5,S1,S9,S8,S7,S10 |
| 9.91 | 14.33 | 582.55 | 0.35 | C36H71NO4 | 4-hydroxysphing-8(Z)-enine-18:0, ceramide | S2,S6,S5,S1,S9,S8,S7,S10 |
| 9.92 | 14.33 | 771.49 | -2.63 | C45H71O8P | PA(42:9) | S2,S6,S5,S8,S7,S10 |
| 9.93 | 14.35 | 745.48 | -0.91 | C43H69O8P | PA(40:8) | S2,S6,S5,S1,S8,S7,S10 |
| 9.93 | 14.36 | 681.49 | 1.88 | C39H69O7P | PA(O-16:0/20:5(5Z,8Z,11Z,14Z,17Z)) | S2,S5,S1,S9,S8,S7,S10 |
| 9.93 | 14.36 | 628.59 | -2.81 | C38H77NO5 | N-(2-hydroxyicosanoyl)-phytosphingosine | S2,S6,S5,S1,S9,S8,S7,S10 |
| 9.93 | 12.36 | 746.61 | -1.07 | C42H84NO7P | PC(16:0/P-18:0) | S2,S6,S5,S1,S9,S8,S7,S10 |
| 9.94 | 8.66 | 346.18 | 2.66 | C15H27N3O4S | Met-Val-Pro | S2,S6,S5,S9,S8,S7,S10 |
| 9.95 | 14.40 | 636.59 | 0.90 | C40H77NO4 | N-(2-hydroxydocosanoyl)-4,8-sphingadienine | S2,S6,S5,S1,S9,S8,S7,S10 |
| 9.95 | 11.23 | 364.32 | -1.56 | C23H41NO2 | Terminaline | S2,S6,S5,S1,S9,S8,S7,S10 |
| 9.96 | 12.43 | 706.61 | -0.25 | C40H84NO6P | [PC (12:2/20:2)] 1-dodecyl-2-eicosyl-sn-glycero-3-phosphocholine | S2,S6,S5,S1,S9,S8,S7,S10 |
| 9.96 | 14.43 | 434.12 | -3.37 | C21H21O10 | Fragarin | S2,S6,S5,S1,S9,S8,S7 |
| 9.97 | 14.44 | 741.45 | 3.25 | C43H65O8P | PA(18:4(6Z,9Z,12Z,15Z)/22:6(4Z,7Z,10Z,13Z,16Z,19Z)) | S2,S6,S5,S1,S9,S8,S7,S10 |
| 9.97 | 12.62 | 187.06 | -2.53 | C8H10O5 | 2-hydroxy-3-carboxy-4,5-cyclopropylhex-5-enoate | S2,S6,S5,S1,S9,S8,S7,S10 |
| 9.97 | 14.44 | 411.36 | -1.87 | C29H46O | 4alpha-Methyl-5alpha-ergosta-8,14,24(28)-trien-3beta-ol | S6,S1,S9,S8,S7,S10 |
| 9.98 | 14.46 | 783.50 | -1.91 | C39H75O13P | PI(18:0/12:0) | S2,S6,S1,S9,S8,S7,S10 |
| 9.99 | 14.49 | 610.58 | 0.65 | C38H75NO4 | 4-hydroxysphing-8(Z)-enine-20:0, ceramide | S2,S6,S5,S1,S9,S8,S7,S10 |
| 10.00 | 10.00 | 326.38 | 0.71 | C22H47N | di-n-Undecylamine | S2,S6,S5,S1,S9,S8,S7,S10 |
| 10.01 | 12.16 | 288.25 | -1.38 | C16H33NO3 | [SP hydroxy,hydroxy,methyl(10:2/2:0)] 6R-(8-hydroxydecyl)-2R-(hydroxymethyl)-piperidin-3R-ol | S2,S6,S5,S1,S9,S8,S7,S10 |
| 10.02 | 14.04 | 746.53 | 1.84 | C40H76NO9P | PS(O-16:0/18:2(9Z,12Z)) | S2,S6,S5,S1,S9,S8,S7,S10 |
| 10.03 | 9.46 | 258.15 | 0.33 | C16H19NO2 | (+/-)-gamma-Lycorane | S2,S6,S5,S1,S9,S8,S10 |
| 10.04 | 14.09 | 736.51 | 1.18 | C38H74NO10P | PS(32:0) | S2,S6,S5,S1,S9,S8,S7,S10 |
| 10.05 | 14.39 | 331.23 | -0.61 | C21H30O3 | 11alpha-Hydroxyprogesterone | S2,S6,S5,S1,S9,S8,S7,S10 |
| 10.06 | 13.87 | 149.06 | -0.89 | C9H8O2 | Dihydrocoumarin | S2,S6,S5,S1,S9,S8,S7,S10 |
| 10.06 | 10.49 | 137.05 | -1.55 | C5H4N4O | Hypoxanthine | S2,S6,S5,S1,S9,S8,S7,S10 |
| 10.08 | 12.65 | 830.57 | 1.68 | C48H80NO8P | PC(40:8) | S8,S7 |
| 10.09 | 14.07 | 221.12 | -3.05 | C13H16O3 | Flossonol | S2,S6,S5,S1,S9,S8,S7,S10 |
| 10.11 | 14.22 | 706.45 | -4.10 | C39H63NO10 | &beta;-chaconine | S2,S6,S5,S1,S9,S8,S7,S10 |
| 10.12 | 14.25 | 578.41 | 0.08 | C33H55NO7 | O-glucosyl-tomatidine | S2,S6,S5,S1,S9,S8,S7,S10 |
| 10.14 | 12.78 | 564.37 | 0.78 | C28H54NO8P | [PC acetyl(18:1)] 1-(9E-octadecenoyl)-2-acetyl-sn-glycero-3-phosphocholine | S2,S6,S1,S9,S8,S7,S10 |
| 10.16 | 14.67 | 301.18 | -0.57 | C19H24O3 | 19-Oxoandrost-4-ene-3,17-dione | S2,S6,S5,S1,S9,S8,S7,S10 |
| 10.17 | 5.61 | 184.17 | 0.42 | C11H21NO | Tecostanine | S2,S6,S5,S1,S9,S8,S7,S10 |
| 10.19 | 10.39 | 445.12 | -2.05 | C17H24N4O6S2 | Cys-Cys-Gly-Tyr | S2,S6,S5,S1,S9,S8,S7,S10 |
| 10.21 | 13.42 | 370.37 | -0.08 | C23H47NO2 | [SP hydroxy,methyl,methyl(18:2/2:0)] 2S-(hydroxymethyl)-4S-(12-methyloctadecyl)azetidin-3R-ol | S2,S6,S5,S9,S8,S7,S10 |
| 10.23 | 5.78 | 332.15 | 0.16 | C18H21NO5 | Ambelline | S2,S6,S5,S1,S9,S8,S7,S10 |
| 10.24 | 14.76 | 329.21 | -0.06 | C21H28O3 | 6beta,19-Epoxypregn-4-ene-3,20-dione | S2,S6,S5,S1,S9,S8,S7,S10 |
| 10.24 | 14.04 | 162.06 | -0.82 | C9H7NO2 | 4,8-Dihydroxyquinoline | S6,S5,S1,S9,S8,S7 |
| 10.24 | 14.48 | 704.44 | -1.28 | C39H61NO10 | Spinosyn P | S2,S6,S5,S1,S9,S8,S7,S10 |
| 10.24 | 14.36 | 279.16 | -0.27 | C16H22O4 | [PR] Emmotin A | S2,S6,S5,S1,S9,S8,S7,S10 |
| 10.24 | 10.49 | 530.23 | -2.34 | C24H31N7O7 | Asn-Trp-Asn-Pro | S2,S6,S1,S8,S7,S10 |
| 10.25 | 13.50 | 444.37 | -1.35 | C25H49NO5 | 12-Hydroxy-12-octadecanoylcarnitine | S2,S6,S5,S1,S9,S8,S7,S10 |
| 10.26 | 14.51 | 610.30 | 0.05 | C35H39N5O5 | Ergocristine | S2,S6,S9,S8,S7,S10 |
| 10.26 | 10.51 | 519.14 | -2.44 | C20H30N4O6S3 | Cys-Met-Cys-Tyr | S2,S6,S5,S1,S9,S8,S7,S10 |
| 10.26 | 14.65 | 293.21 | -2.11 | C18H28O3 | 17beta-Hydroxy-2-oxa-5alpha-androstan-3-one | S2,S6,S5,S1,S9,S8,S7,S10 |
| 10.26 | 14.53 | 483.27 | 3.15 | C22H43O9P | 2-16:1-lysoPG | S2,S6,S7 |
| 10.27 | 14.65 | 315.23 | 0.84 | C21H30O2 | 3,5-Cyclo-5alpha,17alpha-pregn-20-yne-6beta,17-diol | S2,S6,S1,S9,S8,S7,S10 |
| 10.28 | 14.77 | 279.23 | 0.18 | C18H30O2 | [ST (2:0)] estrane-3alpha,17alpha-diol | S2,S6,S5,S1,S9,S8,S7,S10 |
| 10.28 | 13.56 | 737.53 | -2.10 | C41H72N2O9 | [SP] Oceanalin A | S2,S6,S1,S9,S8,S7,S10 |
| 10.28 | 13.07 | 467.36 | 1.98 | C23H51N2O5P | LysoSM(d18:0) | S2,S5,S1,S9,S8,S7 |
| 10.30 | 14.58 | 155.14 | -0.02 | C10H18O | [PR] 1,8-Cineol | S2,S6,S5,S1,S9,S7 |
| 10.32 | 14.64 | 560.39 | -1.15 | C33H53NO6 | &gamma;-solanine | S9,S8,S7 |
| 10.33 | 14.87 | 310.27 | -0.50 | C19H35NO2 | [SP methyl(3:0)] 9-methyl-sphinga-4E,8E,10E-trienine | S2,S6,S5,S1,S9,S8,S7 |
| 10.33 | 13.81 | 183.03 | 0.35 | C8H6O5 | 4-Hydroxyphthalate | S6,S1,S8,S7 |
| 10.34 | 14.67 | 510.34 | -0.73 | C28H47NO7 | Narbomycin | S2,S6,S5,S1,S9,S8,S7,S10 |
| 10.37 | 14.53 | 245.09 | 2.03 | C13H12N2O3 | Indolylacryloylglycine | S2,S6,S5,S1,S9,S10 |
| 10.38 | 13.41 | 340.15 | -0.15 | C20H21NO4 | (R)-Canadine | S2,S6,S5,S1,S9,S10 |
| 10.39 | 13.27 | 466.35 | -3.25 | C23H50N2O5P | LysoSM(d18:1) | S2,S6,S5,S1,S9,S8,S7,S10 |
| 10.39 | 12.84 | 346.31 | -0.43 | C23H39NO | Irehine | S2,S6,S5,S1,S9,S8,S7,S10 |
| 10.39 | 7.86 | 230.10 | 2.42 | C9H15N3O2S | Ergothioneine | S2,S6,S5,S1,S9,S8,S7,S10 |
| 10.40 | 13.91 | 318.28 | 0.67 | C21H35NO | Tridihexethyl | S2,S6,S5,S1,S9,S8,S7,S10 |
| 10.40 | 10.67 | 210.11 | -1.57 | C11H15NO3 | Tyr-OEt | S2,S6,S5,S1,S9,S8,S7,S10 |
| 10.43 | 14.85 | 416.35 | -0.17 | C27H45NO2 | Tomatidine | S2,S6,S5,S1,S9,S8,S7,S10 |
| 10.43 | 14.95 | 296.26 | -2.23 | C18H33NO2 | [SP (3:0)] sphinga-4E,8E,10E-trienine | S2,S6,S5,S1,S9,S8,S7,S10 |
| 10.43 | 14.85 | 506.35 | -2.11 | C29H47NO6 | Mycinamicin VIII | S2,S6,S5,S1,S9,S8,S7,S10 |
| 10.45 | 11.35 | 160.13 | -0.62 | C8H17NO2 | [FA amino(8:0)] 3-amino-octanoic acid | S6,S1,S8,S7 |
| 10.48 | 13.47 | 616.18 | 0.57 | C34H32FeN4O4 | ferriprotoporphyrin IX | S2,S8,S7 |
| 10.48 | 13.97 | 230.25 | 0.24 | C14H31NO | [SP (14:0)] 1-deoxy-tetradecasphinganine | S2,S6,S5,S1,S9,S8,S7,S10 |
| 10.51 | 14.01 | 442.35 | -1.64 | C25H47NO5 | 3-Hydroxy-11Z-octadecenoylcarnitine | S2,S5,S8,S7,S10 |
| 10.51 | 11.03 | 507.28 | 2.04 | C25H38N4O7 | Asp-Leu-Leu-Phe | S2,S6,S1,S9,S8,S7,S10 |
| 10.52 | 15.03 | 554.35 | 2.67 | C26H52NO9P | PS(20:0/0:0) | S2,S5,S1,S9,S8,S7,S10 |
| 10.52 | 14.55 | 480.27 | -0.18 | C29H37NO5 | Lythramine | S2,S6,S5,S1,S9,S8,S7 |
| 10.53 | 11.06 | 570.27 | 1.65 | C32H35N5O5 | Ala-Phe-Phe-Trp | S2,S6,S8,S7,S10 |
| 10.53 | 13.57 | 492.33 | 1.05 | C24H48N2O6P | [SP] N-(acyl)-sphing-4-enine-1-phosphocholine | S2,S6,S5,S1,S9,S8,S7,S10 |
| 10.54 | 15.20 | 278.25 | -0.06 | C18H31NO | [SP (4:0)] 1-deoxy-sphinga-6Z,9Z,12Z,15Z-tetraenine | S2,S6,S5,S1,S9,S8,S7,S10 |
| 10.55 | 15.16 | 301.22 | 0.72 | C20H28O2 | 19-Nor-17alpha-pregn-5(10)-en-20-yne-3alpha,17beta-diol | S2,S6,S1,S9,S8,S7,S10 |
| 10.56 | 15.12 | 522.34 | -1.29 | C29H47NO7 | Mycinamicin VII | S2,S6,S5,S1,S9,S8,S7,S10 |
| 10.56 | 15.12 | 568.39 | 2.14 | C31H53NO8 | 5-O-beta-D-Mycaminosyltylactone | S2,S1,S8,S7,S10 |
| 10.57 | 15.13 | 408.27 | 0.60 | C23H37NO5 | Norerythrostachaldine | S2,S6,S5,S1,S9,S8,S7,S10 |
| 10.60 | 15.20 | 528.35 | 0.64 | C28H49NO8 | Dihydropicromycin | S2,S6,S5,S1,S9,S8,S7,S10 |
| 10.62 | 14.09 | 244.11 | 1.48 | C10H17N3O2S | Biotin amide | S2,S6,S5,S1,S9,S8,S7,S10 |
| 10.62 | 15.39 | 286.28 | 2.72 | C17H35NO2 | [SP methyl(16:0)] 15-methyl-hexadecasphing-4E-enine | S2,S6,S5,S1,S9,S8,S7,S10 |
| 10.62 | 10.47 | 360.25 | 0.60 | C22H33NO3 | Ajaconine | S2,S6,S5,S1,S9,S8,S7 |
| 10.63 | 15.27 | 426.30 | -0.05 | C27H39NO3 | Jervine | S2,S6,S5,S9,S8,S7,S10 |
| 10.64 | 6.74 | 118.09 | 0.03 | C5H11NO2 | 5-Aminopentanoate | S2,S6,S1,S9,S8,S7 |
| 10.65 | 10.65 | 422.29 | -0.58 | C24H39NO5 | Talatizamine | S2,S6,S5,S1,S9,S8,S7,S10 |
| 10.65 | 14.29 | 368.22 | -2.74 | C23H29NO3 | Propantheline | S2,S6,S5,S1,S9,S8,S7,S10 |
| 10.65 | 12.57 | 216.20 | -1.02 | C12H25NO2 | Heptanoylcholine | S2,S6,S5,S1,S9,S8,S7 |
| 10.66 | 10.66 | 492.30 | -0.68 | C20H41N7O5S | Glutathionylspermine | S2,S6,S5,S1,S9,S8,S7,S10 |
| 10.66 | 15.06 | 272.26 | -0.29 | C16H33NO2 | Undecanoylcholine | S2,S6,S1,S9,S8,S7,S10 |
| 10.67 | 11.35 | 563.21 | -3.70 | C24H30N6O10 | Asp-Trp-Asp-Gln | S2,S6,S1,S9,S8,S7,S10 |
| 10.68 | 15.36 | 408.31 | -0.35 | C24H41NO4 | Cassaidine | S2,S5,S1,S9,S8,S7,S10 |
| 10.70 | 12.32 | 242.19 | 0.28 | C17H23N | N-Methyl morphinan | S2,S6,S5,S1,S9,S8,S7,S10 |
| 10.70 | 6.24 | 161.11 | -1.17 | C10H12N2 | Anatabine | S2,S6,S5,S1,S9,S8,S7,S10 |
| 10.71 | 14.42 | 412.31 | 1.39 | C23H41NO5 | 3-Hydroxyhexadecadienoylcarnitine | S2,S5,S1,S9,S8,S7,S10 |
| 10.71 | 6.63 | 300.09 | -3.58 | C10H13N5O6 | 8-Hydroxyguanosine | S2,S6,S5,S1,S9,S8,S7,S10 |
| 10.71 | 15.43 | 749.53 | -0.73 | C40H77O10P | [PG (16:0/18:1)] 1-Hexadecanoyl-2-(9Z-octadecenoyl)-sn-glycero-3-phospho-sn-glycerol | S6,S5 |
| 10.72 | 14.82 | 124.04 | -0.64 | C6H5NO2 | Nitrobenzene | S2,S6,S5,S1,S9,S8,S7,S10 |
| 10.72 | 15.43 | 415.35 | 3.27 | C23H46N2O4 | lyso-ornithine lipid | S2,S6,S5,S1,S9,S8,S7,S10 |
| 10.72 | 15.44 | 440.24 | 0.17 | C26H33NO5 | Militarinone B | S2,S6,S5,S1,S8,S7 |
| 10.73 | 14.94 | 141.05 | -3.07 | C7H8O3 | 2,3,5-Trihydroxytoluene | S2,S6,S5,S1,S9,S8,S7,S10 |
| 10.73 | 12.01 | 368.16 | 0.89 | C17H25N3O4S | Met-Phe-Ala | S2,S6,S5,S1,S9,S8,S7,S10 |
| 10.74 | 14.13 | 378.21 | -0.40 | C24H27NO3 | Cryptopleurine | S2,S6,S5,S1,S9,S8,S7,S10 |
| 10.75 | 14.21 | 379.21 | -1.46 | C21H30O6 | 6alpha-Hydroxycortisol | S6,S5,S1,S9,S8,S10 |
| 10.75 | 12.28 | 222.13 | -1.51 | C16H15N | Dizocilpine | S2,S6,S5,S1,S9,S8,S7,S10 |
| 10.76 | 14.23 | 242.21 | -1.83 | C14H27NO2 | [SP (14:0/2:0)] tetradecasphinga-4E,6E-dienine | S2,S6,S5,S1,S9,S8,S7,S10 |
| 10.77 | 10.77 | 303.07 | 3.62 | C8H18N2O6S2 | PIPES | S2,S6,S5,S1,S8,S7 |
| 10.79 | 11.54 | 384.16 | 3.40 | C17H25N3O5S | Met-Ala-Tyr | S2,S6,S5,S1,S9,S8,S7,S10 |
| 10.81 | 15.62 | 450.29 | -0.16 | C21H42N2O6P | [SP] N-(acyl)-sphing-4-enine-1-phosphoethanolamine | S2,S6,S5,S1,S9,S8,S7,S10 |
| 10.81 | 15.59 | 384.27 | -1.81 | C21H37NO5 | 3-Hydroxy-5, 8-tetradecadiencarnitine | S2,S6,S5,S1,S9,S8,S7,S10 |
| 10.81 | 15.12 | 314.27 | 0.30 | C18H35NO3 | (+)-Prosopinine | S2,S6,S5,S1,S9,S8,S7,S10 |
| 10.84 | 10.49 | 370.14 | 2.05 | C16H23N3O5S | Met-Gly-Tyr | S2,S6,S5,S1,S9,S8,S7,S10 |
| 10.85 | 15.51 | 317.17 | -0.57 | C19H24O4 | 1-Dehydro-15alpha-hydroxytestololactone | S2,S6,S5,S1,S9,S8,S7,S10 |
| 10.86 | 15.56 | 316.25 | -3.06 | C17H33NO4 | [FA (10:0)] O-decanoyl-R-carnitine | S2,S6,S5,S1,S9,S8,S7 |
| 10.87 | 11.74 | 534.23 | 0.07 | C20H35N7O8S | Gln-Met-Gln-Gln | S2,S6,S5,S8,S7,S10 |
| 10.87 | 15.75 | 498.28 | 2.53 | C22H44NO9P | [PS (16:0)] 1-hexadecanoyl-sn-glycero-3-phosphoserine | S2,S6,S5,S1,S9,S8,S7,S10 |
| 10.89 | 14.77 | 316.28 | 0.28 | C18H37NO3 | Dehydrophytosphingosine | S2,S6,S5,S1,S9,S8,S7,S10 |
| 10.89 | 15.66 | 294.21 | 2.29 | C17H27NO3 | (+/-)-5-[(tert-Butylamino)-2'-hydroxypropoxy]-1,2,3,4-tetrahydro-1- naphthol | S2,S6,S5,S1,S9,S8,S7,S10 |
| 10.90 | 15.79 | 453.24 | -0.01 | C27H28N6O | Hoechst 33342 | S2,S6,S1,S9,S8,S7,S10 |
| 10.92 | 14.45 | 256.17 | 0.52 | C17H21NO | Diphenhydramine | S2,S6,S5,S1,S9,S8,S7 |
| 10.92 | 12.10 | 350.14 | 1.60 | C21H19NO4 | Fagaronine | S2,S6,S5,S9,S8,S7,S10 |
| 10.92 | 13.73 | 266.21 | -0.18 | C16H27NO2 | [SP hydroxy,methyl(10:0/2:0/2:0/2:0)] 6S-((deca-1E,3E,5E-trienyl)-2R-(hydroxymethyl)-piperidin-3R-ol | S2,S6,S5,S1,S9,S8,S7,S10 |
| 10.93 | 15.59 | 311.18 | -3.18 | C17H26O5 | Botrydial | S2,S6,S5,S1,S8,S7,S10 |
| 10.93 | 9.47 | 208.17 | -0.34 | C13H21NO | Luciduline | S2,S6,S5,S1,S9,S8,S7,S10 |
| 10.94 | 13.92 | 238.16 | -0.94 | C17H19N | N-Methyl-(R,S)-tetrahydrobenzylisoquinoline | S2,S6,S5,S1,S9,S8,S7 |
| 10.95 | 15.23 | 136.08 | -1.69 | C8H9NO | 2-Phenylacetamide | S2,S6,S5,S1,S9,S8,S7,S10 |
| 10.96 | 11.92 | 492.19 | 0.99 | C17H29N7O8S | Asn-Met-Asn-Asn | S2,S8,S7,S10 |
| 10.96 | 13.30 | 236.14 | -1.45 | C17H17N | (S)-7,8,13,14-Tetrahydroprotoberberine | S2,S6,S5,S1,S9,S7,S10 |
| 10.96 | 14.61 | 298.27 | 1.18 | C18H35NO2 | [SP hydroxy,methyl(8:2/10:0)] 5S-(5S-hydroxyoctyl)-2S-methyldecahydroquinolin-3R-ol | S2,S6,S5,S1,S9,S8,S7,S10 |
| 10.97 | 11.94 | 511.29 | -0.52 | C28H38N4O5 | Phe-Phe-Val-Val | S2,S8,S7 |
| 11.00 | 12.28 | 246.15 | -0.05 | C15H19NO2 | Benzoyltropein | S2,S6,S5,S1,S9,S8,S7,S10 |
| 11.00 | 11.00 | 480.30 | -0.10 | C26H41NO7 | Delcorine | S2,S6,S5,S1,S8,S7,S10 |
| 11.00 | 12.76 | 336.22 | 0.81 | C19H29NO4 | Ankorine | S2,S6,S5,S1,S9,S8,S7,S10 |
| 11.04 | 11.29 | 269.13 | 1.02 | C16H16N2O2 | paspalate | S2,S6,S5,S1,S9,S8,S7,S10 |
| 11.05 | 13.60 | 260.19 | -2.73 | C13H25NO4 | [FA (6:0)] O-hexanoyl-R-carnitine | S6,S5,S1,S9 |
| 11.06 | 11.95 | 189.14 | -0.84 | C12H16N2 | N,N-Dimethyltryptamine | S2,S6,S5,S1,S9,S8,S7,S10 |
| 11.06 | 15.13 | 302.23 | -1.00 | C16H31NO4 | 2-6dimethylheptanoylcarnitine | S2,S6,S1,S9,S8,S7 |
| 11.06 | 9.23 | 152.11 | -0.86 | C9H13NO | D-Cathine | S2,S6,S5,S1,S9,S8,S10 |
| 11.07 | 13.65 | 272.16 | 0.02 | C17H21NO2 | Apoatropine | S2,S6,S5,S1,S9,S8,S7,S10 |
| 11.08 | 10.43 | 128.07 | 0.02 | C6H9NO2 | 2,3,4,5-Tetrahydropyridine-2-carboxylate | S2,S6,S5,S1,S9,S8,S7,S10 |
| 11.08 | 14.70 | 279.14 | 0.75 | C11H22N2O4S | Pantetheine | S2,S6,S5,S1,S9,S8,S7,S10 |
| 11.09 | 7.02 | 159.08 | 4.03 | C6H10N2O3 | 4-Methylene-L-glutamine | S6,S9 |
| 11.09 | 12.19 | 541.30 | -2.64 | C29H40N4O6 | Ile-Phe-Val-Tyr | S2,S6,S5,S1,S9,S8,S7,S10 |
| 11.10 | 10.31 | 352.12 | 2.02 | C20H17NO5 | Berberastine | S2,S6,S9,S8,S7 |
| 11.10 | 6.20 | 418.17 | -1.29 | C14H23N7O8 | Asn-Asn-Asn-Gly | S2,S6,S5,S1,S9,S8,S7,S10 |
| 11.10 | 11.10 | 426.23 | -2.33 | C18H31N7O3S | S-adenosyl-1,8-diamino-3-thiooctane | S2,S6,S5,S1,S9,S8,S7,S10 |
| 11.11 | 12.15 | 358.16 | -0.78 | C20H23NO5 | deacetylcolchicine | S2,S6,S5,S1,S9,S8,S7,S10 |
| 11.11 | 15.74 | 204.17 | -0.63 | C14H21N | N-(Cyclohexylmethyl)-N-methylbenzenamine | S2,S6,S5,S1,S9,S8,S7,S10 |
| 11.11 | 12.22 | 443.32 | -0.11 | C22H42N4O5 | Ile-Leu-Val-Val | S2,S6,S5,S1,S9,S8,S7,S10 |
| 11.13 | 12.25 | 527.29 | 1.41 | C28H38N4O6 | Ile-Phe-Phe-Thr | S2,S6,S5,S1,S9,S8,S7 |
| 11.13 | 15.89 | 331.15 | -0.80 | C19H22O5 | Gibberellin A7 | S2,S6,S5,S1,S9,S8,S7,S10 |
| 11.14 | 11.05 | 229.12 | 2.04 | C10H16N2O4 | (S)-ATPA | S2,S6,S5,S1,S9,S8,S7,S10 |
| 11.14 | 8.17 | 138.09 | -0.84 | C8H11NO | Phenylethanolamine | S2,S6,S5,S1,S8,S7,S10 |
| 11.14 | 10.84 | 270.15 | -0.61 | C17H19NO2 | Strobamine | S2,S6,S5,S1,S9,S8,S7,S10 |
| 11.17 | 11.17 | 468.30 | 0.71 | C25H41NO7 | Browniine | S2,S6,S1,S8,S7,S10 |
| 11.18 | 13.36 | 394.16 | -0.62 | C23H23NO5 | Sanguilutine | S2,S6,S5,S1,S9,S8,S7,S10 |
| 11.18 | 13.64 | 174.15 | -0.67 | C9H19NO2 | [FA amino(9:0)] 9-amino-nonanoic acid | S2,S6,S5,S1,S9,S8,S10 |
| 11.22 | 16.19 | 118.07 | 0.69 | C8H7N | Phenylacetonitrile | S2,S6,S5,S1,S9,S8,S7,S10 |
| 11.23 | 14.81 | 147.09 | -0.85 | C9H10N2 | Dimethylbenzimidazole | S2,S6,S5,S1,S9,S8,S7,S10 |
| 11.24 | 10.72 | 258.15 | 0.23 | C16H19NO2 | (+)-Elaeocarpine | S2,S6,S5,S1,S9,S8,S7,S10 |
| 11.25 | 12.48 | 244.15 | -0.13 | C12H21NO4 | Tiglylcarnitine | S2,S6,S5,S1,S9,S8,S7 |
| 11.27 | 14.83 | 257.10 | 3.56 | C12H16O6 | Phenyl beta-D-glucopyranoside | S2,S6,S5,S1,S9,S8,S7,S10 |
| 11.29 | 14.26 | 291.10 | 2.95 | C14H14N2O5 | N2-Malonyl-D-tryptophan | S2,S6,S5,S1,S9,S8,S7,S10 |
| 11.29 | 10.81 | 298.14 | 1.17 | C18H19NO3 | 7,8-Didehydro-4,5-epoxy-3-methoxy-17-methoxy-(5_)-morphinan-6-one | S2,S6,S5,S1,S9,S8,S7 |
| 11.30 | 12.64 | 336.12 | 2.72 | C20H17NO4 | Berberine | S2,S6,S5,S1,S9,S8,S7,S10 |
| 11.31 | 15.53 | 112.04 | 0.53 | C5H5NO2 | Pyrrole-2-carboxylate | S2,S6,S5,S1,S9,S8,S7,S10 |
| 11.31 | 15.11 | 326.14 | 1.15 | C19H19NO4 | (S)-Cheilanthifoline | S2,S6,S5,S1,S9,S8,S7,S10 |
| 11.31 | 15.40 | 202.18 | -1.91 | C11H23NO2 | [FA amino(11:0)] 11-amino-undecanoic acid | S2,S6,S5,S1,S9,S8,S7,S10 |
| 11.32 | 10.03 | 336.18 | -0.33 | C18H25NO5 | Senecionine | S2,S6,S5,S1,S9,S8,S7,S10 |
| 11.33 | 8.39 | 338.20 | -0.56 | C18H27NO5 | Nemorensine | S2,S6,S5,S1,S9,S8,S7,S10 |
| 11.38 | 12.14 | 290.18 | 2.49 | C17H23NO3 | L-Hyoscyamine | S2,S6,S5,S1,S9,S8,S7,S10 |
| 11.40 | 12.79 | 412.14 | 0.75 | C15H29N3O4S3 | Met-Met-Met | S2,S6,S5,S1,S9,S8,S7,S10 |
| 11.40 | 10.26 | 332.19 | 2.24 | C19H25NO4 | Erythratidine | S2,S6,S5,S1,S9,S8,S7,S10 |
| 11.40 | 16.31 | 170.10 | -2.47 | C12H11N | 4-Aminobiphenyl | S2,S6,S5,S1,S9,S8,S7,S10 |
| 11.40 | 14.04 | 250.18 | -3.91 | C15H23NO2 | Lophocerine | S2,S6,S5,S1,S9,S8,S7,S10 |
| 11.40 | 9.71 | 242.18 | -0.49 | C13H23NO3 | Valeroidine | S6,S5,S1,S9,S8,S7,S10 |
| 11.43 | 14.82 | 334.20 | 1.56 | C19H27NO4 | alpha-Eucaine | S2,S6,S5,S1,S9,S8,S7,S10 |
| 11.45 | 15.02 | 188.16 | -1.25 | C10H21NO2 | [FA amino(10:0)] 10-amino-decanoic acid | S2,S6,S5,S1,S9,S8 |
| 11.46 | 13.95 | 268.13 | -0.69 | C17H17NO2 | (-)-Caaverine | S2,S6,S5,S1,S9,S8,S7,S10 |
| 11.47 | 8.28 | 143.08 | 3.30 | C6H10N2O2 | Ectoine | S2,S6,S5,S1,S9 |
| 11.47 | 11.47 | 205.06 | 0.29 | C19H20O10 | Khellol glucoside | S2,S6,S5,S1,S9,S8,S7,S10 |
| 11.47 | 11.47 | 398.16 | -1.36 | C22H23NO6 | Aureothin | S2,S6,S5,S1,S9,S8,S7,S10 |
| 11.48 | 13.28 | 180.07 | -0.85 | C9H9NO3 | 2,5,6-Trihydroxy-5,6-dihydroquinoline | S2,S6,S5,S1,S9,S8,S7,S10 |
| 11.49 | 11.93 | 222.15 | -0.35 | C13H19NO2 | (+-)-Carnegine | S2,S6,S5,S1,S9,S8,S7,S10 |
| 11.50 | 7.17 | 187.14 | 1.98 | C9H18N2O2 | N-(3-acetamidopropyl)-4-aminobutanal | S2,S6,S5,S1,S9,S8,S7 |
| 11.51 | 15.09 | 313.16 | 3.32 | C18H20N2O3 | Phe-Phe | S2,S6,S5,S1,S9,S8,S7,S10 |
| 11.51 | 12.57 | 175.12 | -1.53 | C11H14N2 | N-Methyltryptamine | S2,S6,S5,S1,S9,S8,S7,S10 |
| 11.51 | 13.35 | 394.19 | -0.97 | C20H27NO7 | Latifoline | S2,S6,S5,S1,S9,S8,S7,S10 |
| 11.52 | 15.86 | 276.12 | 0.57 | C15H17NO4 | Dubinidine | S2,S6,S5,S1,S9,S8,S7,S10 |
| 11.52 | 7.04 | 462.19 | -1.13 | C16H27N7O9 | Asn-Asn-Gln-Ser | S2,S6,S8,S7,S10 |
| 11.56 | 11.55 | 230.14 | -1.95 | C11H19NO4 | Butenylcarnitine | S2,S6,S5,S1,S9,S8,S7,S10 |
| 11.57 | 12.65 | 194.12 | -1.61 | C11H15NO2 | 3,4-Methylenedioxymethamphetamine | S2,S6,S5,S1,S9,S8,S7 |
| 11.58 | 16.53 | 194.10 | -0.96 | C14H11N | 2-Anthramine | S2,S6,S5,S1,S9,S8,S7,S10 |
| 11.59 | 10.09 | 324.16 | 1.37 | C15H21N3O5 | Ala-Ala-Tyr | S2,S6,S5,S1,S9,S7,S10 |
| 11.60 | 7.52 | 354.19 | 0.66 | C18H27NO6 | Rosmarinine | S2,S6,S5,S1,S9,S8,S7,S10 |
| 11.69 | 16.33 | 164.07 | -1.13 | C9H9NO2 | 3-Methyldioxyindole | S2,S6,S5,S1,S9,S8,S7,S10 |
| 11.70 | 16.13 | 211.07 | -0.94 | C9H10N2O4 | N-carbamoyl-p-hydroxy-D-phenylglycine | S2,S6,S5,S1,S9,S7,S10 |
| 11.71 | 13.50 | 310.16 | -0.44 | C16H23NO5 | Sinapoylcholine | S2,S6,S5,S1,S9,S8,S7,S10 |
| 11.73 | 16.30 | 204.07 | -1.60 | C11H9NO3 | Indolepyruvate | S2,S6,S5,S1,S9,S8,S7,S10 |
| 11.73 | 8.46 | 402.16 | -3.70 | C15H23N5O8 | Asn-Asp-Gly-Pro | S2,S6,S5,S1,S9,S8,S7,S10 |
| 11.73 | 15.28 | 130.16 | 0.13 | C8H19N | Octylamine | S2,S6,S5,S1,S9,S8,S7,S10 |
| 11.74 | 8.55 | 341.19 | -0.52 | C20H24N2O3 | 3-Hydroxyquinine | S2,S6,S5,S1,S9,S8,S7,S10 |
| 11.74 | 14.42 | 302.07 | 2.40 | C10H12ClN5O4 | 5'-chloroformycin | S2,S6,S9,S8,S7 |
| 11.74 | 12.25 | 276.20 | 0.50 | C17H25NO2 | Lycoflexine | S2,S6,S5,S1,S9,S8,S7,S10 |
| 11.75 | 14.39 | 245.19 | 2.29 | C12H24N2O3 | Leucyl-leucine | S2,S6,S1,S9,S8,S7,S10 |
| 11.75 | 16.97 | 254.10 | -0.97 | C12H15NO5 | N-Acetylvanilalanine | S2,S6,S5,S1,S9,S8,S7,S10 |
| 11.76 | 11.34 | 174.09 | 0.20 | C6H11N3O3 | 5-Guanidino-2-oxopentanoate | S2 |
| 11.77 | 11.77 | 437.11 | 2.69 | C20H20O11 | Irisxanthone | S2,S6,S5,S1,S9,S8,S7 |
| 11.78 | 15.03 | 176.14 | -0.13 | C12H17N | Nigrifactin | S2,S6,S5,S1,S9,S8,S7,S10 |
| 11.79 | 13.37 | 224.16 | 0.00 | C13H21NO2 | Tigloidine | S2,S6,S5,S1,S9,S8,S7,S10 |
| 11.80 | 12.07 | 196.06 | -0.30 | C9H9NO4 | Dopaquinone | S2,S6,S5,S1,S9,S8,S7,S10 |
| 11.81 | 8.62 | 268.10 | 3.26 | C20H30N4O13 | Glu-Glu-Glu-Glu | S2,S6,S5,S1,S9,S8,S7,S10 |
| 11.82 | 15.18 | 279.17 | 1.07 | C15H22N2O3 | Leu-Phe | S2,S6,S7 |
| 11.84 | 15.53 | 220.17 | 0.59 | C14H21NO | Fabianine | S2,S6,S5,S1,S9,S8,S7,S10 |
| 11.84 | 14.58 | 124.08 | -0.31 | C7H9NO | 2-amino-4-methylphenol | S2,S6,S5,S1,S9,S8,S7,S10 |
| 11.85 | 12.30 | 161.11 | -0.37 | C10H12N2 | Tryptamine | S2,S6,S5,S1,S9,S8,S7,S10 |
| 11.88 | 15.07 | 216.12 | -2.20 | C10H17NO4 | 2-Amino-9,10-epoxy-8-oxodecanoic acid | S2,S6,S5,S1,S9,S8,S7,S10 |
| 11.88 | 16.21 | 274.11 | 0.22 | C15H15NO4 | L-Thyronine | S2,S6,S5,S1,S9,S8,S7,S10 |
| 11.88 | 16.20 | 266.07 | 3.00 | C12H11NO6 | nitecapone | S2,S6,S9,S8,S7,S10 |
| 11.89 | 17.04 | 218.08 | 0.84 | C12H11NO3 | 3-Methylindolepyruvate | S2,S6,S5,S1,S9,S8,S7,S10 |
| 11.90 | 9.82 | 268.15 | -0.04 | C14H21NO4 | Codonopsine | S2,S6,S5,S1,S9,S8,S7,S10 |
| 11.90 | 16.53 | 317.14 | 3.70 | C14H16N6O3 | tetrahydropteroate | S6,S5,S1,S9,S8,S7,S10 |
| 11.91 | 8.41 | 346.13 | 0.74 | C18H19NO6 | Candimine | S2,S6,S5,S1,S9,S8,S7,S10 |
| 11.92 | 11.65 | 314.20 | -2.03 | C16H27NO5 | Heliotrine | S2,S6,S5,S1,S9,S8,S7,S10 |
| 11.92 | 12.89 | 166.12 | -0.60 | C10H15NO | Hordenine | S2,S6,S5,S1,S9,S8,S7,S10 |
| 11.94 | 14.75 | 248.11 | -0.26 | C10H17NO6 | Linamarin | S2,S6,S5,S1,S9,S8,S7,S10 |
| 11.95 | 10.00 | 174.09 | -1.69 | C11H11NO | Echinorine | S6,S5,S1,S9,S8,S7,S10 |
| 11.95 | 13.91 | 509.18 | -0.18 | C21H28N6O7S | Asn-Trp-Cys-Ser | S2,S6,S8,S7,S10 |
| 11.96 | 17.22 | 321.12 | 3.61 | C19H16N2O3 | Alangimarine | S2,S6,S5,S1,S9,S8,S7,S10 |
| 11.97 | 16.90 | 246.08 | -0.53 | C13H11NO4 | Haplopine | S2,S6,S5,S1,S9,S8,S10 |
| 11.97 | 9.40 | 195.09 | -2.48 | C13H10N2 | phenazine methosulfate | S2,S6,S5,S1,S9,S8,S7,S10 |
| 11.97 | 14.90 | 258.17 | -1.28 | C13H23NO4 | 2-Hexenoylcarnitine | S2,S6,S5,S1,S9,S8,S7,S10 |
| 11.98 | 10.12 | 269.11 | 0.26 | C12H16N2O5 | Ser-Tyr | S2,S6,S5,S1,S9,S8,S7,S10 |
| 12.02 | 15.88 | 130.07 | -1.55 | C9H7N | Isoquinoline | S2,S6,S1,S9,S8,S7,S10 |
| 12.02 | 16.97 | 190.11 | -1.29 | C8H15NO4 | 2 -(Butylamido)-4-hydroxybutanoic acid | S2,S6,S5,S1,S9,S8,S7,S10 |
| 12.04 | 14.08 | 214.06 | -0.49 | C14H26N4O5S3 | Ala-Met-Cys-Cys | S2,S6,S1,S7 |
| 12.06 | 17.25 | 183.09 | -1.99 | C12H10N2 | Harman | S2,S6,S5,S1,S9,S8,S7,S10 |
| 12.06 | 17.13 | 306.10 | -1.36 | C15H15NO6 | indol-3-ylmethyl-ascorbate | S2,S6,S5,S1,S9,S8,S7,S10 |
| 12.07 | 15.10 | 166.05 | -1.36 | C8H7NO3 | 4-Pyridoxolactone | S2,S6,S5,S1,S9,S8,S7,S10 |
| 12.07 | 10.45 | 168.10 | -0.65 | C9H13NO2 | 1-(4-Hydroxyphenyl)-2-(methylamino)ethanol | S2,S6,S5,S9,S8,S7,S10 |
| 12.08 | 17.04 | 287.09 | -0.14 | C16H14O5 | (S)-DNPA | S2,S6,S5,S1,S9,S8,S7,S10 |
| 12.08 | 16.21 | 267.10 | 2.42 | C12H14N2O5 | p-aminobenzoyl glutamate | S6,S5,S1,S9,S8,S7,S10 |
| 12.09 | 9.81 | 132.07 | 2.38 | C5H9NO3 | L-Glutamate 5-semialdehyde | S2,S6,S5,S1,S9,S8,S7,S10 |
| 12.09 | 12.89 | 196.10 | -0.70 | C10H13NO3 | L-Tyrosine methyl ester | S2,S6,S5,S1,S9,S8,S7,S10 |
| 12.12 | 14.95 | 138.06 | -1.01 | C7H7NO2 | 3-Pyridylaceticacid | S2,S6,S5,S1,S9,S8,S7,S10 |
| 12.13 | 15.75 | 260.19 | -2.56 | C13H25NO4 | Hexanoylcarnitine | S6,S5,S1,S9,S8,S7,S10 |
| 12.13 | 10.41 | 144.14 | -0.76 | C8H17NO | Butyro-betaine | S2,S6,S5,S1,S9,S8,S7,S10 |
| 12.14 | 14.81 | 181.06 | -3.55 | C8H8N2O3 | Nicotinurate | S2,S6,S5,S1,S9,S10 |
| 12.14 | 11.59 | 283.13 | 2.76 | C13H18N2O5 | Thr-Tyr | S2,S6,S5,S1,S9,S8,S7,S10 |
| 12.17 | 17.54 | 123.04 | -0.30 | C7H6O2 | Tropolone | S2,S6,S5,S1,S9,S8,S7,S10 |
| 12.18 | 17.20 | 242.07 | -0.40 | C10H11NO6 | N-(2,3-Dihydroxybenzoyl)-L-serine | S2,S6,S8,S7,S10 |
| 12.19 | 16.47 | 220.06 | -2.49 | C11H9NO4 | 5-Hydroxyindolepyruvate | S2,S6,S5,S1,S9,S8,S7,S10 |
| 12.19 | 17.48 | 214.07 | -1.11 | C9H11NO5 | N,N-Dihydroxy-L-tyrosine | S2,S6,S5,S1,S9,S8,S7,S10 |
| 12.19 | 17.26 | 286.20 | 0.62 | C15H27NO4 | 2-Octenoylcarnitine | S2,S6,S1,S9,S8,S7,S10 |
| 12.19 | 14.39 | 481.15 | -1.95 | C16H28N6O7S2 | Asn-Met-Asn-Cys | S2,S6,S5,S1,S9,S8,S7 |
| 12.20 | 14.94 | 272.13 | 0.41 | C16H17NO3 | (S)-Norcoclaurine | S2,S6,S5,S1,S9,S8,S7,S10 |
| 12.21 | 17.05 | 176.11 | -1.25 | C11H13NO | 4-trans-(N,N-dimethylamino)cinnamaldehyde | S2,S6,S5,S1,S9,S8,S7,S10 |
| 12.21 | 16.29 | 256.12 | -0.12 | C12H17NO5 | N-D-Glucosylarylamine | S6,S5,S1,S9,S8,S7,S10 |
| 12.21 | 16.38 | 169.08 | -2.22 | C11H8N2 | beta-Carboline | S2,S6,S5,S1,S9,S8,S7,S10 |
| 12.24 | 9.63 | 206.12 | -0.40 | C12H15NO2 | Bellendine | S2,S6,S5,S1,S9,S8,S7,S10 |
| 12.25 | 14.86 | 400.14 | 2.62 | C21H21NO7 | Narcotoline | S2,S6,S5,S1,S8,S7,S10 |
| 12.28 | 17.62 | 115.04 | 0.35 | C5H6O3 | 2-Hydroxy-2,4-pentadienoate | S2,S6,S5,S1,S9,S8,S7,S10 |
| 12.28 | 14.57 | 250.07 | -0.64 | C17H30N4O7S3 | Asp-Met-Met-Cys | S2,S6,S5,S1,S9,S8,S7,S10 |
| 12.30 | 14.83 | 246.17 | -1.03 | C12H23NO4 | 2-Methylbutyroylcarnitine | S2,S6,S5,S1,S9,S8,S7,S10 |
| 12.31 | 17.70 | 213.10 | -1.10 | C13H12N2O | Harmine | S2,S6,S5,S1,S9,S8,S7,S10 |
| 12.33 | 17.11 | 85.03 | 3.71 | C4H4O2 | 3-Butynoate | S2,S6,S5,S1,S9,S8,S7,S10 |
| 12.33 | 16.50 | 228.05 | -0.39 | C9H9NO6 | 5-(2'-Carboxyethyl)-4,6-dihydroxypicolinate | S2,S6,S5,S1,S9,S8,S7 |
| 12.33 | 11.82 | 130.09 | 2.27 | C6H11NO2 | L-Pipecolate | S2,S6,S5,S1,S9,S8,S7,S10 |
| 12.34 | 16.62 | 130.05 | -0.33 | C5H7NO3 | 1-Pyrroline-4-hydroxy-2-carboxylate | S2,S6,S1,S9,S8,S7,S10 |
| 12.34 | 11.59 | 338.20 | 0.18 | C18H27NO5 | Platyphylline | S2,S6,S5,S1,S9,S8,S7,S10 |
| 12.36 | 13.80 | 174.13 | -1.78 | C12H15N | 1-Methyl-4-phenyl-1,2,3,6-tetrahydropyridine | S2,S6,S5,S1,S9,S8,S7,S10 |
| 12.37 | 13.11 | 227.07 | 0.08 | C9H10N2O5 | 3-Nitrotyrosine | S2,S6,S5,S1,S9,S8 |
| 12.39 | 13.67 | 263.14 | 0.06 | C14H18N2O3 | Phe-Pro | S2,S6,S5,S1,S9,S8,S7,S10 |
| 12.39 | 12.39 | 321.06 | 1.16 | C8H17O11P | octulose 8-phosphate | S2,S6,S5,S1,S9,S8,S7,S10 |
| 12.40 | 13.51 | 152.11 | -0.63 | C9H13NO | N-Methyltyramine | S2,S6,S5,S1,S9,S8,S7,S10 |
| 12.41 | 9.40 | 187.07 | 1.20 | C7H10N2O4 | (S)-AMPA | S2,S6,S1,S9,S10 |
| 12.41 | 15.57 | 265.16 | 2.10 | C14H20N2O3 | Phe-Val | S6,S5,S1,S9,S8,S7,S10 |
| 12.41 | 16.88 | 174.08 | -2.24 | C7H11NO4 | N-Acetyl-L-glutamate 5-semialdehyde | S2,S6,S5,S1,S9,S8,S7,S10 |
| 12.42 | 15.88 | 168.03 | -1.09 | C7H5NO4 | Pyridine-2,3-dicarboxylate | S2,S6,S5,S1,S9,S8,S7,S10 |
| 12.42 | 15.77 | 199.09 | -0.25 | C12H10N2O | Harmalol | S2,S6,S5,S1,S9,S8,S7,S10 |
| 12.43 | 14.84 | 244.15 | -0.65 | C12H21NO4 | Tiglylcarnitine | S2,S6,S5,S1,S9,S8,S7,S10 |
| 12.44 | 15.63 | 176.07 | -1.53 | C10H9NO2 | 3-Indoleglycolaldehyde | S2,S6,S5,S1,S9,S8,S7,S10 |
| 12.46 | 14.46 | 328.22 | 1.32 | C16H29N3O4 | Leu-Val-Pro | S2,S6,S5,S1,S7,S10 |
| 12.46 | 14.29 | 290.18 | 1.93 | C17H23NO3 | Atropine | S2,S6,S5,S1,S9,S8,S7,S10 |
| 12.47 | 10.29 | 143.08 | 3.71 | C6H10N2O2 | Ectoine | S2,S6,S5,S1,S9,S8,S7,S10 |
| 12.50 | 12.48 | 184.06 | -1.26 | C8H9NO4 | 3-Hydroxy-4-hydroxymethyl-2-methylpyridine-5-carboxylate | S2,S6,S5,S1,S9,S8,S7,S10 |
| 12.51 | 11.13 | 218.12 | -1.30 | C13H15NO2 | Securinine | S2,S6,S5,S1,S9,S8,S7,S10 |
| 12.53 | 16.21 | 347.12 | 2.88 | C17H18N2O6 | Miraxanthin-V | S2,S6,S5,S1,S9,S8,S7,S10 |
| 12.53 | 11.60 | 180.10 | -0.72 | C10H13NO2 | (-)-Salsolinol | S2,S6,S5,S1,S9,S8,S7,S10 |
| 12.55 | 16.17 | 146.12 | 0.26 | C7H15NO2 | [FA amino(7:0)] 2R-aminoheptanoic acid | S2,S6,S5,S1,S9,S8,S7,S10 |
| 12.56 | 13.55 | 214.11 | -2.63 | C10H15NO4 | Kainic acid | S2,S6,S5,S1,S9,S8,S7,S10 |
| 12.57 | 13.45 | 182.08 | -1.23 | C9H11NO3 | 3-Amino-3-(4-hydroxyphenyl)propanoate | S2,S6,S5,S1,S9,S8,S7,S10 |
| 12.61 | 13.42 | 166.09 | -0.86 | C9H11NO2 | L-Phenylalanine | S2,S6,S5,S1,S9,S8,S7,S10 |
| 12.61 | 15.13 | 231.17 | 1.23 | C11H22N2O3 | Leu-Val | S2,S6,S5,S1,S9,S8,S7,S10 |
| 12.62 | 12.10 | 300.19 | 3.91 | C14H25N3O4 | Leu-Ala-Pro | S2,S5,S1 |
| 12.64 | 7.69 | 314.12 | 3.15 | C14H19NO7 | Tyramineglucuronide | S2,S6,S5,S1,S9,S8,S7,S10 |
| 12.66 | 18.07 | 169.05 | -0.38 | C8H8O4 | 3,4-Dihydroxymandelaldehyde | S2,S6,S5,S1,S9,S8,S7,S10 |
| 12.68 | 14.76 | 176.09 | -1.63 | C7H13NO4 | alpha-aminopimelate | S2,S6,S5,S1,S9,S8,S7,S10 |
| 12.68 | 18.21 | 161.07 | 3.29 | C17H20O6 | Arctolide | S2,S6,S5,S1,S9,S8,S7,S10 |
| 12.68 | 12.97 | 138.09 | -0.77 | C8H11NO | Tyramine | S2,S6,S5,S1,S9,S8,S7,S10 |
| 12.68 | 9.98 | 184.13 | -0.86 | C10H17NO2 | Acetylpseudotropine | S2,S6,S5,S1,S9,S8,S7,S10 |
| 12.69 | 14.12 | 217.16 | 2.45 | C10H20N2O3 | Val-Val | S2,S6,S5,S1 |
| 12.70 | 16.64 | 133.08 | -0.90 | C8H8N2 | Indoleamine | S2,S6,S5,S1,S9,S8,S7,S10 |
| 12.71 | 17.92 | 144.08 | -1.25 | C10H9N | 2-Naphthylamine | S2,S6,S5,S1,S9,S8,S7,S10 |
| 12.73 | 16.84 | 129.07 | 0.45 | C5H8N2O2 | 5,6-Dihydrothymine | S2,S6,S5,S1,S9,S8,S10 |
| 12.75 | 18.11 | 148.11 | -0.94 | C10H13N | Actinidine | S2,S6,S5,S1,S9,S8,S7,S10 |
| 12.76 | 18.22 | 139.05 | -1.48 | C6H6N2O2 | 4-Nitroaniline | S2,S6,S5,S1,S9,S8,S7,S10 |
| 12.77 | 13.29 | 205.10 | 0.33 | C11H12N2O2 | L-Tryptophan | S2,S6,S5,S1,S9,S8,S7,S10 |
| 12.78 | 12.10 | 228.09 | -1.42 | C10H13NO5 | L-Arogenate | S2,S6,S5,S1,S9,S8,S7,S10 |
| 12.81 | 17.90 | 133.05 | 1.36 | C5H8O4 | 2-Acetolactate | S2,S6,S5,S1,S9,S8,S7,S10 |
| 12.82 | 17.06 | 162.05 | -1.34 | C9H7NO2 | Quinoline-3,4-diol | S2,S6,S5,S1,S9,S8,S7,S10 |
| 12.82 | 15.01 | 128.14 | 0.18 | C8H17N | Coniine | S2,S6,S5,S1,S9,S8,S7,S10 |
| 12.85 | 10.02 | 197.09 | -1.37 | C18H24N4O6 | Asn-Pro-Tyr | S2,S6,S5,S1,S9,S8,S7,S10 |
| 12.90 | 14.92 | 196.06 | -1.56 | C9H9NO4 | 2-Carboxy-2,3-dihydro-5,6-dihydroxyindole | S2,S6,S5,S1,S9,S8,S7,S10 |
| 12.96 | 13.95 | 150.09 | -2.05 | C9H11NO | D-Cathinone | S2,S6,S5,S1,S9,S8,S7,S10 |
| 12.98 | 17.76 | 212.06 | -2.33 | C9H9NO5 | 5-(2'-Formylethyl)-4,6-dihydroxypicolinate | S2,S6,S5,S1,S9,S8,S7,S10 |
| 12.98 | 14.20 | 150.10 | -2.63 | C8H11N3 | benzylguanidine | S2,S6,S5,S1,S9,S8,S7,S10 |
| 13.02 | 11.03 | 432.17 | -3.19 | C16H25N5O9 | Asn-Asp-Pro-Ser | S2,S6,S5,S8,S7,S10 |
| 13.02 | 14.17 | 374.16 | 0.17 | C20H23NO6 | 1,2-O-Diacetylzephyranthine | S2,S6,S5,S1,S9,S8,S7,S10 |
| 13.02 | 10.20 | 140.07 | -0.97 | C7H9NO2 | Gabaculine | S2,S6,S5,S1,S9,S8,S7,S10 |
| 13.04 | 18.73 | 146.06 | -1.28 | C9H7NO | 8-hydroxyquinoline | S2,S6,S5,S1,S9,S8,S7 |
| 13.06 | 16.79 | 200.06 | -0.45 | C8H9NO5 | Clavulanic acid | S2,S6,S5,S1,S9,S8,S7,S10 |
| 13.07 | 14.03 | 178.09 | -0.70 | C10H11NO2 | 1,2-Dehydrosalsolinol | S2,S6,S5,S1,S9,S8,S7,S10 |
| 13.10 | 18.61 | 130.07 | -1.46 | C9H7N | Quinoline | S2,S6,S5,S1,S9,S8,S7,S10 |
| 13.15 | 18.82 | 150.08 | -0.47 | C5H11NO4 | N,N-dihydroxyvaline | S2,S6,S5,S1,S9,S8,S7,S10 |
| 13.16 | 17.28 | 170.04 | -0.30 | C7H7NO4 | 3,5-Dihydroxyanthranilate | S2,S6,S1,S8,S7,S10 |
| 13.19 | 14.74 | 178.12 | -0.66 | C11H15NO | Phenmetrazine | S2,S6,S5,S1,S9,S8,S7,S10 |
| 13.25 | 12.60 | 174.09 | -0.87 | C11H11NO | Echinorine | S2,S6,S5,S1,S9,S7,S10 |
| 13.26 | 17.11 | 115.05 | 2.26 | C4H6N2O2 | 5,6-Dihydrouracil | S2,S1,S9 |
| 13.34 | 18.22 | 173.06 | -2.32 | C6H8N2O4 | Hydantoin-5-propionate | S2,S6,S5,S8,S7,S10 |
| 13.35 | 19.16 | 206.05 | 3.76 | C10H7NO4 | Xanthurenic acid | S2,S6,S5,S1,S9,S8,S7,S10 |
| 13.37 | 14.04 | 131.09 | ########## | C6H13NO2 | L-Leucine | S2,S6,S5,S1,S9,S8,S7,S10 |
| 13.39 | 15.32 | 353.06 | -3.53 | C12H17O10P | Arbutin 6-phosphate | S2,S6,S5,S7,S10 |
| 13.40 | 14.89 | 164.09 | -2.19 | C7H9N5 | 1-ethyladenine | S2,S6,S5,S1,S9,S8,S7,S10 |
| 13.41 | 18.64 | 115.04 | 1.01 | C5H6O3 | norfuraneol | S2,S6,S5,S1,S9,S8,S7,S10 |
| 13.42 | 14.66 | 168.10 | -1.21 | C9H13NO2 | 3-Methoxytyramine | S2,S6,S5,S1,S9,S8,S7,S10 |
| 13.43 | 15.38 | 174.12 | -0.96 | C7H15N3O2 | &epsilon;-guanidinocaproate | S2 |
| 13.44 | 13.44 | 490.18 | -0.80 | C14H30N6O11P | O-1,4-alpha-L-Dihydrostreptosyl-streptidine 6-phosphate | S2,S6,S5,S8,S7,S10 |
| 13.45 | 13.45 | 321.06 | 1.24 | C8H17O11P | octulose 8-phosphate | S2,S6,S5,S1,S9,S8,S7,S10 |
| 13.46 | 13.04 | 215.14 | 2.06 | C10H18N2O3 | Val-Pro | S2,S6,S5,S1,S9,S8,S7,S10 |
| 13.48 | 18.89 | 137.11 | -1.56 | C8H12N2 | N,N-Dimethyl-1,4-phenylenediamine | S2,S6,S5,S1,S9,S8,S7,S10 |
| 13.48 | 16.97 | 489.25 | 1.97 | C23H32N6O6 | Ala-Trp-Val-Asn | S2,S6,S5,S8,S7,S10 |
| 13.65 | 16.34 | 198.08 | -0.94 | C9H11NO4 | 3,4-Dihydroxy-L-phenylalanine | S2,S6,S5,S1,S9,S8,S7,S10 |
| 13.68 | 17.36 | 471.35 | -1.16 | C24H46N4O5 | Leu-Leu-Leu-Leu | S2,S5,S10 |
| 13.70 | 11.11 | 203.07 | 2.13 | C7H10N2O5 | N3-fumaramoyl-L-2,3-diaminopropanoate | S2,S6,S5,S1,S9,S8,S7,S10 |
| 13.75 | 16.22 | 154.05 | -2.02 | C7H7NO3 | 3-Hydroxy-2-methylpyridine-5-carboxylate | S2,S6,S5,S1,S9,S8,S7,S10 |
| 13.75 | 17.94 | 190.11 | -1.66 | C8H15NO4 | (2S)-2-{[1-(R)-Carboxyethyl]amino}pentanoate | S6,S5,S1,S9,S8,S7,S10 |
| 13.76 | 9.83 | 187.11 | 1.89 | C8H14N2O3 | Ala-Pro | S2,S6,S5,S1,S9,S8 |
| 13.79 | 12.87 | 184.10 | -1.68 | C9H13NO3 | L-Adrenaline | S6,S5,S1,S9,S8,S7,S10 |
| 13.84 | 16.10 | 122.10 | -0.27 | C8H11N | Phenethylamine | S2,S6,S5,S1,S9,S8,S7,S10 |
| 13.89 | 14.52 | 156.14 | -0.86 | C9H17NO | N-Methylpelletierine | S6,S5,S1,S9,S8,S7,S10 |
| 13.91 | 16.19 | 164.11 | -1.08 | C10H13NO | (R)-2-Methylimino-1-phenylpropan-1-ol | S2,S6,S5,S1,S9,S8,S7,S10 |
| 13.92 | 16.89 | 153.10 | -1.24 | C8H12N2O | 4-(hydroxylamino)-N,N-dimethylaniline | S2,S6,S5,S1,S9,S8,S7,S10 |
| 13.93 | 18.87 | 126.06 | 0.01 | C6H7NO2 | Aminohydroquinone | S2,S6,S5,S1,S9,S8,S7,S10 |
| 13.94 | 13.25 | 142.12 | -2.84 | C8H15NO | Hygrine | S2,S6,S5,S1,S9,S8,S7,S10 |
| 13.98 | 13.34 | 132.10 | -0.83 | C6H13NO2 | L-Isoleucine | S2,S6,S5,S1,S9,S8,S7,S10 |
| 14.00 | 17.62 | 125.07 | -0.22 | C6H8N2O | Methylimidazole acetaldehyde | S2,S6,S5,S1,S9,S8,S7,S10 |
| 14.01 | 9.33 | 241.16 | 1.00 | C12H20N2O3 | Slaframine | S2,S6,S5,S1,S9,S8,S7,S10 |
| 14.01 | 18.69 | 208.13 | -1.51 | C12H17NO2 | 3-(Dimethylamino)propyl benzoate | S6,S5,S1,S9,S8,S7,S10 |
| 14.01 | 15.52 | 130.16 | -0.35 | C8H19N | Tetraethylammonium | S6,S5,S1,S9,S8,S7,S10 |
| 14.05 | 19.96 | 256.12 | -1.58 | C12H17NO5 | N-D-Glucosylarylamine | S2,S6,S5,S1,S9,S8,S7,S10 |
| 14.05 | 18.09 | 285.11 | 3.33 | C24H32N4O12 | Tyr-Glu-Glu-Glu | S2,S6,S5,S1,S9,S8,S7,S10 |
| 14.05 | 17.87 | 175.02 | 1.91 | C6H6O6 | L-Dehydroascorbate | S2,S6,S7 |
| 14.05 | 18.73 | 124.08 | -0.29 | C7H9NO | 6-amino-m-cresol | S2,S6,S5,S1,S9,S8,S7,S10 |
| 14.06 | 12.23 | 230.19 | 0.32 | C11H23N3O2 | N1,N8-diacetylspermidine | S5 |
| 14.07 | 16.61 | 196.06 | -1.20 | C9H9NO4 | Dopaquinone | S2,S6,S5,S1,S9,S8,S7,S10 |
| 14.08 | 19.19 | 184.06 | -0.39 | C8H9NO4 | 4-Pyridoxate | S2,S6,S5,S1,S9,S8,S7,S10 |
| 14.10 | 14.21 | 152.07 | -1.34 | C8H9NO2 | Dopaminequinone | S2,S6,S5,S1,S9,S8,S7,S10 |
| 14.10 | 9.63 | 378.26 | 0.90 | C22H35NO4 | Karakoline | S2,S6,S5,S1,S9,S8,S7,S10 |
| 14.10 | 13.00 | 145.13 | -1.40 | C7H16N2O | N-Acetylcadaverine | S2,S6,S5,S1,S9,S8,S7,S10 |
| 14.27 | 13.37 | 146.12 | -1.10 | C7H15NO2 | 3-Dehydroxycarnitine | S2,S6,S5,S1,S9,S8,S7,S10 |
| 14.29 | 14.16 | 170.15 | -0.37 | C10H19NO | Nitramine | S2,S6,S5,S1,S9,S8,S7,S10 |
| 14.33 | 11.57 | 138.05 | -1.37 | C7H7NO2 | N-Methylnicotinate | S2,S6,S5,S1,S9,S8,S7,S10 |
| 14.34 | 16.44 | 229.16 | 0.75 | C11H20N2O3 | Leu-Pro | S2,S6,S5,S1,S9,S8 |
| 14.34 | 16.96 | 142.09 | -0.54 | C7H11NO2 | L-Hypoglycin | S2,S6,S5,S1,S9,S8,S7,S10 |
| 14.36 | 20.65 | 160.13 | 0.95 | C8H17NO2 | DL-2-Aminooctanoicacid | S2,S6,S5,S1,S9,S8,S7,S10 |
| 14.39 | 11.73 | 159.08 | 0.35 | C6H10N2O3 | 5-Hydroxyectoine | S2,S6,S5,S1,S9,S8,S7,S10 |
| 14.44 | 17.60 | 168.07 | -1.06 | C8H9NO3 | Isopyridoxal | S2,S6,S5,S1,S9,S8,S7 |
| 14.47 | 13.57 | 204.12 | -0.35 | C9H17NO4 | O-Acetylcarnitine | S2,S6,S5,S1,S9,S8,S7,S10 |
| 14.49 | 15.04 | 116.07 | 0.13 | C5H9NO2 | L-Proline | S2,S6,S5,S1,S9,S8,S7,S10 |
| 14.51 | 16.24 | 152.11 | -0.79 | C9H13NO | N-Methylphenylethanolamine | S2,S6,S5,S1,S9,S8,S7,S10 |
| 14.52 | 19.41 | 281.15 | 1.37 | C14H20N2O4 | Val-Tyr | S2,S6,S5,S1,S9,S8,S7,S10 |
| 14.53 | 11.20 | 130.12 | -0.70 | C7H15NO | 4-Trimethylammoniobutanal | S2,S6,S5,S1,S9,S8,S7,S10 |
| 14.56 | 20.69 | 302.09 | -0.71 | C12H15NO8 | 4-Nitrophenol-alpha-D-galactopyranoside | S2,S6,S5,S1,S8,S7,S10 |
| 14.56 | 10.51 | 162.11 | -2.37 | C7H15NO3 | (S)-Carnitine | S2,S6,S5,S1,S9,S8,S7,S10 |
| 14.56 | 18.99 | 188.13 | 0.85 | C9H17NO3 | 8-Amino-7-oxononanoate | S2,S6,S5,S1,S9,S8,S7,S10 |
| 14.60 | 13.30 | 182.08 | -0.86 | C9H11NO3 | L-Tyrosine | S2,S6,S5,S1,S9,S8,S7,S10 |
| 14.66 | 12.27 | 120.08 | -0.24 | C16H18N2 | Agroclavine | S6,S5,S1,S9,S8,S7 |
| 14.70 | 14.75 | 143.08 | 1.39 | C6H10N2O2 | Ectoine | S2,S6,S5,S1,S9,S8,S7,S10 |
| 14.72 | 8.76 | 129.10 | -0.98 | C6H12N2O | L-Lysine 1,6-lactam | S2,S6,S5,S9,S8,S7,S10 |
| 14.74 | 20.29 | 245.19 | 2.13 | C12H24N2O3 | N-(6-Aminohexanoyl)-6-aminohexanoate | S2,S6,S5,S1,S9,S8,S7,S10 |
| 14.80 | 8.31 | 158.12 | -1.13 | C8H15NO2 | Lentiginosine | S2,S6,S5,S1,S9,S8,S7,S10 |
| 14.81 | 21.21 | 188.09 | 1.32 | C8H13NO4 | [FA hydroxy(4:0)] N-(3S-hydroxy-butanoyl)-homoserine lactone | S2,S6,S5,S1,S9,S8,S7,S10 |
| 14.88 | 21.51 | 112.04 | 1.10 | C5H5NO2 | 2,5-Dihydroxypyridine | S2,S6,S5,S1,S9,S8,S7,S10 |
| 14.92 | 15.88 | 118.09 | -0.25 | C5H11NO2 | L-Valine | S2,S6,S5,S1,S9,S8,S7 |
| 14.99 | 14.36 | 152.06 | -0.51 | C5H5N5O | Guanine | S2,S6,S5,S1,S9,S7,S10 |
| 15.00 | 20.07 | 139.05 | -1.90 | C6H6N2O2 | Urocanate | S6,S5,S1,S9,S8,S7,S10 |
| 15.02 | 17.86 | 218.14 | -0.63 | C10H19NO4 | O-Propanoylcarnitine | S6,S5,S1,S9,S8,S7,S10 |
| 15.03 | 14.07 | 467.14 | -1.52 | C15H26N6O7S2 | Asn-Cys-Cys-Gln | S2,S6,S5,S1,S9,S8,S7,S10 |
| 15.27 | 18.90 | 164.11 | -1.19 | C10H13NO | (R)-2-Methylimino-1-phenylpropan-1-ol | S2,S6,S5,S1,S9,S8,S7,S10 |
| 15.32 | 15.06 | 160.10 | -0.15 | C7H13NO3 | 3-Dehydrocarnitine | S2,S6,S5,S1,S9,S8,S7,S10 |
| 15.36 | 16.80 | 332.15 | 0.21 | C18H21NO5 | Pretazettine | S2,S6,S5,S1,S9,S8,S7,S10 |
| 15.42 | 13.43 | 138.09 | -0.69 | C8H11NO | N,N-Dimethylaniline N-oxide | S6,S5,S1,S9,S8,S7,S10 |
| 15.51 | 15.51 | 388.21 | 3.61 | C40H54N8O8 | AngiotensinIV | S6,S5,S1,S9,S8,S7,S10 |
| 15.57 | 21.43 | 124.04 | -0.75 | C6H5NO2 | Picolinic acid | S2,S6,S5,S1,S9,S8,S7,S10 |
| 15.59 | 17.05 | 116.07 | 0.56 | C5H9NO2 | D-Proline | S6,S5,S1,S9,S8,S7,S10 |
| 15.61 | 18.00 | 214.07 | -0.42 | C9H11NO5 | DL-threo-3,4-dihydroxyphenylserine | S2,S6,S5,S1,S9,S8,S7,S10 |
| 15.71 | 14.80 | 136.06 | -0.97 | C5H5N5 | Adenine | S2,S6,S5,S1,S9,S8,S7,S10 |
| 15.72 | 16.94 | 142.05 | 0.13 | C6H7NO3 | 2-Aminomuconate semialdehyde | S2,S6,S5,S9,S8,S7 |
| 15.72 | 21.61 | 166.09 | -2.99 | C9H11NO2 | D-Phenylalanine | S2,S6,S5,S1,S9,S8,S7,S10 |
| 15.88 | 20.28 | 346.17 | 1.03 | C19H23NO5 | Albomaculine | S6,S5,S1,S9,S8,S7,S10 |
| 15.91 | 17.17 | 143.08 | 0.28 | C6H10N2O2 | Ectoine | S2,S6,S5,S1,S9,S8,S10 |
| 15.93 | 17.50 | 144.10 | -0.59 | C7H13NO2 | Stachydrine | S2,S6,S5,S1,S9,S8,S7,S10 |
| 16.00 | 15.04 | 132.07 | -0.16 | C5H9NO3 | 5-Aminolevulinate | S6,S5,S1,S9,S8,S7,S10 |
| 16.34 | 21.67 | 196.06 | -1.93 | C9H9NO4 | 4-Carboxyphenylglycine | S2,S6,S5,S1,S9,S8,S7,S10 |
| 16.42 | 12.37 | 201.16 | 2.54 | C10H20N2O2 | dimethylsuberimidate | S2,S6,S5,S1,S9,S8,S7,S10 |
| 16.63 | 18.10 | 159.08 | -0.68 | C6H10N2O3 | 4-Methylene-L-glutamine | S2,S6,S5,S1,S7 |
| 17.06 | 18.18 | 148.06 | -1.43 | C5H9NO4 | L-Glutamate | S2,S6,S5,S1,S9,S8,S7,S10 |
| 17.86 | 16.68 | 162.11 | -0.46 | C7H15NO3 | L-Carnitine | S2,S6,S5,S1,S9,S8,S7,S10 |
| 18.00 | 15.12 | 118.12 | 0.13 | C6H15NO | 2-Methylcholine | S2,S6,S5,S1,S9,S8,S7,S10 |
| 18.01 | 22.90 | 132.10 | -0.81 | C6H13NO2 | (3R)-beta-Leucine | S2,S6,S5,S1,S9,S8,S7,S10 |
| 18.31 | 22.50 | 150.01 | 4.09 | C7H12N2O7P2 | herbicide 14A | S6,S5,S1,S8,S7,S10 |
| 19.31 | 16.46 | 150.11 | -0.72 | C6H15NO3 | Triethanolamine | S2,S6,S5,S1,S9,S8,S7,S10 |
| 19.40 | 25.47 | 332.15 | 0.15 | C18H21NO5 | Tazettine | S2,S6,S5,S1,S9,S8,S7,S10 |
| 19.83 | 18.65 | 431.24 | 1.71 | C21H30N6O4 | Lys-Phe-His | S2,S6,S5,S1,S9,S8,S7,S10 |
| 20.10 | 20.10 | 205.06 | -0.45 | C19H20O10 | Khellol glucoside | S2,S6,S5,S1,S9,S8,S7,S10 |
| 20.41 | 26.33 | 332.15 | 0.11 | C18H21NO5 | 2-O-Acetylpseudolycorine | S2,S6,S5,S1,S9,S8,S7,S10 |
| 21.18 | 26.84 | 366.14 | -0.26 | C14H23NO10 | 2-(acetylamino)-1-5-anhydro-2-deoxy-3-O-b-D-galactopyranosyl-D-arabino-Hex-1-enitol | S2,S6,S5,S1,S9,S8,S7,S10 |
| 21.38 | 28.88 | 363.17 | 0.69 | C14H26N4O5S | Leu-Cys-Gln | S2,S6,S5,S1,S9,S8,S7,S10 |
| 21.59 | 16.41 | 189.09 | 2.32 | C7H12N2O4 | L-glycyl-L-hydroxyproline | S6,S5,S8,S7,S10 |
| 21.59 | 22.93 | 261.11 | 3.90 | C10H16N2O6 | L-alpha-glutamyl-L-hydroxyproline | S2,S6,S5,S1,S9,S8,S7,S10 |
| 21.60 | 21.60 | 205.06 | -0.69 | C19H20O10 | Khellol glucoside | S2,S6,S5,S1,S9,S8,S7,S10 |
| 21.77 | 22.54 | 322.13 | -1.24 | C32H34N8O7 | Asp-Trp-Trp-His | S7 |
| 25.46 | 23.45 | 170.09 | -0.94 | C7H11N3O2 | N(pi)-Methyl-L-histidine | S2,S1,S9,S8,S7 |
| 25.61 | 24.99 | 156.08 | -0.62 | C6H9N3O2 | L-Histidine | S2,S6,S1,S9,S8,S7,S10 |
| 26.96 | 26.63 | 175.12 | -1.95 | C6H14N4O2 | L-Arginine | S2,S6,S5,S1,S8,S7,S10 |
| 31.56 | 41.03 | 255.07 | 1.54 | C7H15N2O6P | N-&delta;-(phosphonoacetyl)-L-ornithine | S6,S5,S1,S9,S8,S7,S10 |
| 31.94 | 32.18 | 175.14 | -0.18 | C8H18N2O2 | Ne,Ne dimethyllysine | S6,S5,S1,S9,S8,S7 |
| 32.16 | 43.32 | 414.27 | -3.19 | C19H35N5O5 | Ala-Lys-Val-Pro | S2,S6,S5,S1,S9,S8,S7,S10 |
| 32.63 | 42.16 | 330.06 | -3.45 | C10H12N5O6P | 2',3'-Cyclic AMP | S2,S6,S5,S1,S9,S8,S7,S10 |
| 32.84 | 44.68 | 504.35 | 2.73 | C22H45N7O6 | Lys-Lys-Lys-Thr | S2,S1,S9 |
| 32.98 | 44.96 | 634.31 | -0.52 | C31H39N9O6 | Arg-Trp-Trp-Ser | S2 |
| 33.08 | 33.08 | 581.15 | 1.81 | C26H28O15 | Carlinoside | S2,S6 |
| 34.23 | 46.40 | 188.08 | 0.54 | C7H10FN3O2 | &alpha;-fluoromethylhistidine | S6,S5,S8,S7,S10 |
| 34.31 | 49.53 | 220.05 | 3.31 | C6H10N3O4P | 4-Amino-2-methyl-5-phosphomethylpyrimidine | S2,S6,S5,S1,S9,S8,S7,S10 |
| 34.40 | 47.80 | 504.35 | 3.19 | C22H45N7O6 | Lys-Lys-Lys-Thr | S2,S5 |
| 34.61 | 48.21 | 437.19 | 0.40 | C17H24N8O6 | His-Gly-Ser-His | S2,S6,S5,S1,S8,S7 |
| 34.67 | 48.35 | 475.23 | -1.37 | C17H30N8O8 | Ala-Asn-Asp-Arg | S6,S5,S1,S9,S8,S7 |
| 34.83 | 34.83 | 55.94 | -0.46 | Fe | Fe2+ | S2,S6,S5,S1,S9,S8,S7,S10 |
| 34.83 | 48.67 | 590.29 | -2.18 | C27H39N7O8 | Glu-Lys-Trp-Gln | S2,S6,S5,S10 |
| 34.86 | 48.72 | 465.21 | -1.73 | C20H28N6O7 | Asp-Pro-Pro-His | S2,S6,S5,S1,S9,S8,S7,S10 |
| 35.00 | 48.99 | 447.20 | -0.89 | C20H26N6O6 | Gln-Tyr-His | S2,S6,S8,S7 |
| 35.01 | 48.64 | 360.19 | -2.39 | C14H25N5O6 | Leu-Asn-Asn | S2,S6,S5,S1,S9,S8,S7,S10 |
| 35.01 | 49.03 | 463.19 | 0.67 | C20H26N6O7 | His-Gly-Ser-Tyr | S2,S6,S5,S1,S9,S8,S7 |
| 35.02 | 46.94 | 330.06 | -4.02 | C10H12N5O6P | 2',3'-Cyclic AMP | S2,S6,S5,S1,S9,S8,S7 |
| 35.03 | 49.07 | 419.22 | -0.35 | C16H30N6O7 | Ala-Lys-Asn-Ser | S2,S6,S5,S1,S9,S8,S7,S10 |
| 35.06 | 49.13 | 483.22 | -0.39 | C20H30N6O8 | Glu-Thr-Pro-His | S6,S5,S1,S8,S7 |
| 35.08 | 49.16 | 406.17 | -0.25 | C18H23N5O6 | Ser-Tyr-His | S6,S5,S9,S8,S7,S10 |
| 35.13 | 49.27 | 447.26 | 1.30 | C18H34N6O7 | Ala-Lys-Thr-Gln | S2,S6,S5,S1,S9,S8,S7,S10 |
| 35.20 | 49.40 | 634.31 | 2.04 | C31H39N9O6 | Arg-Trp-Trp-Ser | S6,S5 |
| 35.27 | 49.53 | 469.21 | 2.05 | C19H28N6O8 | Asp-Thr-Pro-His | S2,S6,S5,S9,S8,S7,S10 |
| 35.37 | 49.74 | 538.35 | -0.25 | C24H43N9O5 | Arg-Leu-Leu-His | S6,S5,S10 |
| 35.42 | 49.84 | 431.24 | 3.09 | C21H30N6O4 | Lys-Phe-His | S2,S6,S5,S1,S9,S8,S7,S10 |
| 36.18 | 51.36 | 518.29 | -3.90 | C21H39N7O8 | Asp-Lys-Lys-Gln | S2,S6,S5,S1,S9,S8,S7,S10 |
| 36.25 | 49.41 | 330.06 | -4.07 | C10H12N5O6P | 2',3'-Cyclic AMP | S2,S6,S5,S1,S9,S8,S7,S10 |
| 44.63 | 44.63 | 205.06 | -3.26 | C19H20O10 | Khellol glucoside | S2,S6,S5,S1,S9,S8,S7,S10 |
| 45.88 | 45.88 | 948.27 | -3.28 | C33H56N7O17P3S | 2-trans-Dodecenoyl-CoA | S2,S6,S8,S7,S10 |
