# Supplementary material for: Metabolomic and high-throughput sequencing analysis—modern approach for the assessment of biodeterioration of materials from historic buildings
Source: Front Microbiol. 2015 Sep 29;6:979. doi: 10.3389/fmicb.2015.00979 (PMC4586457; doi:10.3389/fmicb.2015.00979)
Supplement: Supplementary file 7 [file Table7.DOCX]

**Table S7**. List of authentic standard used for the calibration of retention time prediction model with ZIC-HILIC chromatography

| **Metabolites** | **RT** | **RT measured** |
| --- | --- | --- |
| **Benzoate** | 5.68 | 5.34 |
| **Fumarate** | 6.21 | 5.75 |
| Pyruvate | 6.73 |  |
| Succinate | 6.73 |  |
| **Uracil** | 8.03 | 8.40 |
| L-Rhamnose | 11.35 |  |
| **L-Phenylalanine** | 11.84 | 12.61 |
| **L-Tryptophan** | 12.26 | 12.77 |
| **L-Leucine** | 12.63 | 13.37 |
| Guanosine | 12.86 |  |
| L-Methionine | 13.11 |  |
| **L-Proline** | 13.96 | 14.49 |
| **L-Valine** | 14.02 | 14.92 |
| D-Gluconic Acid | 14.52 |  |
| D-Glucose | 14.97 |  |
| L-Homocysteine | 15.97 |  |
| **L-Glutamate** | 16 | 17.06 |
| **L-Tyrosine** | 16.03 | 14.60 |
| L-Cysteate | 16.08 |  |
| Taurine | 16.58 |  |
| L-Threonine | 16.66 |  |
| L-Aspartate | 16.69 |  |
| L-Alanine | 18.51 |  |
| L-Glutamine | 19.02 |  |
| Creatinine | 19.1 |  |
| L-Asparagine | 19.47 |  |
| L-Serine | 19.62 |  |
| Glycine | 19.62 |  |
| L-Cystine | 23.22 |  |
| **L-Histidine** | 26.25 | 25.61 |
| **L-Arginine** | 27.29 | 26.96 |
| L-Lysine | 27.67 |  |
| Thiamin | 33.43 |  |
