# Supplementary figures and images for: Metabolomic and high-throughput sequencing analysis—modern approach for the assessment of biodeterioration of materials from historic buildings
Source: Front Microbiol. 2015 Sep 29;6:979. doi: 10.3389/fmicb.2015.00979 (PMC4586457; doi:10.3389/fmicb.2015.00979)

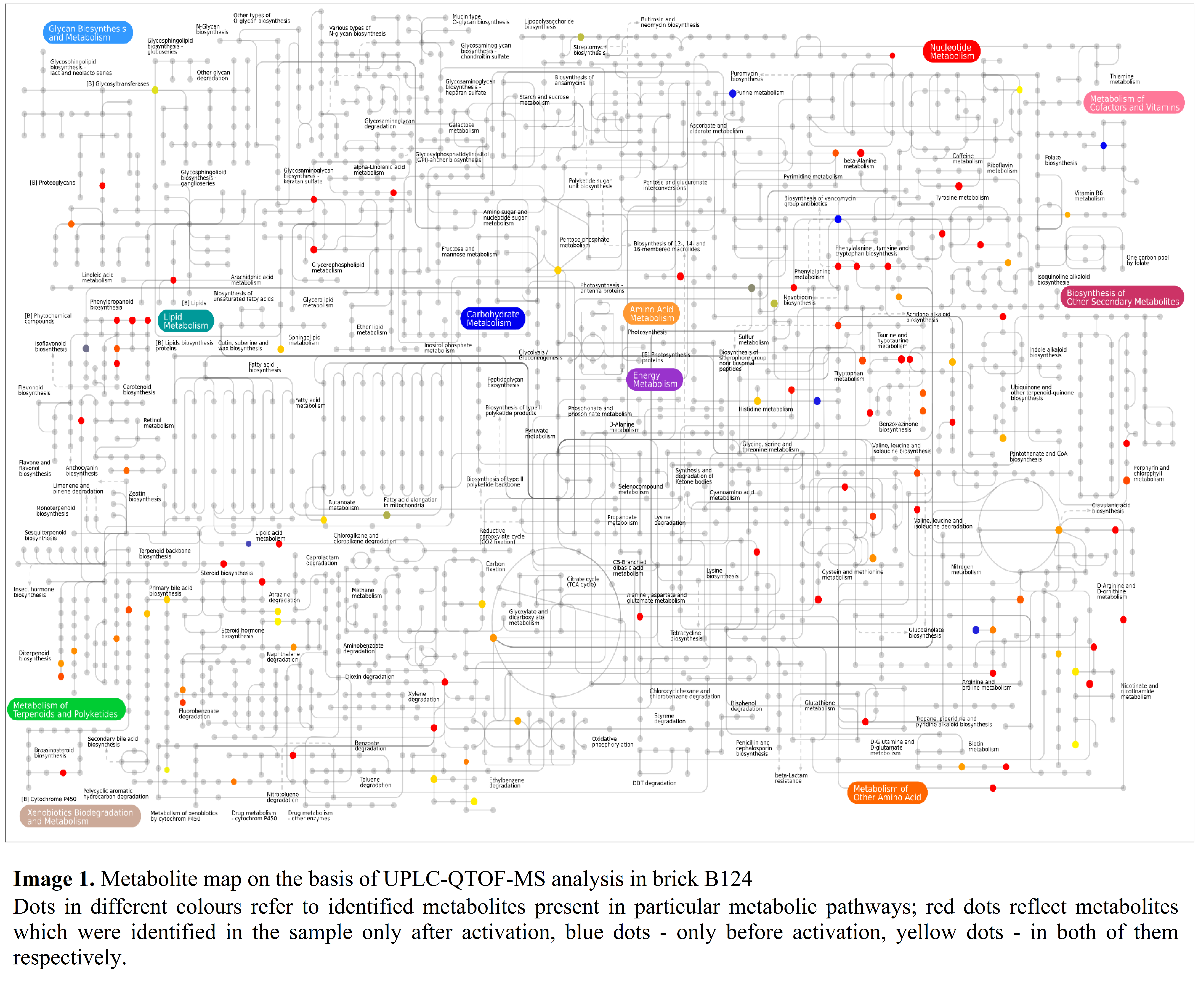

Supplement: Supplementary file 8 [file Image1.TIF]

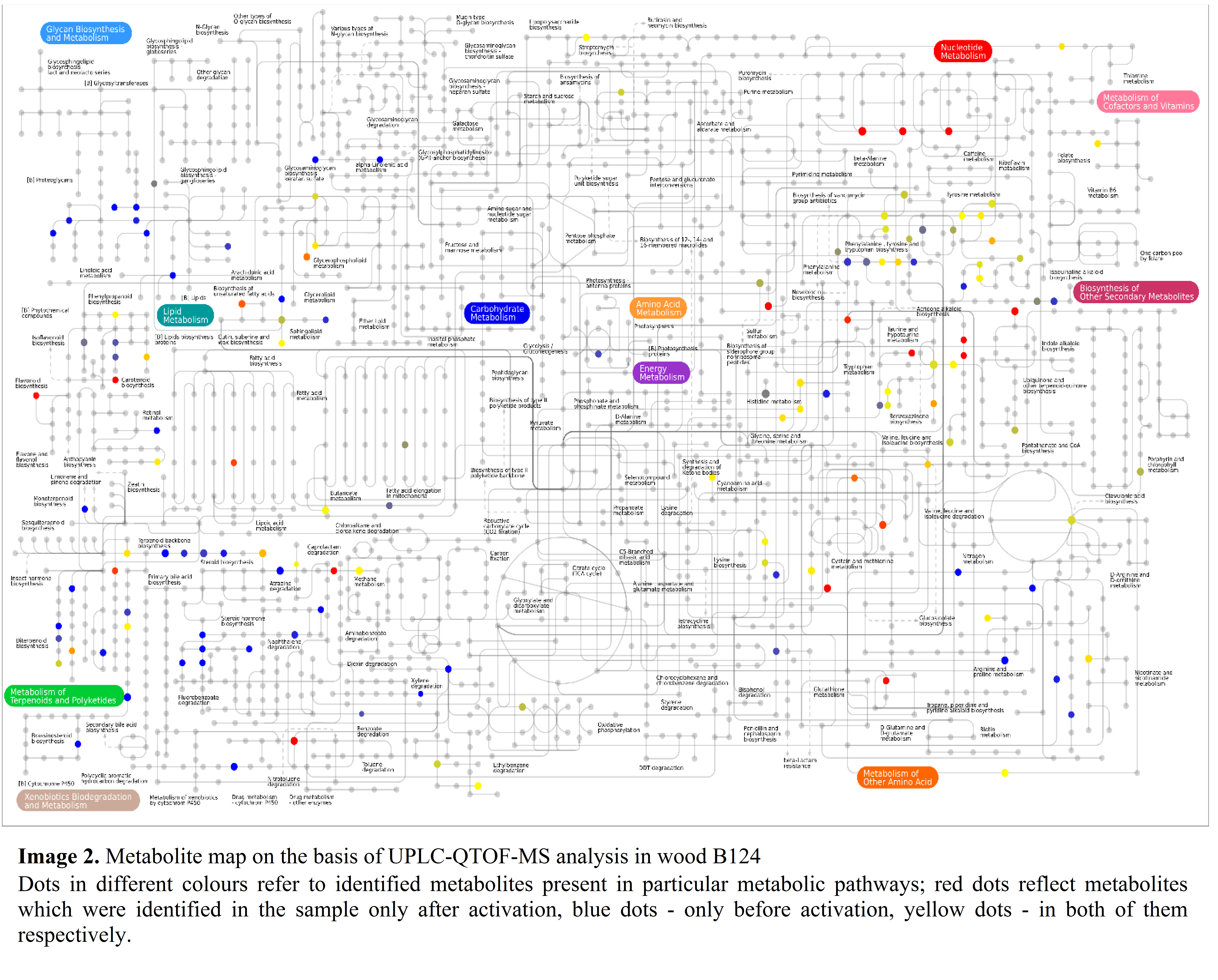

Supplement: Supplementary file 9 [file Image2.TIF]

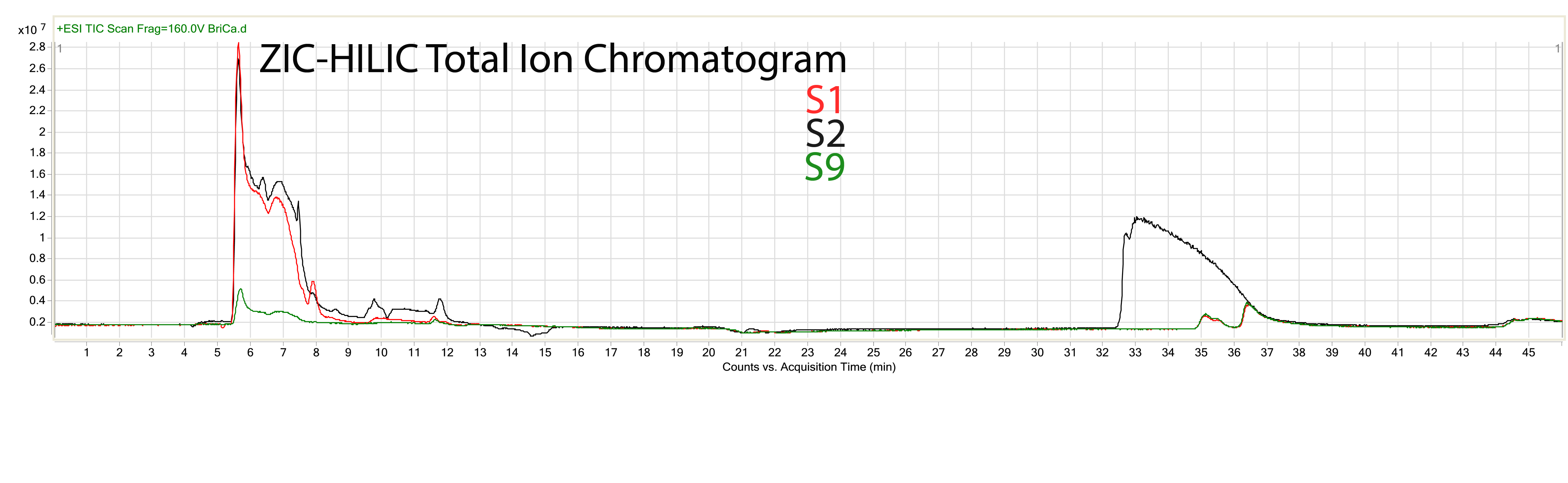

Supplement: Supplementary file 10 [file Image3.TIF]

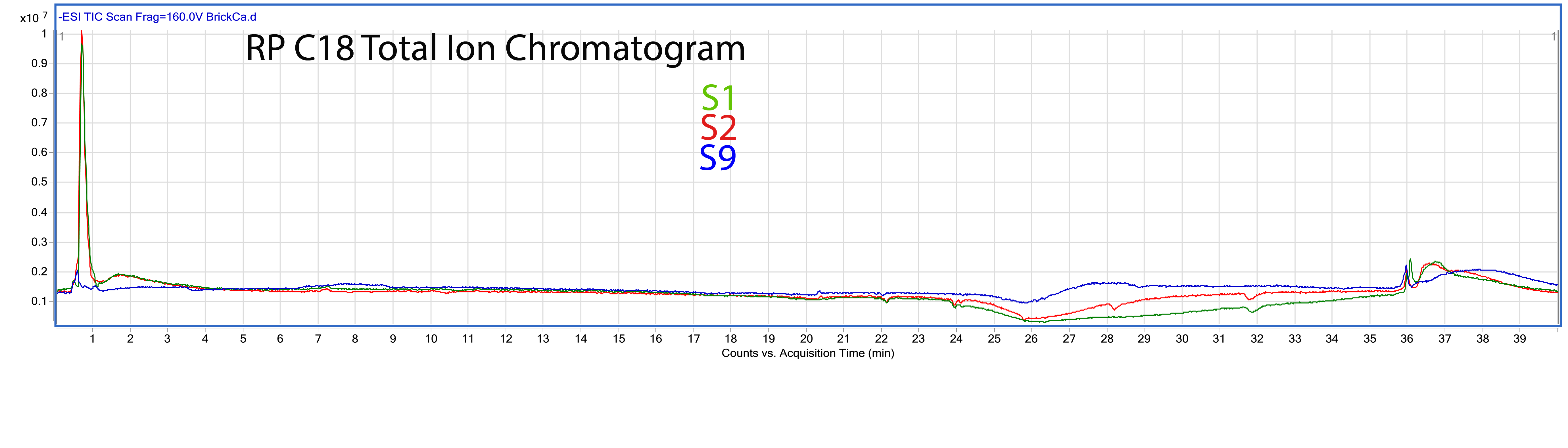

Supplement: Supplementary file 11 [file Image4.TIF]

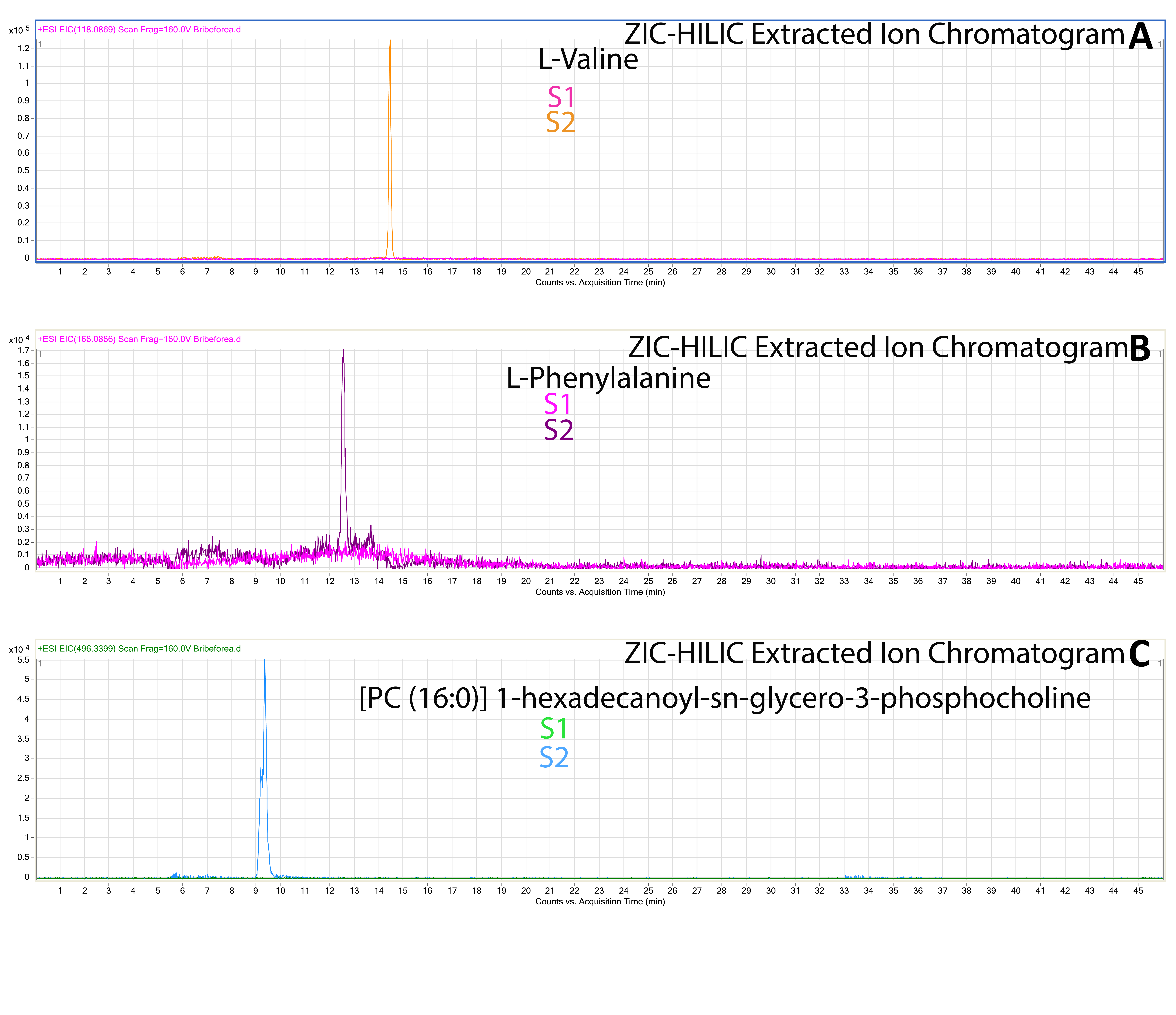

Supplement: Supplementary file 12 [file Image5.TIF]

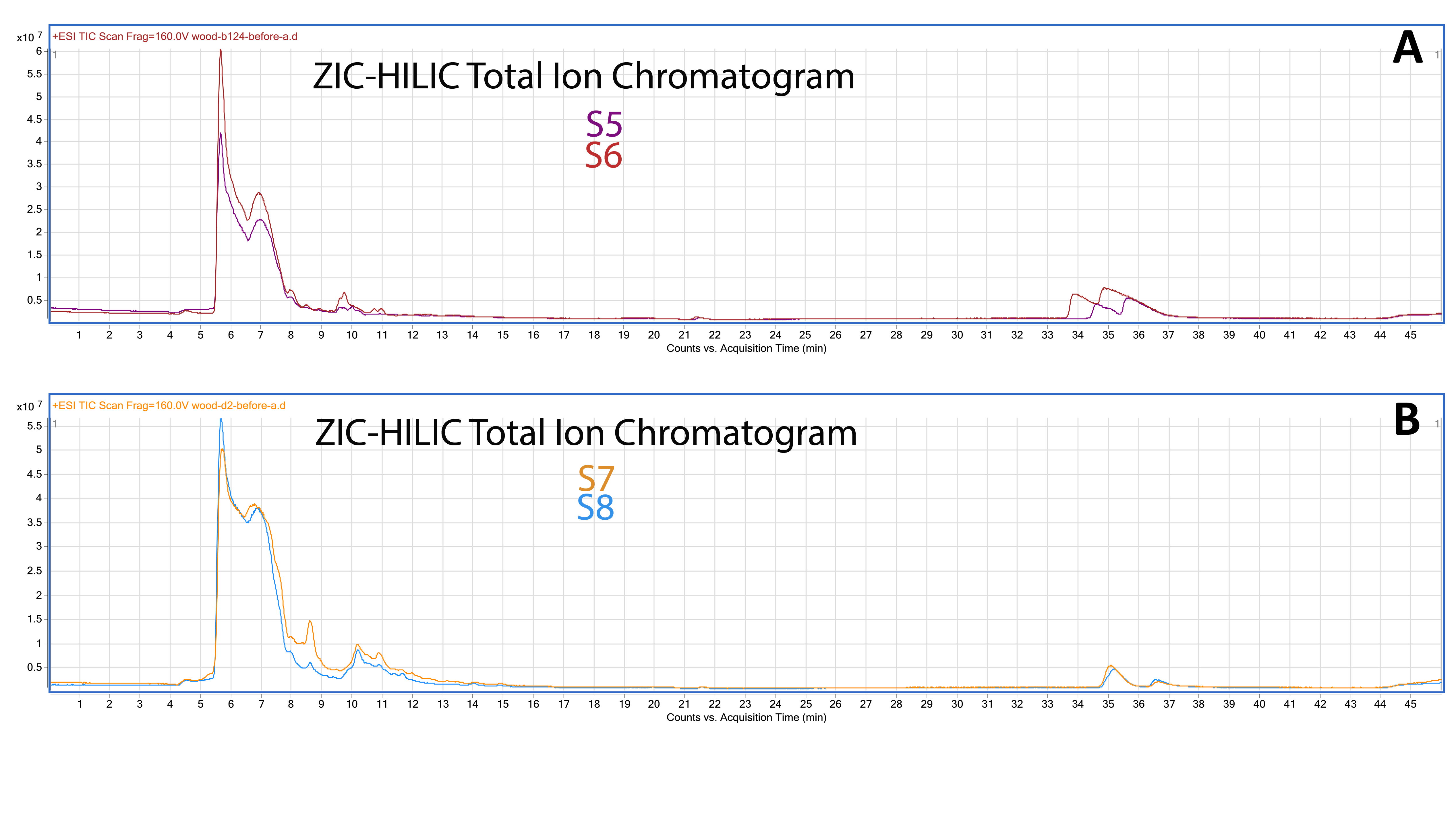

Supplement: Supplementary file 13 [file Image6.TIF]

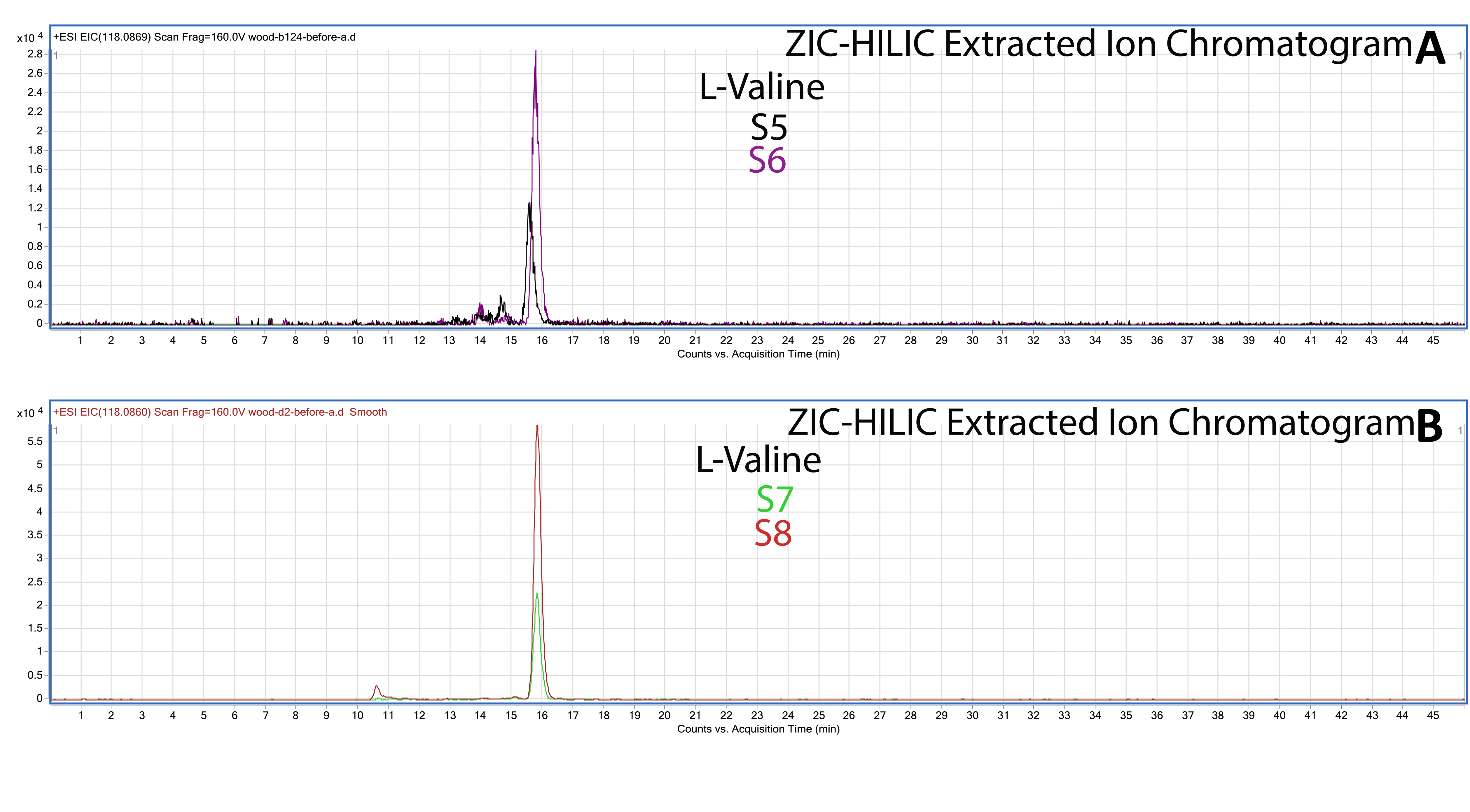

Supplement: Supplementary file 14 [file Image7.TIF]
